# Balance between Crystal Field and Phonon Optimization in Defining the Magnetic Properties of Lanthanide Dichalcogenoimidodiphosphinate Complexes

**DOI:** 10.1021/acs.inorgchem.5c03025

**Published:** 2025-10-27

**Authors:** José Severiano Carneiro Neto, Gemma K. Gransbury, Lucas G. Fachini, Francielli S. Santana, Diego Seckler, Arsen Raza, Patrick Severin Sfragano, Damiano Tanini, Antonella Capperucci, Matteo Briganti, Jaísa F. Soares, Mauro Perfetti

**Affiliations:** † Department of Chemistry “Ugo Schiff”, 9300 University of Florence, Via della Lastruccia 3-13, Sesto Fiorentino 50019, Italy; ‡ Departamento de Química, 28122 Universidade Federal do Paraná, Centro Politécnico, Jardim das Américas, Curitiba-PR 81530-900, Brazil; § Department of Industrial Engineering, DIEF and INSTM Research Unit, University of Florence, Florence 50139, Italy; # Department of Chemistry “Ugo Schiff” and INSTM Research Unit, 9300 University of Florence, Via della Lastruccia 3-13, Sesto Fiorentino 50019, Italy

## Abstract

Single-molecule magnets (SMMs) offer promise for high-density data storage, but suppressing fast magnetization relaxation remains a key challenge in synthetic design. Changing coordinating atoms across a group offers an avenue to control the phononic spectrum and crystal field. We report dysprosium­(III) and erbium­(III) tris­(tetraphenyl-dichalcogenoimidodiphosphinate) complexes with O (**1-Ln**), S (**2-Ln**), or Se (**3-Ln**) donors, examining their influence on the anisotropy and relaxation behavior. The products exhibit distinct geometries, 6-coordinate **1-Ln**, 9-coordinate **2-Ln**, and **3-Ln** complexes that vary from 9-coordinate **3-Tb** to 7-coordinate **3-Er** and **3-Dy**. The geometry dominates the vibrational effects in defining the magnetic relaxation. The 7-coordinate **3-Dy**, with one short Ln–N bond, produces an axial crystal field and the best field-induced SMM behavior (*U*_eff_ = 72(6) cm^–1^), while the high *D*_*3*_ symmetry in **2-Dy** suppresses slow relaxation. Low-lying excited states in **1–3-Er** promote rapid Orbach relaxation, masking Raman processes in **2-Er** and **3-Er**. In **1-Ln**, low-energy vibrations drive Raman relaxation, and contrary to expectations, **3-Dy** exhibits higher vibrational energy contributing to this process. This indicates that heavy chalcogen atoms in the first coordination sphere are a valuable resource, as they can drive crystal field changes that effectively counterbalance the onset of low-energy phonons.

## Graphical abstract

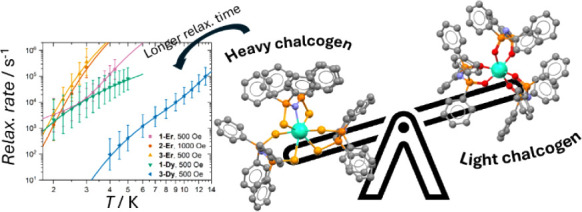

## Introduction

Single-molecule magnets (SMMs) display magnetic bistability and slow relaxation of their magnetization at the molecular level.^1,2^ These systems have garnered significant attention due to their potential applications in high-density data storage and spintronics and their ability to be chemically designed and tuned.^3^ Lanthanide-based SMMs were first reported in 2003,^4^ and the large angular momenta (e.g., Dy^3+^ and Er^3+^, *J* = 15/2 in the ground state) lend the potential for huge uniaxial single-ion magnetic anisotropy and effective barriers to the reversal of the magnetization (*U*_eff_) in the appropriate crystal field (CF).

The general guidelines for maximizing *U*_eff_ and slowing overbarrier Orbach relaxation are well understood: prolate *m*_*J*_ states (*e.g*. *m*_*J*_ = |±15/2⟩ of Er^3+^) are stabilized in an equatorial CF such as in a trigonal planar coordination environment, while oblate *m*_*J*_ states (*e.g*. *m*_*J*_ = |±15/2⟩ of Dy^3+^) are stabilized by an axial CF.^5^ SMMs can also relax via the two-phonon Raman processes, Quantum Tunnelling of Magnetisation (QTM) and one-phonon Direct process in an applied field. Control of all processes is required to improve the SMM performance, and how to suppress relaxation is less well understood for Raman and Direct processes. Theoretical studies on single ion systems suggest that Raman relaxation rates can be slowed by reducing the number of low-energy phonon modes, particularly pseudoacoustic modes, and using rigid polydentate/multihaptic ligands to decouple intramolecular motions from low-energy acoustic vibrations.^6–8^

Experimental studies are needed alongside theory to understand and control relaxation pathways; here we consider the effect of shifting vibrational energies by using different donor elements from the same group. Replacement of coordination atoms can also allow simultaneous tuning of the CF, or significant changes to the CF when the coordination geometry is modified. Computational studies show that substitution of S with Se or Te can be used to tune *U*_eff_ and modulate transverse *g*-values that promote QTM,^9,10^ but the soft donor atom effects are minor as they do not define the magnetic anisotropy axis.^11^ An experimental study on chalcogen-bridged Yb dimers supports this, with only the Te_2_^2–^ species behaving as an SMM.^12^ Substitution of S and O has the potential to significantly change the CF by modifying the anisotropy,^10,13^ and in one trigonal bipyramidal Dy SMM S-substitution was used to improve the symmetry of the equatorial charge distribution, reducing the QTM rate by 3 orders of magnitude and increasing *U*_eff_ from 28.1(9) cm^–1^ (dc field 1000 Oe) to 738(71) cm^–1^ (0 Oe).^14^ In mixed hard–soft donor complexes a single hard anionic donor,^9^ or trans hard anionic donors^14^ can be used to direct the anisotropy. To our knowledge, the effect of donor-atom substitution on Raman rates has not been investigated.

We have chosen tetraorganodichalcogenoimidodiphosphinate ligands, [N­(PR_2_E)_2_]^−^ (*R* = alkyl or aryl, E = chalcogen, Scheme 1) in which the chalcogen atom can be varied. Literature extensively reports on the preparation of the ligands and their metal complexes, encompassing main group^15,16^ and transition (d- and f-block) metals.^17^ By including nitrogen and a range of chalcogen donor atoms^18^ and organic substituents, these chelating molecules significantly enhance the coordination abilities of (related) β-diketonates. Due to their structural versatility, they have been employed to prepare single-source precursors of metal chalcogenides,^19–21^ luminescent materials,^22–27^ NMR chemical shift agents,^28^ and actinoid and lanthanoid complexes from which the nature of chemical bonding in the f-block has been critically reviewed.^29,30^ However, magnetic studies of chalcogenated imidodiphosphinate metal complexes are scarce and limited to d-block elements^31–33^ and [Er­(N­{OP­(C_6_F_5_)_2_}_2_)_3_], which has fast QTM in zero field and is a field-induced SMM with an effective barrier of 18.6 cm^–1^ in a dc field of 500 Oe.^34^ Here we report the synthesis, new crystal structures and magnetic properties of tris­(tetraphenyldichalcogenoimidodiphosphinate) lanthanide complexes [Ln­{N­(*E*PPh_2_)_2_}_3_] with *E* = O (**1-Ln**), S (**2-Ln**) and Se (**3-Ln**), focusing on the archetypal oblate and prolate *4f* shell electron density distribution of the *m*_*J*_ = |±15/2⟩ state in Dy^3+^ and Er^3+^ ions, respectively, with **3-Tb** for structural comparison. We note that the *E* = Te ligand is not synthetically accessible with phenyl substituents.^25^ The denticity of {N­(*E*PR_2_)_2_}^−^ depends on the preferred geometry of the metal atom (particularly in d-block complexes),^35^ the size of the metal cation, the bulkiness of the R-group (frequently phenyl, isopropyl, and *tert*-butyl), and the nature of the metal-chalcogen bond.^25,36^ Bidentate,^37^ tridentate,^38^ and bridging^39^ coordination modes have been reported, sometimes with multiple binding modes in the same series of compounds.^40^ Consequently the lanthanide coordination number (6–9) and geometry varies from octahedral or trigonal prismatic (**1-Ln** ^22,30,41,42^ ), tricapped trigonal prismatic (**2-Ln** ^25,29^ and early **3-Ln** ^25,29,30^ ) to capped trigonal prismatic (reported here for **3-Dy** and **3-Er**), providing the opportunity to investigate the delicate balance between maximizing the CF and tailoring the phononic spectrum.

**Scheme 1:**
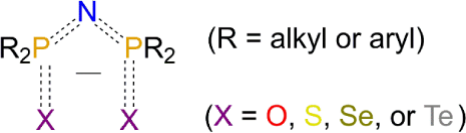
– Dichalcogenoimidodiphosphinate Anions

## Results and Discussion

### Synthesis and Structural Characterization

#### Complexes **1-Ln** with N­(OPPh_2_)_2_^–^ (Tetraphenylimidodiphosphinate, tpip^–^)

The reaction between the metal precursors [Ln­{N­(SiMe_3_)_2_}_3_] (Ln = Er or Dy) and NH­(OPPh_2_)_2_ (Htpip, 1:3 molar ratio in dry thf) was based on the report by Katkova and coworkers,^43^ which described the preparation of the Ce, Nd, Tb, and Ho close analogs of **1-Er** and **1-Dy**. Single crystals were obtained by recrystallization from toluene with X-ray diffraction analysis confirming the formation of the mononuclear tris-chelate complexes (Figure 1a for **1-Er·0.5tol**; Figure S1 for **1-Dy·0.5tol**). Ellipsoid and unit cell plots are displayed in Figures S2 and S3, respectively.

**Figure 1:**
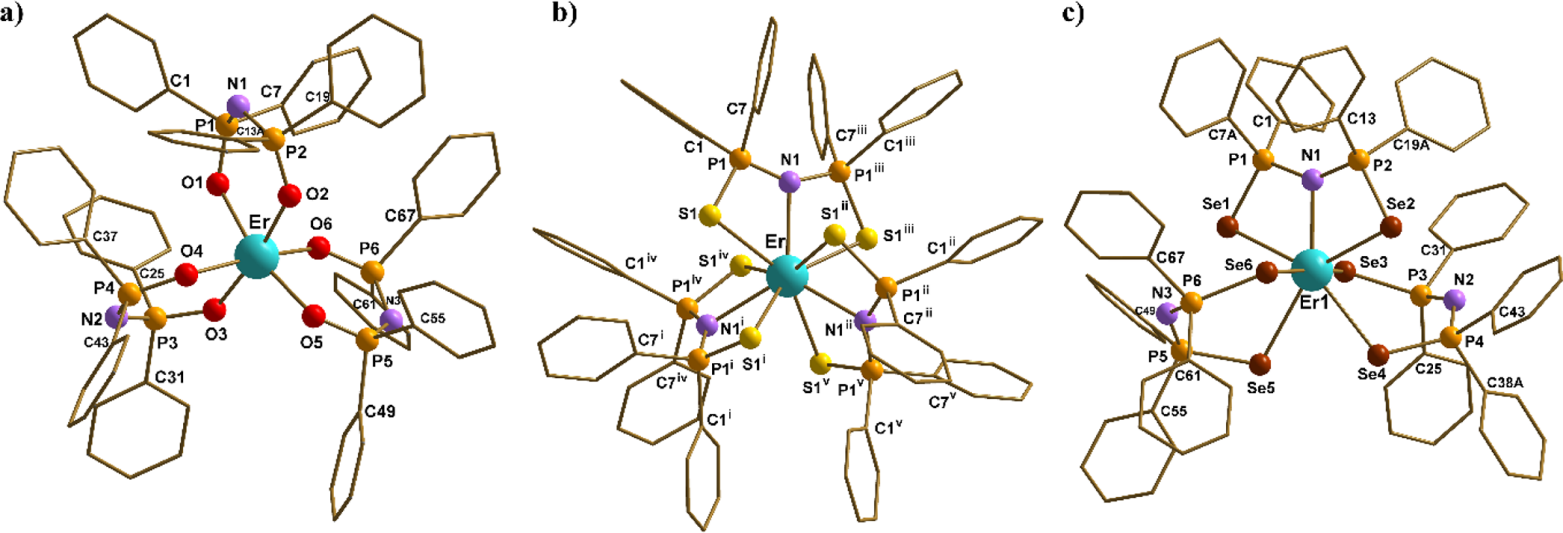
Structures of (a) **1-Er·0.5tol**, [Er­{N­(OPPh_2_)_2_}_3_]•0.5tol, (b) **2-Er·tol**, [Er­{N­(SPPh_2_)_2_}_3_]•tol, and (c) **3-Er·3thf**, [Er­{N­(SePPh_2_)_2_}_3_]·3thf, with the atom numbering scheme. The hydrogen atoms, the second (disordered) orientation of the phenyl rings (C13–C18 and C55–C60 for 1-Er·0.5tol; C7–C12, C19–C24, and C37–C42 for **3-Er·3thf**), and the solvent molecules have been omitted for clarity. The structure of **3-Dy·2thf** is analogous to that of **3-Er·3thf** (Figures S6 and S7).

Collection and refinement data for **1-Er·0.5tol** and **1-Dy·0.5tol** are listed in Table S1; selected bond lengths and angles are shown in Table S2. The structures of **1-Er·0.5tol** and **1-Dy·0.5tol** feature six-coordinate metal centers bound exclusively to the oxygen atoms of three bidentate tpip^–^ ligands, in common with reported **1-Ln** structures for Ln = Pr,^44^ Nd,^30^ Eu,^45^ Gd,^46^ Tb,^22^ Dy,^44,45,47^ Er,^27,48^ Yb,^46^ Lu,^42^ as well as Y.^42^ This differs from the [Ln­{N­(OPPh_2_)_2_}_3_(Solv)] complexes of the light lanthanoids La,^42,49^ Ce,^43^ Pr,^44^ Nd,^27^ and Sm^46^, whose molecular structures display a bound solvent molecule (Solv = thf, MeCN, ethyl acetate, acetone, water). None of the ligand N atoms bind the metal, probably because the small Ln–O bonds and O–Ln–O bite angles (Tables 1 and S2) prevent access to the Ln cation.

**Table 1:** Selected Average Bond Lengths (Å) and Angles (°) for Dichalcogenoimidodiphosphinate Complexes of Er^3+^ and Dy^3+^ (E = Chalcogen)^a^ ^b^ ^c^

	1-Er·0.5tol	1-Dy·0.5tol	2-Er·tol	2-Dy·tol	3-Er·3thf	3-Dy·2thf	3-Er·dcm	3-Dy·dcm
**Ln–E**	2.2409(14)	2.271(3)	2.9121(5)	2.9236(3)	2.9123(9)^c^	2.9306(4)^c^	2.8915(10)^c^	2.9132(3)^c^
**Ln–N** (κ^3^	-	-	2.5421(17)	2.5545(14)	2.412(6)	2.399(3)	2.396(7)	2.4264(18)
**Ln**•••**N** (κ^2^	3.868	3.895	-	-	4.204	4.166	4.252	4.268
**P–N**	1.5913(17)	1.588(4)	1.6125(6)	1.6111(5)	1.605(7)	1.608(3)	1.603(7)	1.608(2)
**P–E**	1.5226(14)	1.519(3)	1.9847(6)	1.9831(4)	2.158(2)	2.1586(10)	2.160(2)	2.1609(6)
**Ln•••Ln** ^b^	11.137	10.833	12.753	12.728	12.368	12.199	13.004	13.029
**E–Ln–E** (κ^3^	-	-	125.954(16)	125.564(12)	133.50(3)	132.750(12)	132.35(3)	131.859(8)
**E–Ln–E** (κ^2^	82.90(5)	81.81(12)	-	-	87.07(3)	86.970(13)	88.08(3)	87.859(8)
**N–Ln–E** (κ^3^	-	-	76.148(8)	76.270(6)	66.90(14)	66.46(7)	67.91(16)	67.59(4)
**P–N–P** (κ^3^	-	-	147.07(12)	147.35(10)	141.2(4)	139.3(2)	140.2(5)	140.82(12)
**P–N–P** (κ^2^	126.49(11)	127.3(3)	-	-	131.8(5)	130.1(2)	131.5(5)	130.68(13)

a Data collection was performed at 100 K, except for **1-Dy·0.5tol** (298 K) and **3-Er·3thf** (300 K).

b Shortest distances in the unit cell.

c Includes both bidentate and tridentate ligands.

Isostructural **1-Er·0.5tol** and **1-Dy·0.5tol** crystallize in the triclinic system (*P*1̅), with two tris-chelate and one solvating toluene in the unit cell. This space group matches the one reported for the Yb^3+^ and Gd^3+^ analogues, also crystallized from toluene.^46^ The **1-Er·0.5tol** structure is also like the one for **1-Er** reported by Ye and co-workers^48^ without cocrystallized solvent. However, it differs from the higher-symmetry space groups reported for **1-Er·0.5H**_**2**_ **O** (*R*3̅, trigonal), grown from MeCN,^27^ and **1-Dy** ^48^ crystallized from dcm/ethanol in the *P*3̅(trigonal) space group.^47^ The use of toluene as the recrystallization solvent led to a much lower crystal symmetry but only one crystallographically independent distorted octahedral complex in the unit cell (Tables 1 and S3), simplifying the interpretation of the magnetic measurements (see below).^50^ In the previously reported trigonal space groups, two (*R*3̅) and three (*P*3̅) crystallographically independent molecules are observed, distorted octahedral and trigonal prismatic (Table S3), in which each lanthanoid cation resides on a crystallographic *C*_3_ axis.

Bond lengths and angles involving the *O-*donor atoms and the metal centers (average Ln–O bond of 2.2409(14) for Er and 2.271(3) Å for Dy at 100(2) K and 298(2) K respectively, and O–Ln–O angles of 82.90(5)° and 81.81(12)° for Er and Dy respectively, are consistent with those reported in the literature. Short average N–P bond lengths of 1.5913(17) and 1.588(4) Å for **1-Er·0.5tol** and **1-Dy·0.5tol** (respectively), compared to the 1.62–1.71 Å range for a single N–P bond, and P–N–P angles of 126.49 (11) and 127.3(3)° (Tables 1 and S2) support a substantial sp^2^ character at the nitrogen centers (122–133° range, Scheme 1).^2^ Phosphorus–oxygen bond lengths of 1.5226(14) (**1-Er·0.5tol**) and 1.519(3) Å (**1-Dy·0.5tol**), in turn, are significantly larger than the known 1.44–1.49 Å range for = O bonds,^15^ evidencing the electron-withdrawing effect of metal coordination.

#### Complexes **2-Ln** with N­(SPPh_2_)_2_^–^

Adapting a literature procedure,^29^ a solution of [Ln­{N­(SiMe_3_)_2_}_3_] (Ln = Er^3+^, Dy^3+^) in toluene was carefully layered on top of a thf solution of NH­(SPPh_2_)_2_ to form a liquid bilayer (1.5:1 toluene:thf). After 48 h at room temperature in the glovebox, crystals were isolated by filtration and carefully dried under vacuum to avoid crystallization solvent loss, yielding **2-Er·tol** and **2-Dy·tol**. Single-crystal X-ray diffraction analyses of the two products confirmed the formation of the desired mononuclear, homoleptic, neutral complexes (Figures 1b, S4 and S5). Collection and refinement data, bond angles, and bond lengths are summarized in Tables S4 and S5. Average bond dimensions are presented in Table 1 and compared with those for the analogous complexes with the O- and Se-donor ligands.

In contrast to the structures of **1-Er** and **1-Dy**, products **2-Er·tol** and **2-Dy·tol** contain nine-coordinate metal cations (Figure 1). The structure of **2-Dy·tol** has been briefly reported but not discussed,^25^ while the Er^3+^ product is described here for the first time. Previously reported **3-Ln** complexes with the larger tripositive lanthanoids La, Ce, Pr, Nd, Sm, Eu, Gd, and Tb, and with Y, also crystallize in the *R*3̅*c* space group and are isostructural with **2-Er·tol** and **2-Dy·tol** ^**25**^ Each lanthanoid center lies on a proper rotation *C*_3_ axis perpendicular to three *C*_2_ axes that coincide with the Ln–N bonds. This confers a *D*_3_ point symmetry to the **2-Er** and **2-Dy** complexes (and **3-Tb**, see below), the highest symmetry observed among the products described in this work. The tridentate ligands coordinate the Ln^3+^ cation through two sulfur and one nitrogen donor atoms, resulting in a tricapped trigonal prismatic geometry. This shows that the steric restrictions to the coordination of the nitrogen atoms, imposed by the short Ln–O bonds in **1-Er** and **1-Dy**, are lifted in the sulfur-containing products due to the longer covalent radii (0.66(2) Å for oxygen and 1.05(3) Å for sulfur).^29^ Indeed, the average Ln–S bonds, 2.9121(5) (**2-Er·tol**) and 2.9236(3) Å (**2-Dy·tol**), are *ca* 30% longer than the corresponding figures in the *O-*bound complexes (Table 1), as are the P–S bond distances compared to P–O (1.9839(5) versus 1.521(2) Å, respectively). The average internal angles in each chelate ring (E–Ln–E and P–N–P, E = chalcogen, Table 1) reflect the inner ring strains determining the distinct coordination geometries. As an example, the average S–Ln–S angle in **2-Er·tol** and **2-Dy·tol**, 125.759(14)°, is much larger (>1.5 times) than the corresponding O–Ln–O figure in **1-Er·0.5tol** and **1-Dy·0.5tol** (82.36(9)°), allowing for the coordination of the N atoms.

#### Complexes **3-Ln** with N­(SePPh_2_)_2_^–^

Products **3-Er** and **3-Dy** were prepared by a synthetic route analogous to that employed for **2-Er** and **2-Dy** (see Experimental) and crystallized from a similar thf/toluene mixture. However, unlike the complexes obtained with *O,O-* and *S,N,S-*donor ligands, the unit cells of **3-Er** and **3-Dy** contain thf instead of toluene as the solvating molecule. The same synthetic procedure employed for **3-Er·3thf** and **3-Dy·2thf** provided complex **3-Tb** (trigonal, *R*3̅*c*) for structural comparison, but in this case, the unit cell accommodates one solvating toluene molecule per unit of the complex (Tables S10 and S11). These three Se-containing products are new and described here for the first time. Additionally, to obtain larger crystals with improved morphology, recrystallizations were performed in dichloromethane to obtain **3-Er·dcm** and **3-Dy·dcm** with the same *P*2_1_/*n* (monoclinic) space group but different cell volumes (Tables S6–S9).

Single-crystal X-ray diffraction analyses of **3-Er**, **3-Dy**, and **3-Tb** gave surprising results (Figure 1 and S6–S9). Complexes **3-Er·3thf** and **3-Dy·2thf** are isostructural and feature a seven-coordinate metal center bound to three ligand anions: two bidentate, coordinated only by selenium atoms, and one tridentate, which additionally coordinates to the lanthanoid via the nitrogen atom. Conversely, **3-Tb·tol** is nine-coordinate and adopts a tricapped trigonal prismatic geometry^50^ (*D*_3_ symmetry) with crystal structure isomorphic to **2-Ln·tol** (Figures 1 and S8) and **3-Ln·tol** (Ln = La–Gd).^25,29^

It thus appears that the Er–Se and Dy–Se bonds are not long enough (average 2.9306(4) Å in **3-Dy·2thf** compared to 2.9236(3) Å in the sulfur-containing **2-Dy·tol** (Table 1), to allow all three nitrogen atoms to bind the Ln cation before electronic repulsion involving the Se atoms and the phenyl groups becomes prohibitive. In this context, Stewart and coworkers reported that their attempts to crystallize **3-Tb** by the same method that gave the Pr, Nd, Sm, and Gd analogs were unsuccessful.^25^ It is then possible, considering the intermediate effective ionic radius of Tb^3+^ (109.5 pm for 9-coordination) between Gd^3+^ (110.7 pm) and Dy^3+^ (108.3 pm),^29^ that tripositive terbium marks the critical transition point between nine-coordination in [Gd­{N­(SePPh_2_)_2_}_3_][Bibr ref26] and seven-coordination in **3-Dy**. This agrees with the fact that 9-coordinate [Ln­{N­(SePPh_2_)_2_}_3_] molecules are reported for La,^29^ Ce,^29^ Nd,^30^ Sm^25^ and Gd^25^ and now Tb, while 7-coordination sets in with Dy, Er (this work), and Y.^36^ Seven-coordinate Dy^3+^, Y^3+^, and Er^3+^ have effective ionic radii of 97, 96, and 94.5 pm,^51^ respectively. This change in coordination number and ligand denticity is apparent when Ln–Se and Ln–N bond lengths in **3-Ln** are plotted (Figure 2), where an abrupt decrease in bond distances between Tb^3+^ and Dy^3+^ reflects the ongoing structural reorganization. Powder X-ray diffraction results confirm that bulk samples of **3-Er·3thf** and **3-Dy·2thf** are the phase pure monoclinic *P*2_1_/*n*, with no evidence of a trigonal *R*3̅*c* phase (Figure S10).

**Figure 2:**
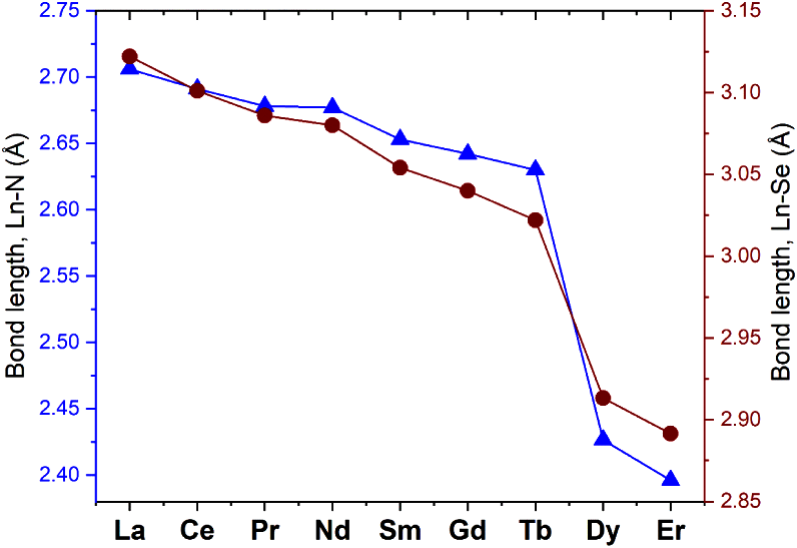
Ln–Se and Ln–N bond lengths for **3-Ln** complexes. Data for La–Gd are those from the literature,^25,29^ while those for the novel products **3-Er**, **3-Dy**, and **3-Tb** are reported in this work. Complexes from La to Tb are 9-coordinate and isostructural, while Dy^3+^ and Er^3+^ give 7-coordinate products. Tris-chelate complexes of tripositive Eu, Ho, and Tm–Lu have not yet been reported.

The observation of 7-coordinate complexes for the later members of the **3-Ln** series but not **2-Ln** is attributed to the comparatively small increase in covalent radius from S to Se (1.05(3) to 1.20(4) Å)^52^ due to the poor shielding of the nuclear charge by the *3d* electrons. It is likely that the large and diffuse Se electron cloud creates strong repulsion near the metal center, distancing two of the three nitrogen atoms (4.2–4.3 Å). The combined effect of two more flexible chelate rings with the increased Se–Ln–Se bite angle allows the nitrogen donor-atom to closely approach the Ln cation resulting in a substantially shorter Ln–N1 bond (2.4 Å) in **3-Er** and **3-Dy** than in the corresponding **2-Ln** complexes (Figures 1c and S6, and Table 1). The resulting geometry is a distorted capped trigonal prism (highest possible symmetry *C*_*2v*_) with an approximate *C*_2_ axis in each molecule that coincides with the Ln–N1 bond. The consequences of these structural reorganizations on the magnetic properties are discussed below.

### Infrared Spectroscopy

Complexes **1-Ln**, **2-Ln** and **3-Ln** (Ln = Er, Dy) have been characterized by FTIR spectroscopy and their spectra are compared with the proligands (Figures S13, S14 and S16). The FTIR spectra of **1-Er** and **1-Dy** are consistent with the presence of the N­(OPPh_2_)_2_^–^ (tpip^–^) ligand, including vibrational modes at 1088 and 1063 cm^–1^, attributed to ν­(P–O),^53^ and the strong band at 1213 cm^–1^, assigned to antisymmetric ν­(P_2_N).^47,54^ In Htpip, the very broad absorption centered at *ca* 760 cm^–1^, absent in the HStpip and HSetpip analogs, comes from the predominant O=PPh_2_–N=PPh_2_(O–H) tautomer,^55^ feature in which the potential *O,O-*donor Htpip differs from the other tetraphenyldichalcogenoimidodiphosphorus acids (compare Figures S13, S14 and S16).^56^ There are no vibrational modes between 3100 and 4000 cm^–1^, confirming the absence of water in the crystals.

In the FTIR spectra of **2-Er** and **2-Dy**, the absence of the vibrational modes at 2631 and 1325 cm^–1^, assigned respectively to ν­(N–H) and δ­(N–H), both present in the proligand (HStpip), confirms ligand deprotonation.^53^ The strong bands at *ca* 1200 cm^–1^, assigned to ν_as_(P_2_N), also corroborate the presence of deprotonated imidodiphosphorus ligands compared to the intense and broad absorption at *ca* 920 cm^–1^ due to ν_as_(P_2_NH) in the free HStpip acid.^56^ The increased wavenumber in the deprotonated molecule reflects the higher PN bond order. Additionally, the strong band at 648 cm^–1^ in the proligand, attributed to ν_as_(PS), shifts to 595 cm^–1^ in the spectra of **2-Er**, **2-Dy**, and **2-Tb** (the latter prepared for comparison) due to coordination (Figures S14 and S15). A decrease in ν_as_(PS) of *ca* 70 cm^–1^ on coordination to first-row transition metals was reported by Siiman and Vetuskey,^53^ and of 50 cm^–1^ on going from H­(Stpip) to Na­(Stpip). The latter, similar to the observed for **2-Er**, **2-Dy**, and **2-Tb**, is compatible with the predominantly ionic nature of the S–Ln bond.

In the FTIR spectra of **3-Er** and **3-Dy** the absorptions at 1203–1205 and 1098–1100 cm^–1^, attributed to ν_as_(P_2_N), and 589 cm^– 1^, ν­(P–Se), confirm the successful preparation of the desired complexes. In addition, the high sensitivity of **3-Er** and **3-Dy** to hydrolysis provided additional evidence of coordination (Figure S17). After 10 min of exposure to the air, two intense bands at 1322 cm^– 1^, attributed to the δ­(N–H), and 917 cm^– 1^, ν_as_(P–NH), emerged in the infrared spectrum of **3-Er**, together with a weak absorption at 2622 cm^– 1^, assigned to ν­(N–H). All these bands belong to the protonated proligand, HSetpip, formed in the Nujol mull upon hydrolysis of the complex.^53^

### Static Magnetic Properties

Solid-state magnetic data were obtained for microcrystalline samples of **1-Ln·0.5tol**, **2-Ln·tol** (Ln = Dy, Er), **3-Er·dcm** and **3-Dy·2thf** to explore their magnetic properties. The magnetic susceptibility-temperature products (χ*T*) at 300 K and 0.1 T are 11.49 (**1-Er·0.5tol**), 11.77 (**2-Er·tol**), 11.25 (**3-Er·dcm**), 13.58 (**1-Dy·0.5tol**), 14.15 (**2-Dy·tol**) and 14.37 cm^3^ K mol^–1^ (**3-Dy·2thf**), in reasonable agreement with the expected free ion values of 11.48 and 14.17 cm^3^ K mol^–1^ for Er^3+^ and Dy^3+^, respectively (Figure 3). Compounds **1-Er·0.5tol** and **2-Er·tol** show a slow decrease in *χT* with decreasing temperature as the crystal field states are depopulated, with a more rapid decrease below 100 K, attaining values of 4.87 and 4.53 cm^3^ K mol^–1^ at 2 K. In contrast, **3-Er·dcm** only significantly decreases below 30 K, suggesting a smaller overall crystal field splitting (CFS), and arriving at 8.43 cm^3^ K mol^–1^ at 2 K. For the Dy^3+^ complexes, values of *χT* decrease more significantly below 100 K, reaching values of 9.76 (**1-Dy·0.5tol**), 7.89 (**2-Dy·tol**) and 11.35 cm^3^ K mol^–1^ (**3-Dy·2thf**) at 2 K, 0.1 T, suggesting that the derivative with the most magnetic ground state is **3-Dy·2thf**, and least magnetic is **2-Dy·tol**.

**Figure 3:**
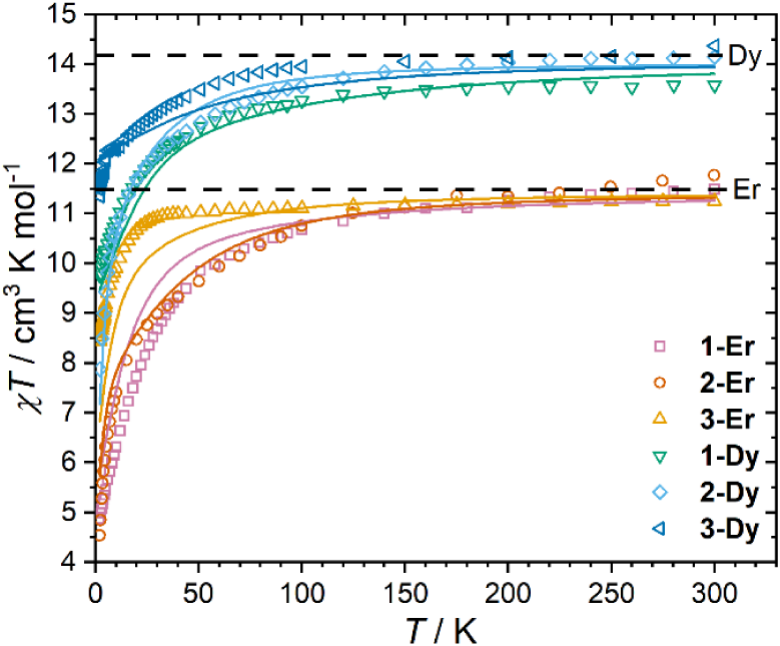
Temperature-dependence of χ*T* for **1-Ln·0.5tol**, **2-Ln·tol**, **3-Er·dcm** and **3-Dy·2thf**. Solid lines indicate CASSCF-SO calculated curves. Dashed black lines indicate free ion values for Dy^3+^ and Er^3+^.

The magnetization of the Er^3+^ complexes does not saturate even at 5 T at 2 K (Figure S19), reaching 4.55 (**1-Er·0.5tol**), 4.73 (**2-Er·tol**) and 5.35 *N*_A_ μ_B_ (**3-Er·dcm**); **1-Er·0.5tol** and **2-Er·tol** are close to the expected value for a pure *m_J_* = |±15/2⟩ state (4.50 *N*_A_ μ_B_), suggesting significant *m_J_* = |±15/2⟩ contributions. The reduced magnetization curves of the Er^3+^ compounds (Figures S19, S21 and S23) do not overlap, indicating low-lying accessible excited states. The magnetization for **1-Dy·0.5tol** and **3-Dy·2thf** (Figure S31) is close to saturating at 2 K and 5 T at values of 5.28 and 5.51 *N*_A_ μ_B_, respectively, suggesting significant *m_J_* = |±15/2⟩ contributions as a pure *m_J_* = |±15/2⟩ would saturate at 5.00 *N*_A_ μ_B_. The reduced magnetization curves are overlapped at low *H*/*T* for **3-Dy·2thf** (Figure S29) and close to overlapped for **1-Dy·0.5tol** (Figure S25), suggesting these compounds have relatively well isolated ground states. In contrast, **2-Dy·tol** (Figure S27) does not saturate, reaching 6.50 *N*_A_ μ_B_ at 2 K, 5 T, and the reduced magnetization curves do not overlap, indicating at least one low-lying excited state.

### CASSCF-SO Calculations

To understand the impact of the different coordination environments on the electronic structure of **1–3-Ln**, complete active space self-consistent field spin–orbit (CASSCF-SO) calculations were performed on the crystal structure coordinates (Figures 4, 5, S33–S40, Tables 2 and S16–S25). The agreement of calculated and experimental susceptibility and magnetization curves is good for almost all the compounds (Figure S18–S32) except for overpredicted high-field magnetization values for **2-Er·tol** (Figure S21), underpredicted magnetic susceptibility below 100 K for **3-Er·dcm** (Figure S23), and the trend of the magnetic susceptibility of **2-Dy·2thf** (Figure S28).

**Table 2:** CASSCF-SO Results for **1-Ln**, **2-Ln**, and **3-Ln** Complexes, Showing *g*-Tensor Components, Ground State Composition, Expectation Value of the *J*_*z*_ Operator for the Ground Doublet, Energy Gap to the Second Kramers Doublet and Total Crystal Field Splitting

	Ground state						
	*g*_*z*_	*g*_*y*_	*g*_*x*_	Composition including contributions >4%	<*J*_*z*_>	2nd KD/cm^–1^	CFS/cm^–1^
**1-Er·0.5tol**	11.59	5.84	1.56	45%|±15/2> + 19%|±11/2> + 5.7%|±5/2> + 5.6%|±3/2> + 4.7%|±1/2> + 4.5%|∓3/2>	±5.80	29.4	347
**2-Er·tol**	12.26	0.23^a^	0.21^a^	29%|±15/2> + 29%|±9/2> + 20%|∓9/2> + 12%|∓15/2> + 7.6%|∓3/2>	±6.13	8.9^b^	236
**3-Er·3thf**	14.05	3.33	1.39	40%|±15/2> + 30%|∓15/2> + 7.7%|±9/2>	±7.02	12.4	232
**3-Er·dcm**	13.66	3.36	0.20	51%|±15/2> + 24%|±11/2> + 5.0%|±7/2> + 4.0%|±13/2>	±6.83	16.0	220
**1-Dy·0.5tol**	17.16	1.31	0.82	80%|±15/2> + 4.1%|±1/2>	±8.58	47.0	478
**2-Dy·tol**	3.05	9.14^*a*^	9.45^*a*^	60%|±7/2> + 39%|∓5/2>	±1.52	11.2	147
**3-Dy·2thf**	19.68	0.043	0.025	94%|±15/2> + 3.0%|∓15/2>	±9.84	120	461
**3-Dy·dcm**	19.48	0.14	0.067	71%|±15/2> + 23%|∓15/2> + 4.0%|±11/2>	±9.74	88.1	364

a By symmetry, g_x_ and g_y_ should be equal; any variation is due to loss of symmetry by rounding of coordinates in calculations.

b Scaled value to fit magnetization data is 19 cm^–1^.

**Figure 4:**
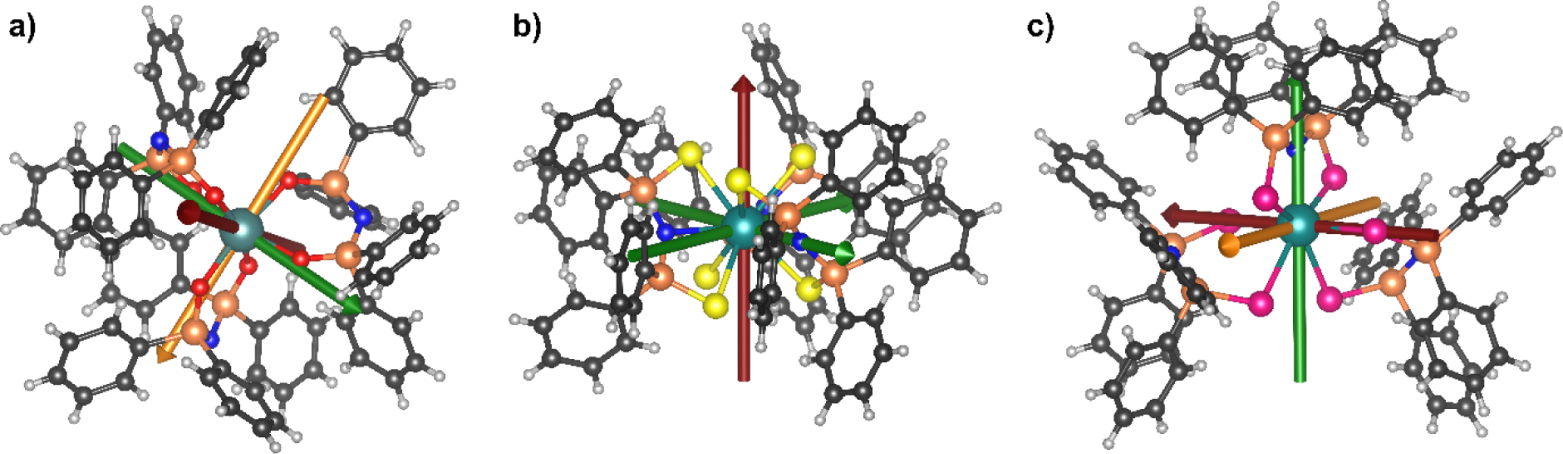
Ground-state anisotropy axes for (a) **1-Dy·0.5tol**, (b) **2-Dy·tol** and (c) **3-Dy·2thf** as calculated by CASSCF-SO. Easy axis (green), intermediate axis (orange), hard axis (maroon). Color code: Dy (cyan), N (blue), P (peach), O (red), S (yellow), Se (pink), C (dark gray), and H (light gray).

**Figure 5:**
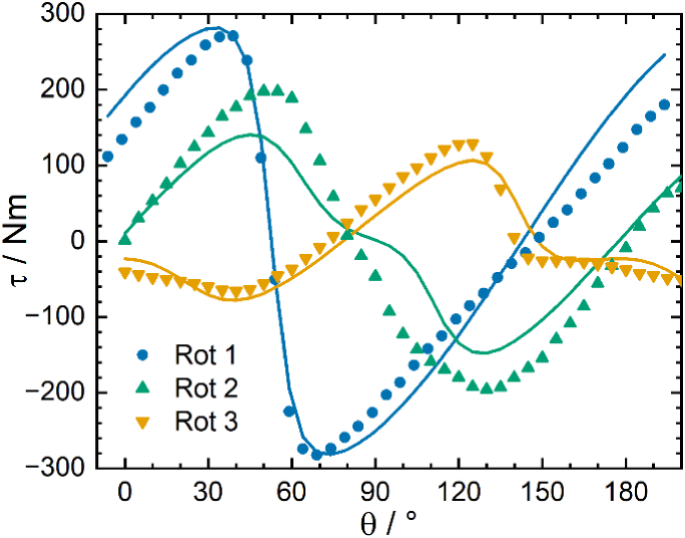
Cantilever torque magnetometry measurements of **3-Er·dcm** at 2 K, 9 T, showing three perpendicular rotations, along with simulations using a *S*_eff_ = 1/2 model.

The ground states are extremely varied because of the different coordination environments in **1–3-Ln** (Table 2). In **1-Ln**, the coordination environment is pseudo-octahedral (local C_1_ symmetry) with hard oxygen donors, so there is no strong driving force or geometric constraints defining the magnetic anisotropy axis. Consequently, for **1-Er·0.5tol** the ground state is rhombic with the easiest axis bisecting the O–Er–O angle of one of the bidentate ligands (Figure S41; 4.8° between *g*_*z*_ and Er–N) while for **1-Dy·0.5tol** the ground state is rhombic but more axial, with the easy axis aligning along one of the O–Dy–O axes of the pseudo-octahedron (Figure 4; 9.1° and 11.6° between *g*_*z*_ and each Er–O), reflecting the prolate and oblate nature of the *m*_*J*_ = |±15/2⟩ of the Er^3+^ and Dy^3+^ ions, respectively. The ground states for both complexes are extremely mixed but dominated by the *m*_*J*_ = |±15/2⟩ component (39% in **1-Er·0.5tol** and 80% in **1-Dy·0.5tol**). The first excited state for **1-Dy·0.5tol** is calculated at 47 cm^–1^ compared to 29 cm^–1^ for **1-Er·0.5tol**. These calculations are in line with observations from the experimental magnetic data above. The stabilization of high *m*_*J*_ states in low-symmetry non-ideal coordination environments is a result of the large *J* values of these ions.^57^

In contrast, **2-Ln·tol** has *D*_*3*_ symmetry with the coordination sphere being dominated by the three hard-donor N atoms in a plane and completed with six softer S atoms. Equatorial coordination in Dy^3+^ should favor low *m*_*J*_ states and easy plane anisotropy; the ground state of **2-Dy·tol** is an easy plane with the hard axis along the *C*_*3*_ axis (*g*_*z*_ = 3.0, *g*_*xy*_ ∼ 9.3, Figure 4) but it is composed of intermediate *m*_J_ states. For Er^3+^, equatorial coordination is the favored geometry to stabilize large *m*_*J*_ states and easy axis anisotropy; **2-Er·tol** is easy axis (*g*_*z*_ = 12.3, *g*_*xy*_ ∼ 0.22) and contains large *m*_J_ components (29% |±15/2⟩ + 12% |±15/2⟩) but is mixed in composition (Table 2, Figure S41). The stabilization of the intermediate *m*_J_ components in **2-Ln·tol** is attributed to the S atoms shifting the crystal field from equatorial toward isotropic, which is also reflected in the small overall CFS and low energy first excited states (Table 2). The magnetization is highly sensitive to the separation of these states, and we attribute the overpredicted magnetization at high field and low temperature for **2-Er·tol** (Figure S21) to an inaccuracy in calculating the small energy gap. Indeed, scaling all crystal field parameters to change the splitting (while approximately keeping the composition constant), the magnetization data can be well-reproduced with a first excited state at 19.1 cm^–1^ (Figure S34).

Compounds **3-Ln** have local C_1_ symmetry in both solvatomorphs but the crystal field is axial and dominated by the single short Ln–N bond, with the softer Se atoms expected to contribute less to the crystal field than the S atoms in **2-Ln**. The Dy–N bond defines the easy axis of the ground state anisotropy in **3-Dy·2thf** (Figure 4c). This ground state is highly axial (*g*_*z*_ = 19.7, *g*_*x*_, *g*_*y*_ ∼ 0.03), dominated by *m*_*J*_ = |±15/2⟩ (94%) and is well-stabilized. While all compounds except **2-Dy·tol** have highly magnetic ground states, **3-Dy·2thf** is the only compound for which there is a large energy barrier to the reversal of the magnetization (Figures S39 and S37–39). Relaxation is expected to occur via the first excited doublet at 120 cm^–1^, which has significant transverse *g*-values of 0.3–0.4. **3-Dy·dcm** is more mixed and has the first excited state at 88 cm^–1^ (Table 2). In **3-Er·dcm**, the ground state is rhombic and has a mixed composition, with *m*_*J*_ = |±15/2⟩ as the largest component (51%; 40% in **3-Er·3thf**). The hard axis is expected to lie along Er–N but is found by CASSCF-SO to be offset by 42° (Figure S41). The CASSCF-SO predicted magnetic susceptibility is also in poor agreement with experiment for **3-Er·dcm**; the ^4^I_15/2_ term spans 220 cm^–1^, comparable to **2-Er•·tol** (Table 2), as expected for Er ions in nonequatorial coordination environments. Combined, these observations led us to pursue further experimental methods of determining the electronic structure and anisotropy of **3-Er·dcm**.

### Torque Magnetometry

Cantilever torque magnetometry (CTM) measurements were performed on a single crystal of **3-Er·dcm** to experimentally determine the magnetic anisotropy. These crystals are monoclinic, with four molecules in the unit cell, two of which are magnetically unique. Measurements were conducted along three perpendicular axes (Table S26 and Figures S42–S44), at temperatures between 2 and 50 K and fields of 3–9 T. We focus initially on the 2 K data set, which reports on the ground state anisotropy as it has the largest population of the ground doublet. At 2 K, rotation 1 has a hard zero around 52° and an easy zero spaced by ∼ 90° (Figure 5), similar to what would be expected for a single magnetically unique molecule, indicating that the projections of the easy axes into the examined plane are close to aligned for the two molecules in this rotation (Figure S42). In rotations 2 and 3, the additional inflections and zero positions in the torque curve indicate summation effects between the signals of the two magnetically unique molecules.

The torque curves at 2 K, at which only the ground state is expected to be significantly populated, were fit to a model of two non-interacting effective spins of 1/2, related by crystal symmetry (Figure S45 and Table S27). The good agreement observed in the simulation of the powder magnetization curve at 2 K (Figure S30), suggests that the *g*-tensor of the ground state (*g* = 0.20, 3.36, 13.66) is correctly predicted by the *ab initio* calculations. Therefore, our fitting parameters were the mass of the crystal (too small to be measured) and the g-tensor orientation. This model was able to reproduce the main features of the torque curves (Figure 5). As there are two magnetically unique molecules this fitting gives two possible solutions (Figure S46), with the easy axis lying along a Se–Er–Se axis or between atoms. As the *m*_*J*_ = |±15/2⟩ of Er^3+^ has a prolate shape, the solution with the easy axis between atoms is more likely; this is in good agreement (5°) with the CASSCF-SO easy direction (Figure 6). The hard and intermediate directions are rotated 30° relative to the CASSCF-SO direction, with the hard axis moved closer to the Er–N vector (29° absolute angle, 7° projected in the hard plane) than in the calculation (42°, 18°). The experimentally determined orientations are in better agreement with the expected strong CF contribution of the coordinating N atom in **3-Er·dcm**.

**Figure 6:**
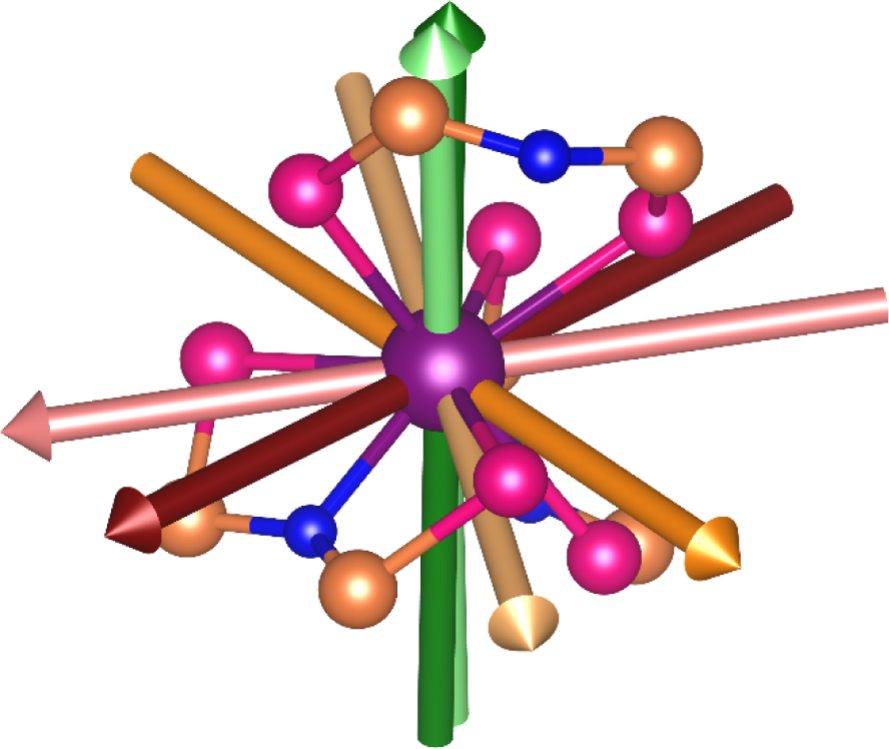
Experimentally determined ground-state anisotropy axes for **3-Er·dcm**: easy axis (dark green), intermediate axis (orange), hard axis (maroon). Overlaid CASSCF calculated orientation: easy axis (pale green), intermediate axis (pale orange), hard axis (pale maroon). Color code: Er (purple), N (blue), P (peach), Se (pink).

Simulation of the torque curves at all temperatures using the CASSCF-SO calculated crystal field parameters and the orientations determined at 2 K (Figure S47) does not provide a good reproduction of the experimental data, as the simulation includes a strong contribution from a low energy excited state that is not observed experimentally. Torque and magnetometry data taken together suggest that the first excited state lies at a higher energy (or with a different composition). This accounts for the experimental crystal field differing from the CASSCF results by more than a simple rotation and scaling of CF parameters. However, the simple *S*_eff_ = 1/2 model above confirms the ground state anisotropy of 3-Er·dcm.

The current CASSCF-SO approach remains the most tractable method for large lanthanide complexes, and it generally captures the overall electronic behavior. However, this computational pathway presents some inherent limitations, as it *i)* neglects a significant part of dynamic electron correlation, and *ii)* includes only the localized 4f orbitals in the active space, neglecting small contributions from 5d, 6s, and ligand-based orbitals. These should be included to account for covalent contributions and are expected to play a major role with softer donor atoms. However, such computational quantification of covalent contributions to lanthanide coordination bonds is still a challenging and open field of research,^58–60^ whose coverage is beyond the scope of this work.

### Dynamic Magnetic Properties

The dynamic magnetic properties of **1-Ln·0.5tol**, **2-Ln·tol** (Ln = Er, Dy), **3-Er·dcm** and **3-Dy·2thf** were investigated with alternating current (ac) magnetic susceptibility measurements on a PPMS or SQUID-XL magnetometer (Figures S48–S63). All compounds except **3-Dy·2thf** have no significant out of phase susceptibility (χ″) in zero field. For **3-Dy·2thf**, the peak in χ″ lies above the maximum accessible frequency (Figure S53). Thus, none of the compounds are zero-field SMMs due to rapid QTM in the ground doublets, reflecting the significant transverse *g*-values that are smallest for **3-Dy·2thf**.

The field-dependence at 2 K was studied for all compounds (Figures S48–S54). Except for **2-Dy·tol**, all products exhibit significant slow relaxation in an applied field; the absence of this behavior for **2-Dy·tol** is attributed to the weakly magnetic ground state and easy plane anisotropy. The field-dependent relaxation in **3-Dy·2thf** at 2 K is much slower than the other compounds and appears as a single peak. For the Er compounds and **1-Dy·0.5tol**, two features are observed at 2 K under a dc field: a narrow, fast, field-dependent peak, and a slower, broad peak around 10 Hz that grows in at fields of 1000–1500 Oe and then is field-independent above ca. 2000 Oe. The slow field-independent process, also observed as a small fraction in **2-Dy·tol**, is attributed to sample decomposition. We focus on the faster process, which is field-independent at low fields but then moves quickly at higher fields.

The temperature-dependence of the main relaxation process was studied under fixed dc fields (Figures S55–S63). The Er compounds showed peaks up to 3.5 K (**1-Er·0.5tol**, 500 and 1000 Oe) or 2.5 K (**2-Er·tol**, 1000 Oe; **3-Er·dcm**, 500 Oe) and **1-Dy·0.5tol** displayed peaks up to 4.5 K (500 and 1000 Oe). There is very little variation between the 500 and 1000 Oe data sets for **1-Ln** , consistent with the field-independence in this range. Compound **3-Dy·2thf** has peaks from 4–12 K at 500 and 1000 Oe, but 2.25–12 K at 3000 Oe (Figures S61–63). Temperature- and field-dependent relaxation rates were extracted by fitting data to the generalized Debye (one peak) or double generalized Debye (two peaks) model (Tables S28–S41).

The temperature and field-dependence of all relaxation rates were fit to a sum of Direct relaxation, phonon-pair driven Raman^7^ and/or Orbach relaxation processes (eq 1) using CC-FIT2 (Table 3, Figures 7 and S64–S68).^61,62^

1 $$\tau^{-1}\left(T,H\right)=AH^{4}T+R\frac{e^{\omega /k_{B}T}}{{\left(e^{\omega /k_{B}T}-1\right)}^{2}}+\tau_{0}^{-1}e^{-\frac{U_{\mathrm{eff}}}{k_{B}T}}$$

**Figure 7:**
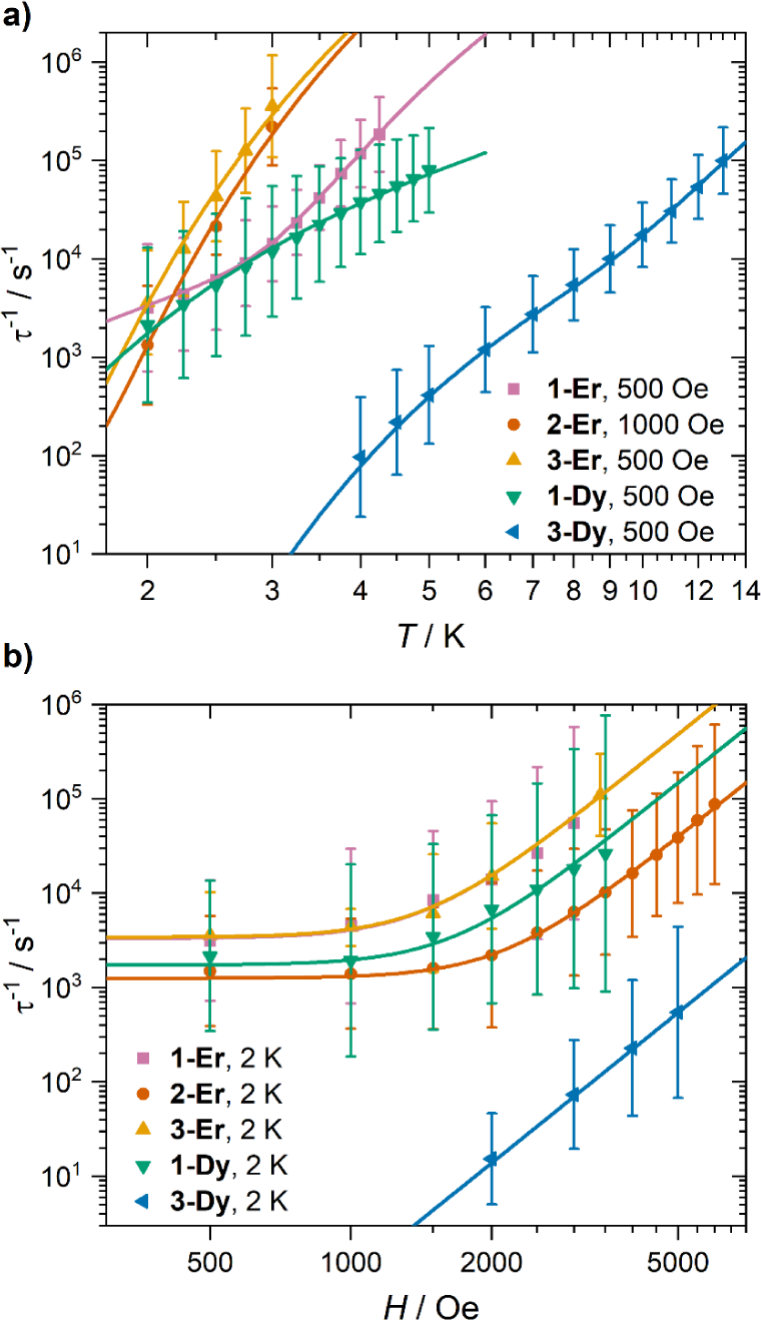
(a) Temperature-dependence of relaxation rates in **1-Ln·0.5tol**, **3-Er·dcm** and **3-Dy·2thf** at 500 Oe and **2-Er·tol** at 1000 Oe. (b) Field dependence of relaxation rates in **1-Ln·0.5tol**, **2-Er·tol**, **3-Er·dcm** and **3-Dy·2thf**. Error bars indicate 1 ESD in the distribution of rates. Solid lines indicate results of a simultaneous fit of all temperature- and field-dependent data to eq 1 with the parameters given in Table 3. The orange and pink lines in panel (b) are mostly superimposed.

**Table 3:** Fitted Magnetic Relaxation Parameters for **1-Ln**, **2-Ln**, and **3-Ln** (Ln = Dy, Er) Complexes, Including Orbach (*τ*_*0*_, *U*_*eff*_), Raman (*R*, *ω*), and Direct (*A*) Relaxation Processes as Reported in eq 1

	τ_0_^–1^/s^–1^	*U*_eff_/cm^–1^	*R*/s^–1^	ω/cm^–1^	*A*/s^–1^ Oe^–4^ K^–1^
**1-Er·0.5tol**	10^8.8(2)^	24.2(13)	10^4.3(3)^	2.9(8)	10^–9.42(4)^
**2-Er·tol**	10^9.61(12)^	20.8(5)	-	-	10^–10.51(3)^
**3-Er·dcm**	10^9.33(13)^	18.5(5)	-	-	10^–9.42(4)^
**1-Dy·0.5tol**	-	-	10^5.81(3)^	8.22(13)	10^–9.93(4)^
**3-Dy·2thf**	10^8.3(3)^	72(6)	10^5.42(8)^	22.6(7)	10^–12.36(2)^

The temperature and field dependence of the Direct process is fixed to the theoretical values for Kramer’s ions.^63^ The Raman expression derives from the two-phonon correlation function, where ω is the energy of the first Γ-point optical modes: in the limit *k*_B_ *T* ≪ *ω* the expression becomes a second Arrhenius process; in the other limits, *k*_B_ *T* ≫ *ω*, the rates are proportional to *T*.^2^ Summing up all contributions from many phonon pairs leads to the traditional power law expression *CT* ^*n*^;^7^ here we assume Raman is driven by a pair of near-equal energy phonons to extract the phonon energy.

Compound **1-Er·0.5tol** has two field-independent temperature-dependent relaxation processes (Figure S64). The process with an energy barrier of 24.2(13) cm^–1^ is assigned to an Orbach process occurring via the first excited state, which is calculated by CASSCF to be at 29.4 cm^–1^, with dc magnetic data well reproduced by these calculations. The τ_0_^–1^ value of 10^8.8(2)^ s^–1^ is also consistent with an Orbach process. These values are similar to those reported for a **1-Er** analogue with fluorinated phenyl groups, which has *U*_eff_ of 18.6 cm^–1^ and τ_0_^–1^ of 10^7.76^ s^–1^. The second relaxation process in **1-Er·0.5tol** is assigned to a Raman process with low energy 2.9(8) cm^–1^ Γ-point optical phonons, with a lower coefficient of 10^4.3(3)^ s^–1^.

In contrast to **1**-**Er·0.5tol**, compounds **2-Er·tol** and **3-Er·dcm** have a single, fast-moving thermally dependent process in low fields. Fitting of all temperature- and field-dependent data (Figures S65 and S66) indicate energy barriers of 20.8(5) and 18.5(5) cm^–1^ respectively, which are in good agreement with the energy of the first excited states: 19.1 cm^–1^ for **2-Er·tol** from fitting magnetization data and 16.0 cm^–1^ for **3-Er·dcm** from CASSCF. This suggests Orbach relaxation dominates at 2 K and low fields, consistent with large τ_0_^–1^ coefficients of 10^9.61(12)^ s^–1^ and 10^9.33(13)^ s^–1^ respectively. Compounds **1–3-Er** have similar low *U*_eff_ values confirming the absence of strong axial anisotropy; when QTM is quenched in the ground doublet by a dc field, thermally assisted QTM at the first excited state (Orbach) and Raman processes compete. For **2-Er·tol** and **3-Er·dcm**, Orbach is too fast to observe any underlying Raman process.

Compound **1-Dy·0.5tol** relaxes via a single, relatively slow-moving thermally dependent process in low fields (Figure S67). Fitting of all temperature- and field-dependent data indicates an energy barrier of 8.22(13) cm^–1^ and a coefficient of 10^5.81(3)^ s^–1^. CASSCF calculations well-reproduce magnetic data and predict a much higher first excited state at 35.8 cm^–1^, so a process with such a low barrier cannot be Orbach relaxation. We conclude that Raman is the dominant process for **1-Dy·0.5tol** in low fields; Orbach relaxation is not seen in the observable range, but could take over above 5 K. The phonons driving Raman relaxation in **1-Ln·0.5tol** are low energy (c.f. 16 cm^–1^ for [Dy­(acac)_3_(H_2_O)_2_], acac = acetylacetonate, and 12.3 cm^–1^ for a Ce^3+^ Schiff-base metal–organic framework),^7,64^ but are consistent between the two **1-Ln** complexes. Compounds **1–3-Er** and **1-Dy** have similar field-dependencies at 2 K, and above 1000 Oe, relaxation is dominated by a strongly field-dependent Direct process with coefficients of 10^–9.4^–10^–10.5^ s^–1^ Oe^4–^ K^–1^. The slowest of these compounds is **2-Er·tol**, which has a more ideal geometry for Er^3+^ ions, while the identity of coordinating atoms (**1-Er·0.5tol** vs **3-Er·dcm**) has minimal effect (Figure 7).

Compound **3-Dy·2thf** has two temperature-dependent, field-independent processes in the observed frequency range at 500 and 1000 Oe, but under 3000 Oe a field-dependent and weakly temperature-dependent Direct process switches-on and is observable within the accessible range. The relaxation rates are fit well considering Direct, phonon-pair driven Raman and an Orbach process (Figure S68). This gives values of τ_0_^–1^ = 10^8.3(3)^ s^–1^, *U*_eff_ = 72(6) cm^–1^, *R* = 10^5.42(8)^ s^–1^, ω = 22.6(7) cm^–1^ and *A* = 10^–12.36(2)^ s^–1^ Oe^–4^ K^–1^. The Orbach barrier is much smaller than the CASSCF-SO prediction of 120 cm^–1^ for **3-Dy·2thf**. However, 72(6) cm^–1^ is close to the first excited state for **3-Dy·dcm** (88 cm^–1^), indicating this value is reasonable and could be consistent with a strong sensitivity to small distortions of the structure at low temperatures. The Raman coefficient is comparable to **1-Ln·0.5tol** and the Direct coefficient is much lower than the other compounds, reflecting the higher axiality of the ground state.

It was expected that heavier chalcogen coordination (**3-Ln**) would result in lower energy vibrations compared to lighter chalcogens (**1-Ln**); contrary to expectations, a larger ω value is found for **3-Dy·2thf** than **1-Ln·0.5tol** (Table 3). The ω-value corresponds to the lowest energy optical modes at the Γ-point, and it appears that these low-energy modes do not involve the Ln–E bond, which has a bond stretch expected at a much higher energy. Instead, the complex geometry is more important for defining the low-energy phonon spectrum, and the 6-coordinate **1-Ln·0.5tol** results in very low energy phonons giving rise to rapid Raman relaxation. At the same time, the complex geometry controls the axiality of CF, which is significantly larger in **3-Dy·2thf** when directed by the short Dy–N bond, resulting in a larger *U*_eff_. The Er^3+^ complexes have low axiality and low energy first excited states, resulting in very rapid Orbach relaxation, which competes with the fast two-phonon Raman relaxation in **1-Er·0.5tol** and masks Raman relaxation in **2-Er·tol** and **3-Er·dcm**. These data show that close control of the axiality of the complex and low energy phonon spectrum via the coordination geometry and choice of Ln^III^ ion is more important in suppressing Orbach, Raman, Direct and QTM processes than vibrational effects of chalcogen substitution.

## Conclusions

In this work we have experimentally and computationally investigated the magnetic anisotropy and slow relaxation of **1–3-Ln** (Ln = Dy, Er), reported the synthesis and characterization of **2-Ln** and **3-Ln**, and achieved crystallization of **3-Tb** for the first time. The ligands are tridentate in **2-Ln** and the complexes are isostructural with the reported for early Ln, while for **3-Ln** we see a switch between a 9-coordinate complex with three κ^2^-N,Se,Se tridentate ligands for **3-Tb** to a 7-coordinate complex with one κ^3^-N,Se,Se tridentate ligand and two κ^2^-Se,Se bidentate ligands for **3-Er** and **3-Dy**. The 7-coordinate complexes have a single short Ln–N bond that directs the anisotropy of the products. Torque magnetometry confirms that in **3-Er·dcm** the easy axis lies between atoms, and the hard axis lies 7° from the projection of the Er–N vector in the hard plane. The axial crystal field imposed by the short Dy–N bond in **3-Dy·2thf** results in better field-induced SMM parameters than for the other complexes, with a thermal barrier to relaxation of 72(6) cm^–1^ and majority *m*_*J*_ = ± 15/2⟩ ground state.

The Er complexes have large angular momentum components in the ground state but are not strongly anisotropic, with low-energy excited states and in-field slow relaxation with barriers of 19–24 cm^–1^. The crystal field of **2-Ln·tol** has significant contributions from S and so is not purely equatorial, leading to underperforming slow magnetic relaxation for Er^3+^, while the imposed *D*_*3*_ symmetry ensures an easy plane ground state for **2-Dy·tol** and no slow relaxation.

In **1-Ln·0.5tol**, in-field Raman relaxation occurs with comparable fast rates and lowest Γ-point optical phonon energies of 2.9(8) and 8.22(13) cm^–1^ for **1-Er·0.5tol** and **1-Dy·0.5tol**, respectively, much less than 22.6(7) cm^–1^ for **3-Dy·2thf**. The fast Orbach processes in **2-Er·tol** and **3-Er·dcm** mask the observation of Raman relaxation in these complexes. The slowest Direct process is also observed for **3-Dy·2thf**, followed by **2-Er·tol** and then equivalent rates for O and Se donors in **1-Er·0.5tol** and **3-Er·dcm**, indicating strong dependence on the crystal field rather than vibrations. Therefore, controlling the coordination geometry, hence the crystal field, is more effective in suppressing relaxation processes than the vibrational effects of chalcogen substitution.

## Experimental Section

### General Methods

Caution! Extreme care should be taken both in the handling of the cryogen liquid nitrogen and its use in the Schlenk line trap to avoid the condensation of oxygen from air. Butyllithium is pyrophoric and must be handled using proper needle and syringe techniques.

All syntheses were performed under dinitrogen (99.999%, Praxair or Air Liquide) using Schlenk and glovebox techniques. Solvents were dried by standard methods^65^ and freshly distilled under N_2_ before use. Chemicals (98–99%) were supplied by Aldrich and used as received.

Elemental analyses were performed under argon by MEDAC Laboratories (Chobham, Surrey, UK) on a Thermal Scientific Flash ES 1112 Series instrument. Fourier-transform infrared (FTIR) spectra (4000 to 400 cm^–1^) were recorded with a Bruker Vertex 70 spectrophotometer (resolution 2 cm^–1^). Samples were analyzed from emulsions in dry mineral oil (Nujol). ^1^H (200.13 MHz), ^31^P (81.02 MHz), and ^13^C NMR (50.33 MHz) spectra were acquired at room temperature on a Bruker DPX 200 NMR spectrometer operating at 4.7 T. Chemical shifts (δ) are reported in ppm relative to tetramethylsilane as the internal reference for ^1^H and ^13^C, and H_3_PO_4_ 85% as the external reference for ^31^P.

Single-crystal X-ray diffraction data were collected on a Bruker D8 Venture diffractometer equipped with Photon 100 CMOS or Photon II-7 detectors and a graphite monochromator. Mo–Kα radiation was used for all compounds, except for **3-Dy·dcm**, for which Cu–Kα radiation was used. The crystals were covered by mineral oil, mounted on a MicroMount support (MiTeGen), and analyzed at room temperature or 100 K, depending on their moisture sensitivity. Data were processed with APEX3,^66^ APEX4,^67^ or APEX5 ^68^ software. Structures were determined by direct methods on SHELXS^69^ or SHELXT,^70^ and refined by full-matrix least-squares methods on F^2^ in SHELXL.^70^ A face absorption correction (Gaussian from crystal shape)^71^ was applied to complex **3-Er·dcm**, while a multiscan approach was employed for the other products.^66^ In the case of **3-Er·dcm**, the remaining residual density near the heavy atoms may be due to absorption effects. Conversely, the residuals away from Er and Se, and close to lighter C, N, and P atoms, indicate a likely presence of twinning that, despite attempts, could not be identified by the Platon analysis and remained unresolved in the original data.

All non-hydrogen atoms were refined with anisotropic thermal parameters, except those belonging to some of the disordered Ph rings as listed in the Supporting Information (section 2, Crystallography). All hydrogen atoms were included in idealized positions, with their *U*_iso_ values set to ride on the *U*_eq_ values of the parent carbon atoms. Scattering factors for neutral atoms were taken from the literature.^72^ The WinGX graphical interface was used to run the aforementioned software.^73^ Structure drawings were made with the Diamond,^74^ ORTEP3,^75^ and Mercury^76^ programs. The coordination geometries of the Ln^3+^ ions were confirmed from the crystallographic data using the SHAPE software.^50^ Details on the modeling of disordered lattice solvents and the use of the PLATON/SQUEEZE tool are presented in the SI.

Powder X-ray diffractograms were recorded with Bruker D8 Discovery equipment using a high-brightness Cu–Kα X-ray microsource (ca. 50 μm) with a Montel multilayer focalization system (λ = 1.5406 Å). The sample was packed in a Hilgenberg glass capillary (0.5 mm diameter, glass no. 14) and measured with a rotating capillary Debye–Scherrer vertical setup at room temperature, employing 0.01° steps from 5° to 50° in 2θ. The measurement time was about 72 h for each sample. The resulting powder pattern was analyzed with the TOPAS v.5 software (Bruker AXS Corporation).^77^

### Synthesis

Ligand and lanthanide complex precursor syntheses were adapted from literature and are given in the Supporting Information.^78^

*[Ln­{N­(OPPh*_2_)_2_}_3_ *], Ln = Er* ^*3+*^ (**1-Er**)*, Dy* ^*3+*^ (**1-Dy**). NH­(OPPh_2_)_2_ (0.719 g, 1.724 mmol) was suspended in 20 mL of thf to receive, under vigorous stirring, the dropwise addition of [Er­{N­(SiMe_3_)_2_}_3_] (0.372 g, 0.574 mmol) dissolved in 25 mL of thf. The resulting light pink solution was stirred at room temperature for 18 h and then evaporated to dryness under vacuum, yielding a light pink microcrystalline solid. This was dissolved in 25 mL of toluene and stored at −20 °C. After 2 days, 210 mg of pink crystals were isolated and dried under vacuum (product **1-Er**). The mother solution was transferred to another Schlenk tube and left undisturbed at −20 °C for 2 weeks, producing a second small batch of crystals (150 mg). Yield of **1-Er**: 0.360 g (41.5%). Elemental analysis (%, **1-Er**): calcd for [Er­{N­(OPPh_2_)_2_}_3_]·0.5C_7_H_8_ (C_72_H_60_ErN_3_O_6_P_6_·0.5C_7_H_8_): C 62.01, H 4.41, N 2.87. Found: C 61.87, H 4.59, N 3.05. Yield of **1-Dy**: 0.503 g (64.2%). Elemental analysis (%, **1-Dy**): calcd for [Dy­{N­(OPPh_2_)_2_}_3_]·0.5C_7_H_8_ (C_72_H_60_DyN_3_O_6_P_6_·0.5C_7_H_8_): C 62.21, H 4.43, N 2.88. Found: C 62.21, H 4.45, N 3.18.

*[Ln­{N­(SPPh*_2_)_2_}_3_ *], Ln = Er* ^*3+*^ (**2-Er**)*, Dy* ^*3+*^ (**2-Dy**)*.* A solution of NH­(SPPh_2_)_2_ (0.432 g, 0.962 mmol) in 14 mL of thf was transferred to a Schlenk tube and received the careful addition of [Er­{N­(SiMe_3_)_2_}_3_] (0.208 g, 0.320 mmol) dissolved in 15 mL of toluene. This created a toluene layer on top of the thf solution, which was kept at room temperature in the glovebox for liquid diffusion. After 3 days, 120 mg of light pink crystals were isolated and dried under vacuum (product **2-Er**). The mother liquor was transferred to another Schlenk tube and left undisturbed at −20 °C for 4 days, yielding an additional small batch of crystals (50 mg). **Yield of 2-Er:** 0.170 g (33.0%). Elemental analysis (%, **2-Er**): Calcd for [Er­{N­(SPPh_2_)_2_}_3_]·0.5C_7_H_8_ (C_72_H_60_ErN_3_S_6_P_6_·0.5C_7_H_8_): C 58.17, H 4.14, N 2.70. Found: C 58.28, H 4.26, N 2.85. **Yield of 2-Dy:** 0.298 g (59.6%). Elemental analysis (%, **2-Dy**): calcd for [Dy­{N­(SPPh_2_)_2_}_3_]·C_7_H_8_ (C_72_H_60_DyN_3_S_6_P_6_·C_7_H_8_): C 59.30, H 4.28, N 2.63. Found: C 59.45, H 4.32, N 2.52.

*[Ln­{N­(SePPh*_2_)_2_ *}], Ln = Er* ^*3+*^ (**3-Er**)*, Dy* ^*3+*^ (**3-Dy**). A solution of NH­(SePPh_2_)_2_ (0.453, 0.834 mmol) in 3 mL of thf was carefully layered with a solution of [Er­{N­(SiMe_3_)_2_}_3_] (0.181 g, 0.278 mmol) in 5 mL of toluene. The resulting mixture was kept at room temperature in the glovebox for liquid diffusion. After 2 days, light pink crystals were isolated by filtration (product **3-Er**). **Yield of 3-Er:** 0.493 g (87.3%). Elemental analysis (%, **3-Er**): calcd for [Er­{N­(SePPh_2_)_2_}_3_]·C_4_H_8_O (C_72_H_60_ErN_3_Se_6_P_6_·C_4_H_8_O): C 48.91, H 3.67, N 2.25. Found: C 48.88, H 4.14, N 2.26. **Yield of 3-Dy:** 0.511 g (63.1%). Elemental analysis (%, **3-Dy**): calcd for [Dy­{N­(SePPh_2_)_2_}_3_]·2C_4_H_8_O (C_72_H_60_DyN_3_Se_6_P_6_·2C_4_H_8_O): C 49.69, H 3.96, N 2.17. Found: C 49.56, H 4.08, N 2.27.

### Magnetism

Magnetic measurements were performed on microcrystalline powder samples wrapped in Teflon tape and pressed into a pellet. The pellet was suspended inside a plastic straw using cotton thread and the straw fixed to the end of the sample rod. The S- and Se-based pellets were prepared inside a glovebox. dc measurements were performed on a Quantum Design SQUID-XL7 and ac measurements on this instrument or a Quantum Design PPMS. The χ*T* diamagnetic correction was obtained by comparing measurements performed at 1 and 10 kOe at room temperature, or when this did not provide sensible results, by subtracting the contribution of the Teflon (−3.7 × 10^–7^ cm^3^ g^–1^). Data were corrected for the diamagnetic contribution of the sample, calculated as the molecular weight (g mol^–1^) multiplied by −0.5 × 10^–6^ cm^3^ mol^–1^.

Ac data with one peak were fit to a Generalized Debye model:

$$\tau^{-1}=\chi_{S}+\frac{\Delta \chi }{1+{\left(i\omega \tau \right)}^{1-\alpha }}$$

Where *χ*_*s*_ is the adiabatic susceptibility, Δ_*χ*_ is equal to *χ*_*T*_–*χ*_*S*_ where *χ*_*T*_ is the isothermal susceptibility, *ω* is the angular frequency, *τ* is the characteristic relaxation time and α reflects the distribution of relaxation times. Ac data with two peaks were fit to a Double Generalized Debye model:

$$\tau^{-1}=\chi_{S}+\frac{\Delta \chi_{1}}{1+{\left(i\omega \tau_{1}\right)}^{1-\alpha_{1}}}+\frac{\Delta \chi_{2}}{1+{\left(i\omega \tau_{2}\right)}^{1-\alpha_{2}}}$$

where *χ*_S_ = *χ*_S1_+ *χ*_S2_ (assuming *χ*_S1_ = *χ*_S2_), Δ*χ*_1_ = *χ*_T1_ –*χ*_S1_ and Δ*χ*_2_ = *χ*_T2_ – *χ*_S2_. Fitting was performed in a customized MATLAB script. For **3-Er·dcm**, the 2 K in-field data was not fit well using the Double Generalized Debye equation above, and so was fit to a rearranged equation:

$$\tau^{-1}=\chi_{S}+\frac{\chi_{T}-\chi_{\mathrm{mid}}}{1+{\left(i\omega \tau_{2}\right)}^{1-\alpha_{2}}}+\frac{\chi_{\mathrm{mid}}-\chi_{S}}{1+{\left(i\omega \tau_{1}\right)}^{1-\alpha_{1}}}$$

where *χ*_S_ = *χ*_S1_ + *χ*_S2_, *χ*_T_ = *χ*_T1_ + *χ*_T2_, and *χ*_mid_ = *χ*_S2_+*χ*_T1_. This fitting was done in a different customized MATLAB script, employing fminuit and weighting the out-of-phase data to the in-phase data 2:1. The temperature and field-dependence of relaxation rates was fit using a customized python script, using the ccfit2 package.^61,62^

### CASSCF Calculations

The calculations were based on the X-ray structural data, with hydrogen positions further optimized via density functional theory (DFT) using the ORCA 5.0.4 software.^79^ The ωB97X functional was employed,^80^ combined with the D3 dispersion correction^81^ and the Douglas–Kroll–Hess (DKH) Hamiltonian for scalar relativistic effects. For atoms directly bonded to the lanthanide (nitrogen and oxygen), the DKH-def-TZVP basis set^82^ was utilized, while SARC2-DKH-QZVP^83^ was applied to the lanthanide atoms. All other atoms (selenium, sulfur, carbon, hydrogen, and phosphorus) were treated with the DKH-def-SVP basis set. This basis set scheme was consistently applied in post-Hartree–Fock (post-HF) calculations of electronic properties, which were conducted at the complete active space self-consistent field (CASSCF) level of theory. The active space comprised nine electrons in the 4f orbitals of the Dy atom, denoted as CAS­(9,7), and 11 electrons in the 4f orbitals of the Er atom, denoted as CAS­(11,7). State-average calculations were performed to include all 21 sextuplets, 224 quartets, and 490 doublets of the Dy ground ^6^H term, as well as all 35 quartets and 112 doublets of the Er ground ^4^I term, followed by spin–orbit perturbation. The magnitudes and orientations of the magnetic anisotropy axes for the ground and excited Kramers doublets were determined using the SINGLE_ANISO module and the pseudospin *S^eff^* = 1/2 approach.^84^

### Torque Magnetometry

Measurements were performed on a single crystal of **3-Er·dcm** on a Quantum Design PPMS using the torque insert. The crystal was secured on an acetate plate with Apiezon N-grease and indexed crystallographically using an Oxford Diffraction Xcalibur 3CCD four-circle diffractometer with a graphite monochromator and Mo-Ka radiation. The crystal maximum dimensions were 0.128 × 0.245 × 0.313, and the mass was estimated as 3.95 μg. In the fitting procedure the mass was revised to 1.96(3) μg, which may indicate partial decomposition of the air sensitive crystal. Measurements were performed for three perpendicular orientations of the crystal (Figures S41–S43), rotating between θ = 0–200° in 5° increments (+θ corresponds to sample rotating clockwise). For rotation 3, a Teflon cube was used to support the acetate lying perpendicular to the cantilever surface. Torque measurements were performed at temperatures of 2, 5, 10, 20, and 50 K and fields of 3, 5, 7, and 9 T, depending on the intensity of the signal. The temperature was unstable at 10 K for rotation 1 (8.0–13.0 K) and 50 K for rotation 2 (47.1–50.2 K). The torque data were background-corrected by point-by-point subtracting the measured value for an empty piece of acetate (rotations 1 and 2) or the acetate supported by the Teflon cube (rotation 3) under the same field and temperature conditions. There was a remaining vertical offset in the data set, due to the intrinsic magnetoresistance of the instrument, which was corrected by subtracting the average position where the different field traces crossed, treating each temperature and rotation separately. The error in aligning the crystal by eye is around 5°. The position of the hard zero in simulations of rotation 1 was insensitive to small changes in the *g*-tensor orientations, so the rotation 1 data set was offset by - 6° to provide better agreement. Torque simulations were performed in a customized script using EasySpin version 6.0.6;^85^ further details are provided in the ESI.

## Supplementary Material



## Notes

Crystallographic data; infrared spectra; powder X-ray diffraction patterns; CASSCF-SO calculations; dc and ac magnetic measurements; cantilever torque magnetometry measurements. Data available from authors upon reasonable request (PDF).
