# Supplementary material for: Balance between Crystal Field and Phonon Optimization in Defining the Magnetic Properties of Lanthanide Dichalcogenoimidodiphosphinate Complexes
Source: Inorg Chem. 2025 Oct 27;64(44):21879–92. doi: 10.1021/acs.inorgchem.5c03025 (PMC12606704; doi:10.1021/acs.inorgchem.5c03025)
Supplement: Supplementary file 1 [file ic5c03025_si_001.pdf]

Supplementary Material

Balance between crystal field and phonon optimization in defining the magnetic properties of lanthanide dichalcogenoimidodiphosphinate complexes

José Severiano Carneiro Neto[a,b], Gemma K. Gransbury[a], Lucas G. Fachini[b], Francielli S. Santana[b], Diego Seckler[b], Arsen Raza[c], Patrick Severin Sfragano[a], Damiano Tanini[a], Antonella Capperucci[a], Matteo Briganti[d], Jaísa F. Soares[b]*, Mauro Perfetti[d]**

a. *Department of Chemistry "Ugo Schiff", University of Florence, Via della Lastruccia 3-13, 50019 Sesto Fiorentino (FI), Italy.*
b. *Departamento de Química, Universidade Federal do Paraná, Centro Politécnico, Jardim das Américas, 81530-900 Curitiba-PR, Brazil.*
c. *Department of Industrial Engineering, DIEF and INSTM Research Unit, University of Florence, 50139 Florence, Italy.*
d. *Department of Chemistry "Ugo Schiff", University of Florence and INSTM Research Unit, Via della Lastruccia 3-13, 50019 Sesto Fiorentino (FI), Italy.*

* *matteo.briganti@unifi.it; jaisa.soares@ufpr.br; mauro.perfetti@unifi.it*

Table of Contents

1. Synthesis 3

2. Crystallography 6

3. Infrared Spectroscopy 31

4. DC Magnetometry 34

5. CASSCF-SO Calculations 42

6. Cantilever Torque Magnetometry 57

7. AC Magnetometry 62

8. References 96

1. Synthesis

Synthesis and purification of the $HN(EPPh_2)_2$ ligands (*E = O, S, Se*)

Synthesis of $HN(OPPh_2)_2$

The procedure was adapted from the literature.[1] A colorless solution of chlorodiphenylphosphine (1.103 g, 5.00 mmol) in 10 mL of freshly distilled toluene was added dropwise to a toluene solution (10 mL) of hexamethyldisilazane (0.4035 g, 2.50 mmol). After addition, the mixture was heated at 80-90 ºC for 5 h to distill off chlorotrimethylsilane (boiling point 57 ºC). The system was then cooled down to room temperature, and H_2O_2 35% (w/w, 0.52 mL, 6.0 mmol) dissolved in 6 mL of tetrahydrofuran was added slowly. After stirring for four hours, the reaction mixture was filtered to give 0.170 g of a white microcrystalline solid that was dried under vacuum. The filtrate was left at room temperature overnight to produce a second batch of product (0.630 g). This solid (0.800 g) was dissolved in dichloromethane (25 mL), and the resulting solution was filtered through Celite and silica (60 Å, 230–400 mesh). After concentrating the solution and cooling it down overnight at -20 ºC, 540 mg of colorless crystals were filtered and dried under vacuum.

Yield of $HN(OPPh_2)_2$: 0.800 g (76.7%). Recrystallization yield: 0.540 g (67.5%). ^{31}P NMR (81.02 MHz, $CDCl_3$): δ = 20.9 ppm. Elemental analysis (%) calcd for $C_{24}H_{21}NO_2P_2$: C 69.1, H 5.07, N 3.36; found: C 69.1, H 5.16, N 3.18.

Synthesis of $HN(SPPh_2)_2$

Due to the similar procedures for obtaining the imidotetraphenyldichalcogenophosphinate ligands with S and Se as donor atoms, only the representative synthetic route for $HN(SPPh_2)_2$ is described here. Yields for the synthesis and recrystallization steps are provided for both products.

Chlorodiphenylphosphine (2.21 g, 1.80 mL, 10.0 mmol) in 10 mL of toluene was added dropwise to 20.0 mL of a toluene solution of hexamethyldisilazane (hmds, 808 mg, 1.05 mL, 5.00 mmol). The

reaction mixture developed a greenish hue with slight turbidity during the addition, returning to colorless after a few minutes. The mixture was then heated to distill trimethylsilyl chloride (Scheme S1, top) and cooled to room temperature to receive the addition of elemental sulfur (400 mg, 12.5 mmol) suspended in 10 mL of toluene. This reaction system was then heated under reflux at 130 ºC for *ca* 14 h (Scheme S1, bottom).

After reflux, the mother solution was cooled to -20 ºC for 24 h, yielding 1.69 g of a white microcrystalline solid, which was isolated by filtration and dried under vacuum. The solid product was washed with toluene and carbon disulfide to remove residual sulfur. Recrystallization was performed by dissolving the product in 35 mL of CH_2Cl_2, followed by filtration through Celite and silica (60 Å, 230-400 mesh), concentration under vacuum, and cooling to -20 ºC for two days. This gave colorless crystals that were filtered and dried under vacuum.

Yield of $HN(SPPh_2)_2$: 1.46 g (64.9%). Recrystallization yield: 0.80 g (54.7%)

Yield of $HN(SePPh_2)_2$: 1.42 g (52.2%). Recrystallization yield: 0.70 g (49.2%).

^{31}P NMR (81.02 MHz, $CDCl_3$): δ = 57.5 ppm for $HN(SPPh_2)_2$ and 53.1 ppm for $HN(SePPh_2)_2$. Elemental analysis (%) calcd for $C_{24}H_{21}NSe_2P_2$: C 53.1, H 3.90, N 2.58; found: C 53.9, H 4.26, N 2.55.

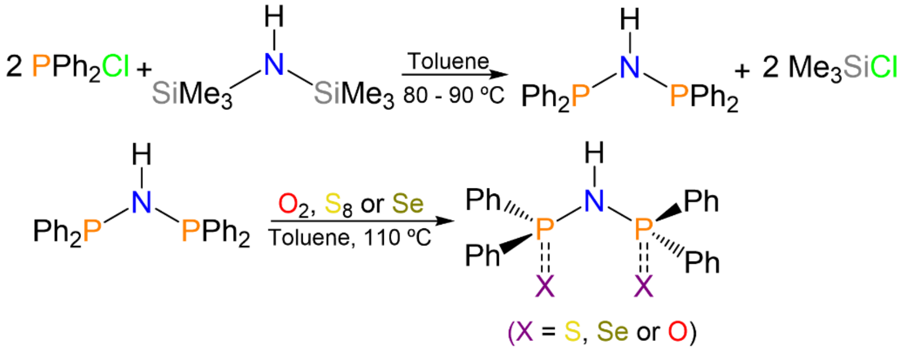

Scheme S1. Synthesis of the $HN(XPPh_2)_2$ proligands.

Preparation of the lanthanoid(III) chloride precursors

Preparation of trans-*[$LnCl_4(thf)_2$]*trans-*[$LnCl_2(thf)_5$] (Ln = Er, Dy)*

The lanthanoid precursors were prepared by dehydration of $LnCl_3{\cdot}6H_2O$ with triethylorthoformate in thf.[2] The procedure for obtaining the ion pair complex [$DyCl_3(thf)_5$][$DyCl_4(thf)_2$] will be described below; the same methodology was applied to the other Ln cations.

$DyCl_3{\cdot}6H_2O$ (1.03 g, 2.73 mmol) and 5.5 mL (33.0 mmol) of triethyl orthoformate (teof, $C_7H_{16}O_3$) were mixed in 40 mL of thf. The reaction mixture was stirred vigorously for 40 min, quickly turning into a colorless solution. Subsequently, the solvent was completely removed under vacuum with gentle heating (30-40 ºC), yielding a white microcrystalline solid. This procedure (addition of thf + teof and drying) was repeated a second time. After the final drying step, the resulting white solid was dissolved in 30 mL of thf, and the solution was filtered through Celite and cooled overnight to -20 ºC. This gave 0.90 g of a white microcrystalline solid that was isolated by filtration and gently dried under a dinitrogen stream to minimize solvent loss. After concentrating the mother solution and cooling it to -20 ºC, a second batch of product (200 mg) was obtained.

Yield of [$LnCl_2(thf)_5$][$LnCl_4(thf)_2$]: 1.10 g (77.3 %) and 0.62 g (72.8 %) for Ln = Dy^{3+} and Er^{3+}, respectively.

Synthesis of [Ln{$N(SiMe_3)_2$}$_3$] (Ln = Er, Dy)

The representative synthesis of [Dy{$N(SiMe_3)_2$}$_3$] is described in detail, and yields are given for both the Er and Dy products. [Li{$N(SiMe_3)_2$}] (0.912 g, 5.44 mmol) was dissolved in 15 mL of thf, transferred to a sublimation flask, and cooled down to 0 ºC. [$DyCl_2(thf)_5$][$DyCl_4(thf)_2$] (0.901 g, 0.906 mmol) was then suspended in 15 mL of thf and added slowly to the lithium amide solution to give a colorless mixture, which was stirred for approximately 14 h at room temperature and then evaporated to dryness under vacuum. The resulting solid was sublimated between 160 and 180 ºC (oil bath temperature)

at 10^{-3} Torr to produce a batch of colorless crystals that dissolved almost completely in 20 mL of pentane. Following filtration through Celite, the solution was concentrated under vacuum and cooled to –20 ºC. A few hours later, colorless needles were isolated by solvent evaporation to dryness under vacuum.

Yield of [Dy{N(SiMe$_3$)$_2$}$_3$]: 0.570 g (49.1%). Recrystallization yield: 0.18 g (31.6%).

Yield of [Er{N(SiMe$_3$)$_2$}$_3$]: 1.05 g (71.9%). Recrystallization yield: 0.53 g (50.3%).

2. Crystallography

Additional refinement details

Solvent disorder modeling and use of the PLATON SQUEEZE routine

The toluene molecule reported for **1-Er·0.5tol** is disordered over a crystallographic inversion center, so the C73–C79 occupancy was fixed at 0.5, and the ring and methyl carbon atoms were refined with anisotropic thermal parameters. In **1-Dy·0.5tol**, whose data were collected at room temperature (298 K), the lattice toluene was even more disordered, leading to unsuccessful attempts at atomistic modeling. Therefore, the PLATON SQUEEZE routine[3] was used to account for this solvent contribution to the calculated structure factors.

PLATON SQUEEZE was also used to model highly disordered solvent electron density in **2-Dy·tol**, **2-Er·tol**, **3-Er·3thf**, **3-Dy·2thf**, and **3-Tb·tol**. In **3-Er·dcm** and **3-Dy·dcm**, on the other hand, the crystallizing solvent was well defined, without disorder, and was successfully modeled in the difference maps.

In **2-Er·tol**, the likely solvent-related (remaining) 294 electrons were distributed in the 1532 Å^3 vacant volume, making up 14.0% of the unit cell. Then, using 50 electrons per toluene molecule, 294/50 = 5.88; 5.88/6 (since Z = 6) gave 0.98 solvent molecule per **2-Er** unit. In **2-Dy·tol**, the remaining electrons and voids were 276 and 1496 Å^3, respectively, corresponding to 13.8% of the unit cell and 0.92 molecule of toluene per unit of the complex. The complex **3-Tb·tol**, which is similar to the **2-Ln·tol** products,

contains a nine-coordinate lanthanide cation but possesses N and Se as donor atoms instead of N and S. It also presents one extensively disordered toluene molecule, whose electron density was treated with PLATON SQUEEZE.

In the case of **3-Er·3thf**, one thf molecule was located and refined anisotropically. The remaining 356 electrons were spread in 671 Å^3 solvent-accessible volume, corresponding to 8.4% of the unit cell. Considering 40 electrons per thf molecule, 356/40 = 8.90; 8.90/4 (as Z = 4) = 2.22 thf molecules per unit of the complex, which were processed with PLATON SQUEEZE. Analogously, for **3-Dy·2thf**, after modeling and refining anisotropically one of the lattice thf molecules (O1, C73-C76), the remaining 184 electrons occupied the solvent-accessible volume of 648 Å^3 (8.4% of the unit cell). These 184 electrons corresponded to 1.15 thf molecules per unit of the complex.

In all cases where PLATON SQUEEZE was employed, a mixed approach was adopted where the better-defined part of the total electron density, including the non-disordered solvent and the disordered phenyl rings in the ligands (see below), was modeled and refined anisotropically or isotropically in the usual manner. Afterwards, the electron density corresponding to the severely disordered solvent molecules was back-Fourier transformed to render their contribution to the calculated structure factors as described by Spek.[4, 5]

Disorder modeling – Phenyl rings in the ligands

In **1-Er·0.5tol**, two disordered phenyl groups (C13-C18 and C55-C60) were modeled in two parts using FLAT restraints. The two conformations of C55-C60 were restrained with SAME, ensuring the components of C55 shared the same position and anisotropic displacement parameters. The atoms of C14-C18 (parts A and B) were modeled isotropically, while C13 and all atoms in both parts of the C55-C60 ring were refined anisotropically.

For **1-Dy·0.5tol**, non-hydrogen atoms were refined with anisotropic thermal parameters, except for the minor component (part B) of the disordered C13–C18 and C55–C60 rings, where geometric restraints

(DFIX and FLAT) were applied. Treating the likely disorder in the other rings, indicated by elongated ellipsoids, was hampered by issues arising after refinement cycles, causing the atoms to "jump" out of the ring structure, even with constraints. The fact that data for **1-Dy·0.5tol** were collected at 298 K likely contributed to these difficulties in the anisotropic refinement.

In **2-Ln·0.5tol**, **3-Ln·dcm** (Ln = Er and Dy), and **3-Tb·tol**, all non-hydrogen atoms were refined with anisotropic thermal parameters, and no atom in the ligand presented disorder to be treated.

In **3-Er·3thf**, three phenyl groups (C7-C12, C19-C24, and C37-C42) were disordered and modeled with two components (A and B) using FLAT, SIMU, and DFIX restraints, as well as ADP constraints. Despite several attempts, refining these components anisotropically was not possible, likely due to lower data quality from collection at 300 K instead of a low temperature. These difficulties were similar to those described above for data on **1-Dy·0.5tol**, also collected at room temperature. Therefore, all the disordered atoms were treated isotropically.

In **3-Dy·2thf**, one disordered phenyl group (C7-C12) was modeled with two components using FLAT and DFIX restraints. The major component (part A) was refined anisotropically, while the atoms of part B were refined isotropically.

Table S1. Crystal and refinement data for **1-Ln·0.5tol**. Radiation wavelength: 0.71073 Å (Mo-Kα)

**Complex**	**1-Er·0.5tol**	**1-Dy·0.5tol**
Elemental formula	$2(C_{72}H_{60}ErN_3O_6P_6)$, C_7H_8	$C_{72}H_{60}DyN_3O_6P_6$, $0.5(C_7H_8)$
CCDC deposition number	2456286	2456287
Formula weight (g mol^{-1})	2924.76	1457.61
Crystal system / Space group	Triclinic, *P*-1	Triclinic, *P*-1
Temperature (K)	100(2)	298(2)
Unit cell axes (Å)	a = 13.1341(4)	a = 13.434(2)
	b = 14.0116(5)	b = 14.191(2)
	c = 21.4570(8)	c = 21.665(4)
Unit cell angles (º)	α = 71.107(2)	α = 71.395(4)
	β = 82.8060(10)	β = 82.719(4)
	γ = 62.9760(10)	γ = 62.729(4)°
Cell volume (Å^3) / Z / F(000)	3327.0(2) / 1 / 1488	3478.5(10) / 2 / 1484
Crystal color, shape	light pink prism	colorless prism
Density, calculated (Mg m^{-3})	1.460	1.392
Absorption coefficient (mm^{-1})	1.462	1.266
θ range for data collection (º)	2.0 to 32.6	2.2 to 25.0
hkl range	$-19 \leq h \leq 19$, $-21 \leq k \leq 21$, $-32 \leq l \leq 32$	$-15 \leq h \leq 15$, $-16 \leq k \leq 16$, $-25 \leq l \leq 25$
Reflections collected / unique	227052 / 24211 [R(int) = 0.056]	57443 / 12187 [R(int) = 0.089]
No. of 'observed' reflections ($I > 2\sigma_I$)	22330	9035
Absorption correction / Refinement	Semi-empirical from equivalents / Full-matrix least-squares on F^2	
Transmission (max / min)	0.7465 and 0.6407	0.7455 and 0.6954
Data / restrictions / parameters	24211 / 12 / 898	12187 / 27 / 835
Goodness-of-fit (F^2)	1.027	1.067
Final R indexes [I>2σ(I)] *	R_1 = 0.032, wR_2 = 0.074	R_1 = 0.043, wR_2 = 0.100
Final R indexes (all data) *	R_1 = 0.036, wR_2 = 0.076	R_1 = 0.075, wR_2 = 0.115
Largest diff. peak and hole (e Å^{-3})	1.68 and -1.69	0.95 and -0.91
Location of the largest diff. peak	1.06 Å from Er1	1.63 Å from O2

* **1-Er·0.5tol:** $w = [\sigma^2(Fo^2)+(0.0200P)^2+8.5507P]^{-1}$, with $P=(Fo^2+2Fc^2)/3$; **1-Dy·0.5tol:** $w = [\sigma^2(Fo^2)+(0.0601{*}P)^2]^{-1}$, with $P=(Fo^2+2Fc^2)/3$.

Table S2. Selected bond lengths (Å) and angles (°) for **1-Er·0.5tol** and **1-Dy·0.5tol**

**Bond length**	**1-Er·0.5tol**	**1-Dy·0.5tol**	**Bond angle**	**1-Er·0.5tol**	**1-Dy·0.5tol**
Ln–O1	2.2573(14)	2.295(3)	O2–Ln–O1	83.40(5)	82.44(12)
Ln–O2	2.2660(14)	2.293(4)	O4–Ln–O3	82.54(5)	81.34(11)
Ln–O3	2.2406(14)	2.266(3)	O5–Ln–O6	82.76(5)	81.67(12)
Ln–O4	2.2292(13)	2.265(3)	O6–Ln–O1	86.06(6)	85.46(11)
Ln–O5	2.2174(14)	2.245(3)	O4–Ln–O1	93.17(5)	93.24(11)
Ln–O6	2.2347(14)	2.265(3)	O5–Ln–O4	99.63(5)	101.69(11)
N1–P1	1.5896(17)	1.592(4)	O5–Ln–O1	165.51(5)	163.24(11)
N1–P2	1.5934(17)	1.581(4)	O3–Ln–O2	172.42(5)	171.73(12)
N2–P3	1.5905(17)	1.583(4)	O4–Ln–O6	168.29(5)	167.02(12)
N2–P4	1.5916(17)	1.589(4)	O3–Ln–O1	90.46(5)	91.12(12)
N3–P5	1.5912(18)	1.589(4)	O5–Ln–O3	97.84(5)	98.56(13)
N3–P6	1.5916(18)	1.593(4)	O6–Ln–O3	85.78(5)	85.78(12)
O1–P1	1.5199(14)	1.513(3)	O4–Ln–O2	93.32(5)	93.82(12)
O2–P2	1.5249(15)	1.520(4)	O5–Ln–O2	89.09(5)	88.96(12)
O3–P3	1.5223(15)	1.518(3)	O6–Ln–O2	98.19(5)	98.80(13)
O4–P4	1.5265(14)	1.525(3)	P2–N1–P1	124.18(11)	125.6(3)
O5–P5	1.5240(14)	1.522(3)	P3–N2–P4	127.37(11)	128.4(2)
O6–P6	1.5180(14)	1.514(3)	P5–N3–P6	127.93(11)	128.0(3)

Table S3. Continuous shape measures[6] of the coordination polyhedra for **1-Er** and **1-Dy** compared to literature data. The symmetries in parentheses are for the ideal polyhedral shapes

Geometry	1-Er·0.5tol	1-Dy·0.5tol	1-Er·0.5H$_2$O ($R\bar{3}$)[16]		1-Dy ($P\bar{3}$)[17]		
			**Er1***	**Er2***	**Dy1***	**Dy2***	**Dy3***
Hexagon (D_{6h})	32.711	32.842	33.474	34.256	34.377	34.253	33.897
Pentagonal pyramid (C_{5v})	23.669	22.824	25.659	16.848	20.071	20.798	24.090
**Octahedron (O_h)**	**0.941**	**1.206**	**0.801**	15.448	4.624	**3.754**	**1.390**
**Trigonal prism (D_{3h})**	11.883	11.570	11.383	**0.064**	**4.238**	5.210	9.350
Johnson pentagonal pyramid J2 (C_{5v})	27.250	26.421	29.460	20.937	24.076	24.772	27.928

* The Er1, Er2, Dy1, Dy2, Dy3 labels for **1-Er·0.5H$_2$O** ($R\bar{3}$) and **1-Dy** ($P\bar{3}$) refer to the crystallographically independent Ln centers in the asymmetric units.

Table S4. Crystal and refinement data for **2-Ln·tol**. Radiation wavelength: 0.71073 Å (Mo-Kα)

Complex	2-Er·tol	2-Dy·tol
Elemental formula	$C_{72}H_{60}ErN_3P_6S_6$, C_7H_8	$C_{72}H_{60}DyN_3P_6S_6$, C_7H_8
CCDC deposition number	2456288	2456289
Formula weight (g mol^{-1})	1604.80	1600.04
Crystal system / Space group	Trigonal, *R*-3c	Trigonal, *R*-3c
Temperature (K)	100(2)	100(2)
Unit cell axes (Å)	a = 14.958(2)	a = 14.9430(9)
	b = 14.958(2)	b = 14.9430(9)
	c = 56.304(12)	c = 56.151(5)
Unit cell angles (°)	α = β = 90°, γ = 120°	α = β = 90°, γ = 120°
Cell volume (Å^3) / Z / F(000)	10910(4) / 6 / 4902	10858.4(16) / 6 / 4890
Crystal color, shape	light-pink rhomb	colorless rhomb
Density, calculated (Mg m^{-3})	1.466	1.468
Absorption coefficient (mm^{-1})	1.505	1.385
θ range for data collection (°)	2.7 to 27.5	2.7 to 29.6
hkl range	$-19\leq h\leq 19$	$-20\leq h\leq 20$
	$-19\leq k\leq 19$	$-20\leq k\leq 20$
	$-73\leq l\leq 73$	$-77\leq l\leq 78$
Reflections collected / unique	191578 / 2797 [R(int) = 0.051]	219651 / 3405 [R(int) = 0.033]
No. of 'observed' reflections (I > 2σ_I)	2634	3203
Absorption correction / Refinement	Semi-empirical from equivalents / Full-matrix least-squares on F^2	
Transmission (max / min)	0.7260 and 0.6757	0.7459 and 0.6994
Data / restrictions / parameters	2797 / 0 / 134	3405 / 0 / 134
Goodness-of-fit (F^2)	1.128	1.118
Final R indexes [I>2σ(I)] *	R_1 = 0.018, wR_2 = 0.043	R_1 = 0.018, wR_2 = 0.043
Final R indexes (all data) *	R_1 = 0.021, wR_2 = 0.044	R_1 = 0.021, wR_2 = 0.044
Largest diff. peak and hole (e Å^{-3})	0.48 and -0.34	0.58 and -0.38
Location of the largest diff. peak	1.42 Å from S1	1.42 Å from S1

* **2-Er·tol**: w = $[\sigma^2(Fo^2)+(0.0167P)^2+30.5235P]^{-1}$, where $P=(Fo^2+2Fc^2)/3$; **2-Dy·tol:** w = $[\sigma^2(Fo^2)+(0.0164P)^2+28.1724P]^{-1}$ where $P=(Fo^2+2Fc^2)/3$.

Table S5. Selected bond lengths (Å) and angles (°) for **2-Er·tol** and **2-Dy·tol**

Bond length	2-Er·tol	2-Dy·tol	Bond angle	2-Er·tol	Bond angle	2-Dy·tol
Ln–S1	2.9121(5)	2.9236(3)	N1^{ii}–Er–N1	119.999(1)	N1–Dy–N1^{ii}	119.999(1)
Ln–S1^{i}	2.9122(5)	2.9236(3)	S1^{iii}–Er–S1^{i}	152.297(15)	S1^{iii}–Dy–S1^{ii}	152.540(12)
Ln–S1^{ii}	2.9122(5)	2.9236(3)	S1^{iv}–Er–S1^{ii}	152.298(15)	S1^{iv}–Dy–S1^{i}	152.542(12)
Ln–S1^{iii}	2.9120(5)	2.9235(3)	S1–Er–S1^{v}	152.295(15)	S1^{v}–Dy–S1	152.539(12)
Ln–S1^{iv}	2.9121(5)	2.9235(3)	N1–Er–S1^{i}	133.927(10)	N1–Dy–S1	134.004(7)
Ln–S1^{v}	2.9121(5)	2.9236(3)	N1^{i}–Er–S1^{ii}	133.931(10)	N1–Dy–S1^{iv}	134.006(7)
Ln–N1	2.5421(17)	2.5545(14)	N1^{i}–Er–S1^{iii}	133.925(10)	N1^{i}–Dy–S1^{i}	134.008(7)
Ln–N1^{i}	2.5420(17)	2.5545(14)	N1–Er–S1^{v}	133.930(10)	N1^{i}–Dy–S1^{iii}	134.002(7)
Ln–N1^{ii}	2.5421(17)	2.5546(14)	N1^{ii}–Er–S1	133.931(10)	N1^{ii}–Dy–S1^{ii}	134.008(7)
P1–S1	1.9847(6)	1.9831(4)	N1^{ii}–Er–S1^{iv}	133.926(10)	N1^{ii}–Dy–S1^{v}	134.003(7)
N1–P1	1.6126(6)	-----	S1^{iii}–Er–S1	125.955(16)	S1^{iii}–Dy–S1	125.565(12)
N1–P1^{iii}	1.6124(6)	-----	S1^{iv}–Er–S1^{i}	125.953(16)	S1^{iv}–Dy–S1^{ii}	125.562(12)
N1–P^{i}	-----	1.6111(5)	S1^{v}–Er–S1^{ii}	125.954(16)	S1^{v}–Dy–S1^{i}	125.564(12)
N1–P^{v}	-----	1.6111(5)	S1^{iv}–Er–S1	92.14(2)	S1^{iv}–Dy–S1	91.990(14)
			S1^{v}–Er–S1^{i}	92.14(2)	S1^{v}–Dy–S1^{ii}	91.989(14)
			S1^{iii}–Er–S1^{ii}	92.14(2)	S1^{iii}–Dy–S1^{i}	91.990(14)
			N1–Er–S1^{ii}	76.149(7)	N1–Dy–S1^{ii}	76.270(6)
			N1–Er–S1^{iv}	76.149(7)	N1–Dy–S1^{iii}	76.270(6)
			N1^{i}–Er–S1	76.150(7)	N1^{i}–Dy–S1	76.273(6)
			N1^{i}–Er–S1^{v}	76.145(8)	N1^{i}–Dy–S1^{v}	76.267(6)
			N1^{ii}–Er–S1^{i}	76.152(8)	N1^{ii}–Dy–S1^{i}	76.272(6)
			N1^{ii}–Er–S1^{iii}	76.144(8)	N1^{ii}–Dy–S1^{iv}	76.269(6)
			S1–Er–S1^{i}	75.228(16)	S1–Dy–S1^{i}	75.412(11)
			S1–Er–S1^{ii}	75.229(16)	S1–Dy–S1^{ii}	75.413(11)
			S1^{i}–Er–S1^{ii}	75.228(16)	S1^{ii}–Dy–S1^{i}	75.413(11)
			S1^{iii}–Er–S1^{iv}	75.231(16)	S1^{iii}–Dy–S1^{iv}	75.416(11)
			S1^{iii}–Er–S1^{v}	75.231(16)	S1^{iii}–Dy–S1^{v}	75.416(11)
			S1^{iv}–Er–S1^{v}	75.230(16)	S1^{iv}–Dy–S1^{v}	75.415(11)
			N1–Er–S1	62.977(8)	N1–Dy–S1^{i}	62.782(6)
			N1–Er–S1^{iii}	62.977(8)	N1–Dy–S1^{v}	62.782(6)
			N1^{i}–Er–S1^{i}	62.981(8)	N1^{i}–Dy–S1^{ii}	62.786(6)
			N1^{i}–Er–S1^{ii}	62.972(8)	N1^{i}–Dy–S1^{iv}	62.777(6)
			N1^{ii}–Er–S1^{ii}	62.979(8)	N1^{ii}–Dy–S1	62.786(6)
			N1^{ii}–Er–S1^{v}	62.975(8)	N1^{ii}–Dy–S1^{iii}	62.779(6)
			P1^{iii}–N1–P1	147.07(12)	P^{v}–N1–P^{i}	147.35(10)
			P1–N1–Er	106.46(6)	P^{i}–N1–Dy	106.33(5)
			P1–S1–Er	84.873(18)	P–S1–Dy	84.828(14)

2-Er: Symmetry transformations used to generate equivalent atoms:
i –x+y+1,–x+1,z
ii –y+1,x–y,z
iii –x+4/3,–x+y+2/3,–z+1/6
iv y+1/3,x–1/3,–z+1/6
v x–y+1/3,–y+2/3,–z+1/6

2-Dy: Symmetry transformations used to generate equivalent atoms:

i –x+y+1,–x+1,z
ii –y+1,x–y,z
iii x–y+1/3,–y+2/3,–z+1/6
iv y+1/3,x–1/3,–z+1/6
v –x+4/3,–x+y+2/3,–z+1/6

Table S6. Crystal and refinement data for **3-Er·dcm** and **3-Dy·dcm**. λ = 0.71073 Å (Mo-Kα) for **3-Er·dcm** and λ = 1.54178 Å (Cu-Kα) for **3-Dy·dcm**

Complex	3-Er·dcm	3-Dy·dcm
Elemental formula	$C_{72}H_{60}ErN_3P_6Se_6$, CH_2Cl_2	$C_{72}H_{60}DyN_3P_6Se_6$, CH_2Cl_2
CCDC deposition number	2456290	2456291
Formula weight (g mol^{-1})	1878.99	1874.23
Crystal system / Space group	Monoclinic, $P2_1$/n	Monoclinic, $P2_1$/n
Temperature (K)	100(2)	102(2)
Unit cell axes (Å)	a = 13.3237(10)	a = 13.3462(4)
	b = 38.434(2)	b = 38.5054(12)
	c = 14.0429(10)	c = 14.0422(4)
Unit cell angles (º)	β = 94.129(3)	β = 94.1660(10)°
Cell volume (Å^3) / Z / F(000)	7172.4(8) / 4 / 3668	7197.2(4) / 4 / 3660
Crystal color, shape	colorless prism	colorless prism
Density, calculated (Mg m^{-3})	1.740	1.730
Absorption coefficient (mm^{-1})	4.4500	11.314
θ range for data collection (º)	2.4 to 26.0	2.3 to 80.8
hkl range	$-16\leq h\leq 16$, $-47\leq k\leq 47$, $-17\leq l\leq 17$	$-16\leq h\leq 12$, $-49\leq k\leq 48$, $-17\leq l\leq 17$
Reflections collected / unique	432781 / 14090 [R(int) = 0.154]	182173 / 15693 [R(int) = 0.039]
No. of 'observed' reflections (I > $2\sigma_I$)	9559	15199
Absorption correction / Refinement	Gaussian from crystal shape / Full-matrix least-squares on F^2	Semi-empirical from equivalents / Full-matrix least-squares on F^2
Transmission (max / min)	0.7503 and 0.4675	0.7543 and 0.5239
Data / restrictions / parameters	14090 / 0 / 820	15693 / 0 / 820
Goodness-of-fit (F^2)	1.267	1.027
Final R indexes [I>2σ(I)] *	R_1 = 0.062, wR_2 = 0.145	R_1 = 0.024, wR_2 = 0.059
Final R indexes (all data) *	R_1 = 0.129, wR_2 = 0.189	R_1 = 0.026, wR_2 = 0.060
Largest diff. peak and hole (e Å^{-3})	3.84 and -1.79	1.06 and -0.76
Location of the largest diff. peak	0.02 Å from Er1	0.91 Å from Cl2

* **3-Er·CH_2Cl_2**: $w = [\sigma^2(Fo^2)+(0.07689)^2+72.1212P]^{-1}$ where $P=(Fo^2+2Fc^2)/3$;
3-Dy·CH_2Cl_2: $w = [\sigma^2(Fo^2)+(0.0241P)^2+15.7510P]^{-1}$ where $P=(Fo^2+2Fc^2)/3$.

Table S7. Selected bond lengths (Å) and angles (°) for **3-Er·dcm** and **3-Dy·dcm**

**Bond length**	**3-Er·dcm**	**3-Dy·dcm**	**Bond angle**	**3-Er·dcm**	**3-Dy·dcm**
Ln-N(1)	2.398(7)	2.4264(18)	Se(1)-Ln(1)-Se(2)	132.36(3)	131.859(8)
Ln–Se(1)	2.8858(10)	2.9055(3)	N(1)-Ln(1)-Se(1)	67.91(16)	67.79(4)
Ln–Se(2)	2.9260(10)	2.9512(3)	N(1)-Ln(1)-Se(2)	67.92(16)	67.59(4)
Ln–Se(3)	2.8618(9)	2.8878(3)	Se(3)-Ln(1)-Se(4)	88.87(3)	88.598(8)
Ln–Se(4)	2.8935(10)	2.9081(3)	Se(5)-Ln(1)-Se(6)	87.29(3)	87.119(8)
Ln–Se(5)	2.8896(9)	2.9092(3)	N(1)-Ln(1)-Se(3)	94.14(17)	94.26(4)
Ln–Se(6)	2.8923(10)	2.9174(3)	N(1)-Ln(1)-Se(6)	84.96(17)	84.85(4)
Ln-P(1)	3.284(2)	3.2993(6)	N(1)-Ln(1)-Se(4)	153.21(16)	153.45(4)
Ln-P(2)	3.296(2)	3.3098(5)	N(1)-Ln(1)-Se(5)	136.40(16)	136.22(4)
N(1)-P(1)	1.624(7)	1.6276(18)	Se(1)-Ln(1)-Se(4)	138.24(3)	138.083(9)
N(1)-P(2)	1.633(7)	1.6295(18)	Se(1)-Ln(1)-Se(5)	76.46(3)	76.627(8)
N(2)-P(3)	1.576(7)	1.5909(19)	Se(1)-Ln(1)-Se(6)	113.86(3)	113.923(8)
N(2)-P(4)	1.590(7)	1.5958(19)	Se(3)-Ln(1)-Se(1)	76.47(3)	76.391(7)
N(3)-P(5)	1.618(8)	1.608(2)	Se(3)-Ln(1)-Se(2)	89.80(3)	89.800(7)
N(3)-P(6)	1.573(8)	1.598(2)	Se(3)-Ln(1)-Se(5)	101.30(3)	101.498(8)
P(1)-Se(1)	2.135(2)	2.1387(6)	Se(3)-Ln(1)-Se(6)	168.13(3)	168.117(8)
P(2)-Se(2)	2.148(2)	2.1492(6)	Se(4)-Ln(1)-Se(2)	85.51(3)	86.056(8)
P(3)-Se(3)	2.178(2)	2.1792(6)	Se(5)-Ln(1)-Se(2)	151.11(3)	151.414(9)
P(4)-Se(4)	2.160(2)	2.1672(6)	Se(5)-Ln(1)-Se(4)	68.33(3)	68.280(8)
P(5)-Se(5)	2.170(2)	2.1663(6)	Se(6)-Ln(1)-Se(2)	78.88(3)	78.888(7)
P(6)-Se(6)	2.168(2)	2.1646(6)	Se(6)-Ln(1)-Se(4)	86.72(3)	87.021(8)
			P(1)-Ln(1)-P(2)	55.49(6)	55.329(13)

Table S8. Crystal and refinement data for **3-Er·3thf** and **3-Dy·2thf**. Wavelength: 0.71073 Å (Mo-Kα)

**Complex**	**3-Er·3thf**	**3-Dy·2thf**
Elemental formula	$C_{72}H_{60}ErN_3P_6Se_6$, 3(C_4H_8O)	$C_{72}H_{60}DyN_3P_6Se_6$, 2(C_4H_8O)
CCDC deposition number	2456292	2456293
Formula weight (g mol^{-1})	2010.38	1933.51
Crystal system / Space group	Monoclinic, $P2_1$/n	Monoclinic, $P2_1$/n
Temperature (K)	300(2)	100(2)
Unit cell axes (Å)	a = 21.6010(14)	a = 21.3878(16)
	b = 14.1355(9)	b = 13.9645(9)
	c = 26.2929(16)	c = 25.841(2)
Unit cell angles (°)	β = 91.395(2)	β = 91.426(3)
Cell volume (Å^3) / Z / F(000)	8025.9(9) / 4 / 3804	7715.6(10) / 4 / 3812
Crystal color, shape	light pink prism	colorless prism
Density, calculated (Mg m^{-3})	1.601	1.665
Absorption coefficient (mm^{-1})	3.933	3.972
θ range for data collection (°)	2.3 to 27.0	2.2 to 27.9
hkl range	$-27\leq h\leq 27$, $-18\leq k\leq 18$, $-33\leq l\leq 25$	$-28\leq h\leq 28$, $-18\leq k\leq 18$, $-33\leq l\leq 33$
Reflections collected / unique	142609 / 17495 [R(int) = 0.114]	345667 / 18394 [R(int) = 0.084]
No. of 'observed' reflections ($I > 2\sigma_I$)	11028	14722
Absorption correction / Refinement	Semi-empirical from equivalents / Full-matrix least-squares on F^2	
Transmission (max / min)	0.7455 and 0.4864	0.7458 and 0.5009
Data / restrictions / parameters	17495 / 15 / 747	18394 / 9 / 851
Goodness-of-fit (F^2)	1.065	1.106
Final R indexes [I>2σ(I)] *	R_1 = 0.066, wR_2 = 0.147	R_1 = 0.038, wR_2 = 0.072
Final R indexes (all data) *	R_1 = 0.121, wR_2 = 0.169	R_1 = 0.059, wR_2 = 0.078
Largest diff. peak and hole (e Å^{-3})	1.89 and -1.30	1.52 and -1.27
Location of the largest diff. peak	0.80 Å from Er1	1.28 Å from Dy1

* **3-Er·thf**: $w = [\sigma^2(Fo^2)+(0.0674P)^2+36.7421P]^{-1}$, where $P=(Fo^2+2Fc^2)/3$; **3-Dy·thf**: $w = [\sigma^2(Fo^2)+(0.0125P)^2+40.1989P]^{-1}$, where $P=(Fo^2+2Fc^2)/3$.

Table S9. Selected bond lengths (Å) and angles (°) for **3-Er·3thf** and **3-Dy·2thf**

**Bond length**	**3-Er·3thf**	**3-Dy·2thf**	**Bond angle**	**3-Er·3thf**	**3-Dy·2thf**
Ln-N(1)	2.412(6)	2.399(3)	Se(1)-Ln(1)-Se(2)	133.50(3)	132.750(12)
Ln–Se(1)	2.9295(9)	2.9574(4)	N(1)-Ln(1)-Se(1)	67.15(14)	66.47(7)
Ln–Se(2)	2.9327(10)	2.9621(4)	N(1)-Ln(1)-Se(2)	66.64(14)	66.46(7)
Ln–Se(3)	2.8726(8)	2.9049(4)	Se(3)-Ln(1)-Se(4)	87.53(2)	86.771(13)
Ln–Se(4)	2.9260(9)	2.9344(5)	Se(5)-Ln(1)-Se(6)	86.60(3)	87.169(12)
Ln–Se(5)	2.9297(10)	2.9385(4)	N(1)-Ln(1)-Se(3)	90.62(14)	92.13(7)
Ln–Se(6)	2.8833(8)	2.8863(4)	N(1)-Ln(1)-Se(6)	91.35(14)	90.28(7)
Ln-P(1)	3.321(2)	3.3379(11)	N(1)-Ln(1)-Se(4)	140.81(14)	149.68(7)
Ln-P(2)	3.330(2)	3.3313(10)	N(1)-Ln(1)-Se(5)	149.88(14)	141.57(7)
N(1)-P(1)	1.635(6)	1.631(3)	Se(1)-Ln(1)-Se(4)	148.65(3)	142.365(12)
N(1)-P(2)	1.622(7)	1.629(3)	Se(1)-Ln(1)-Se(5)	83.35(3)	77.784(12)
N(2)-P(3)	1.593(6)	1.599(3)	Se(1)-Ln(1)-Se(6)	97.84(3)	107.362(13)
N(2)-P(4)	1.596(6)	1.591(3)	Se(3)-Ln(1)-Se(1)	76.37(3)	79.726(12)
N(3)-P(5)	1.582(7)	1.599(3)	Se(3)-Ln(1)-Se(2)	108.94(3)	98.118(12)
N(3)-P(6)	1.602(7)	1.599(3)	Se(3)-Ln(1)-Se(5)	88.13(3)	95.059(12)
P(1)-Se(1)	2.139(2)	2.1428(10)	Se(3)-Ln(1)-Se(6)	172.60(3)	172.888(13)
P(2)-Se(2)	2.142(2)	2.1369(11)	Se(4)-Ln(1)-Se(2)	77.04(3)	83.677(12)
P(3)-Se(3)	2.160(2)	2.1775(10)	Se(5)-Ln(1)-Se(2)	141.43(3)	148.457(13)
P(4)-Se(4)	2.161(2)	2.1663(10)	Se(5)-Ln(1)-Se(4)	69.22(3)	68.561(11)
P(5)-Se(5)	2.166(2)	2.1665(10)	Se(6)-Ln(1)-Se(2)	78.37(3)	76.739(11)
P(6)-Se(6)	2.180(2)	2.1618(10)	Se(6)-Ln(1)-Se(4)	95.43(3)	87.767(14)
			P(1)-Ln(1)-P(2)	55.02(5)	54.55(2)

Table S10. Crystal and refinement data for **3-Tb·tol**. Radiation wavelength: 0.71073 Å (Mo-Kα)

**Complex**	**3-Tb·tol**
Elemental formula	$C_{72}H_{60}N_3P_6Se_6Tb$, C_7H_8
CCDC deposition number	2456294
Formula weight (g mol^{-1})	1877.86
Crystal system / Space group	Trigonal, *R*-3c
Temperature (K)	100(2)
Unit cell axes (Å)	a = 15.1064(10)
	b = 15.1064(10)
	c = 56.730(6)
Unit cell angles (°)	α = β = 90°, γ = 120°
Cell volume (Å^3) / Z / F(000)	11211.6(19) / 6 / 5532
Crystal color, shape	colorless block
Density, calculated (Mg m^{-3})	1.669
Absorption coefficient (mm^{-1})	4.042
θ range for data collection (°)	3.9 to 27.5
hkl range	-19≤*h*≤19
	-19≤*k*≤19
	-73≤*l*≤73
Reflections collected / unique	117299 / 2865 [R(int) = 0.053]
No. of 'observed' reflections (I > 2σ_I)	2589
Absorption correction / Refinement	Semi-empirical from equivalents /
	Full-matrix least-squares on F^2
Transmission (max / min)	0.7457 and 0.7043
Data / restrictions / parameters	2865 / 0 / 134
Goodness-of-fit (F^2)	1.051
Final R indexes [I>2σ(I)] *	R_1 = 0.014, wR_2 = 0.033
Final R indexes (all data) *	R_1 = 0.019, wR_2 = 0.034
Largest diff. peak and hole (e Å^{-3})	0.39 and -0.35
Location of the largest diff. peak	0.73 Å from C7

* $w=[\sigma_2(F_o^2)+(0.0152P)^2+22.8728P]^{-1}$, where $P=(F_o^2+2F_c^2)/3$

Table S11. Selected bond lengths (Å) and angles (°) for **3-Tb·tol**

**Bond length**	**3-Tb·tol**	**Bond angle**	**3-Tb·tol**
Tb–Se1	3.0224(2	N1^{i}–Tb–N1	119.999(1)
Tb–Se1^{i}	3.0224(2	Se1^{iii}–Tb–Se1^{i}	152.131(6)
Tb–Se1^{ii}	3.0225(2)	Se1^{iv}–Tb–Se1^{ii}	152.133(6)
Tb–Se1^{iii}	3.0223(2)	Se1–Tb–Se1^{v}	152.129(6)
Tb–Se1^{iv}	3.0224(2)	N1–Tb–Se1^{i}	133.528(4)
Tb–Se1^{v}	3.0224(2)	N1^{i}–Tb–Se1^{ii}	133.533(4)
Tb–N1	2.6304(16)	N1^{i}–Tb–Se1^{iii}	133.526(6)
Tb–N1^{i}	2.6304(16)	N1–Tb–Se1^{v}	133.533(4)
Tb–N1^{ii}	2.6304(16	N1^{ii}–Tb–Se1	133.533(4)
P1–Se1	2.1347(4)	N1^{ii}–Tb–Se1^{iv}	133.527(6)
N1–P1	1.6131(6)	Se1^{iii}–Tb–Se1	126.780(6)
N1–P1^{iii}	1.6130(6)	Se1^{iv}–Tb–Se1^{i}	126.777(6)
		Se1^{v}–Tb–Se1^{ii}	126.780(6)
		Se1^{iv}–Tb–Se1	92.940(9)
		Se1^{v}–Tb–Se1^{i}	92.939(8)
		Se1^{iii}–Tb–Se1^{ii}	92.941(8)
		N1–Tb–Se1^{ii}	76.065(3)
		N1–Tb–Se1^{iv}	76.068(3)
		N1^{i}–Tb–Se1	76.068(3)
		N1^{i}–Tb–Se1^{v}	76.062(3)
		N1^{ii}–Tb–Se1^{i}	76.069(3)
		N1^{ii}–Tb–Se1^{iii}	76.062(3)
		Se1–Tb–Se1^{i}	74.512(6)
		Se1–Tb–Se1^{ii}	74.512(6)
		Se1^{i}–Tb–Se1^{ii}	74.512(6)
		Se1^{iii}–Tb–Se1^{iv}	74.515(8)
		Se1^{iii}–Tb–Se1^{v}	74.515(8)
		Se1^{iv}–Tb–Se1^{v}	74.513(7)
		N1–Tb–Se1	63.389(3)
		N1–Tb–Se1^{iii}	63.390(3)
		N1^{i}–Tb–Se1^{i}	63.394(3)
		N1^{i}–Tb–Se1^{iv}	63.383(3)
		N1^{ii}–Tb–Se1^{ii}	63.392(3)
		N1^{ii}–Tb–Se1^{v}	63.388(3)
		P1^{iii}–N1–P1	144.01(11)
		P1–N1–Tb	107.99(6)
		P1–Se1–Tb	83.103(11)

Symmetry transformations used to generate equivalent atoms:
i -x+y+1,-x+1,z *ii* -y+1,x-y,z
iii -x+4/3,-x+y+2/3,-z+7/6 *iv* y+1/3,x-1/3,-z+7/6 *v* x-y+1/3,-y+2/3,-z+7/6

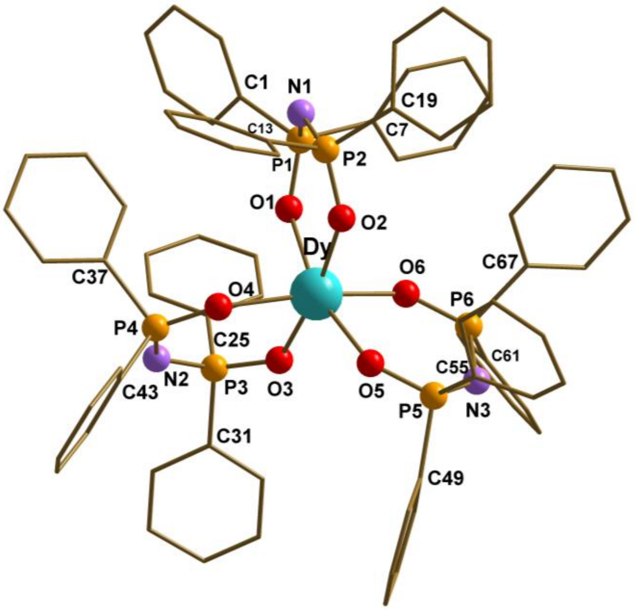

Figure S1. Representation of the molecular structure of **1-Dy·0.5tol**, $[Dy\{N(\underline{O}PPh_2)_2\}_3]$·0.5tol, with atom numbering in the chelate rings. The hydrogen atoms, the second disordered orientation of the C55–C60 phenyl ring, and the disordered toluene solvate were omitted for clarity.

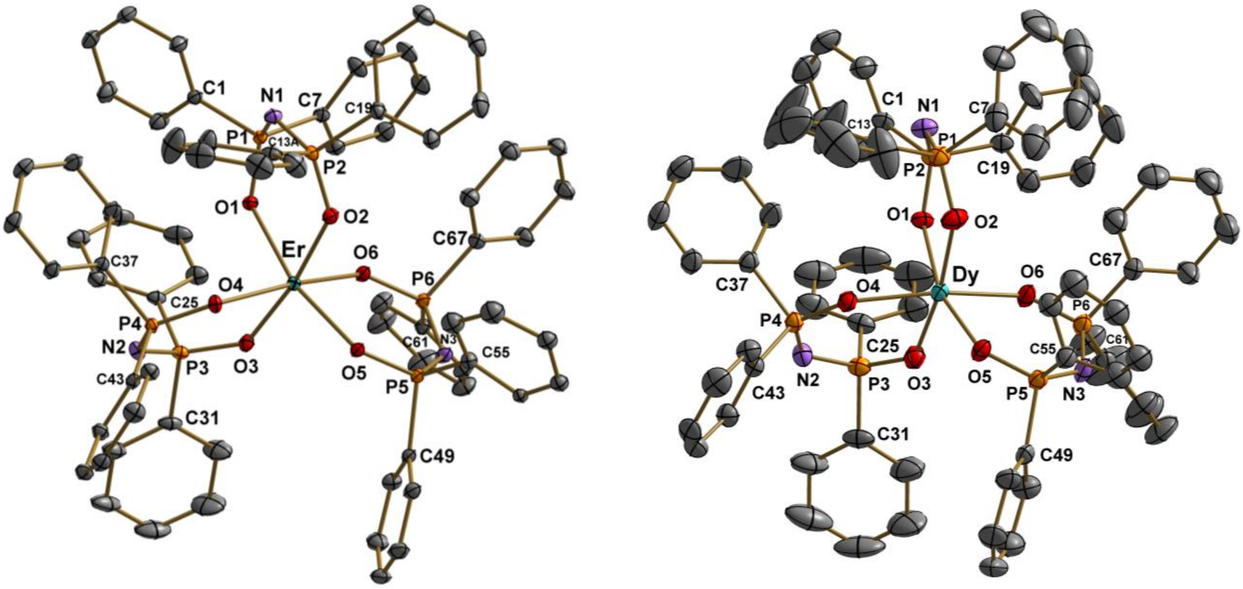

Figure S2. Ellipsoid plots for **1-Er·0.5tol** and **1-Dy·0.5tol**, drawn at the 50% and 40% probability level, respectively.

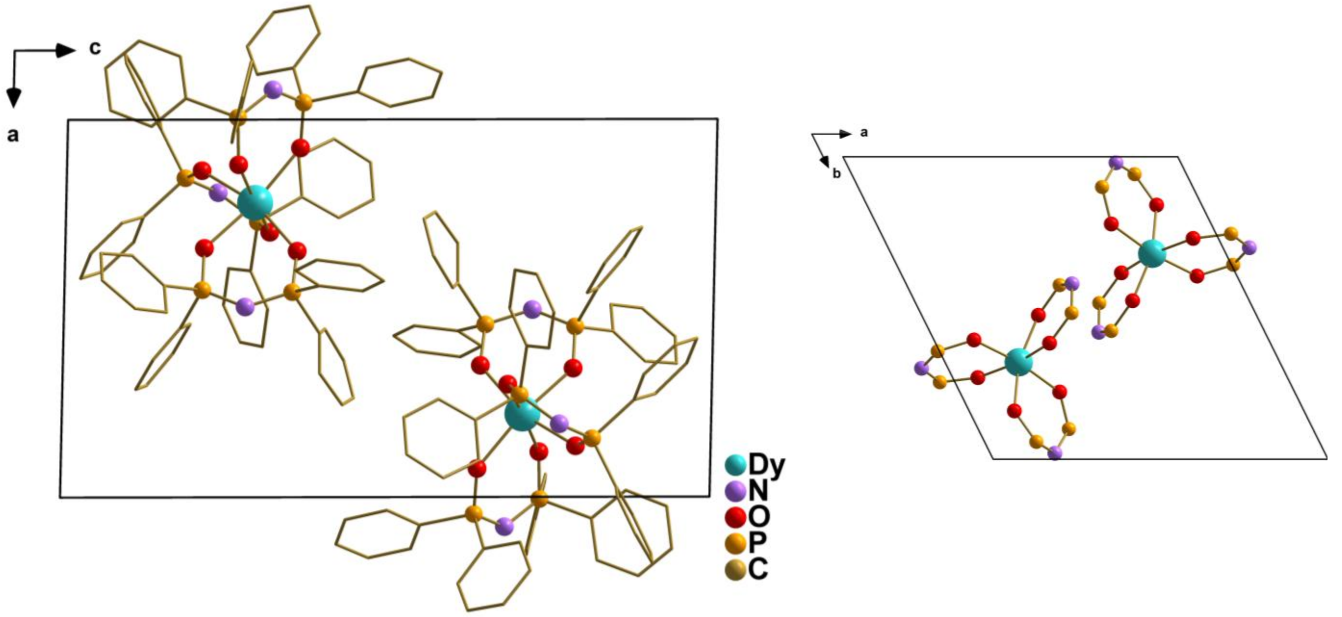

Figure S3. Left: Ball-and-stick representation of the unit cell of product **1-Dy·0.5tol** down the *b* axis, excluding hydrogen atoms and disordered solvent (toluene). Right: **1-Dy·0.5tol** molecules along the *c* axis. Only the atoms forming the chelate rings are displayed to illustrate the core structure of **1-Dy·0.5tol**. Corresponding diagrams for **1-Er·0.5tol** show no significant differences at this representation level.

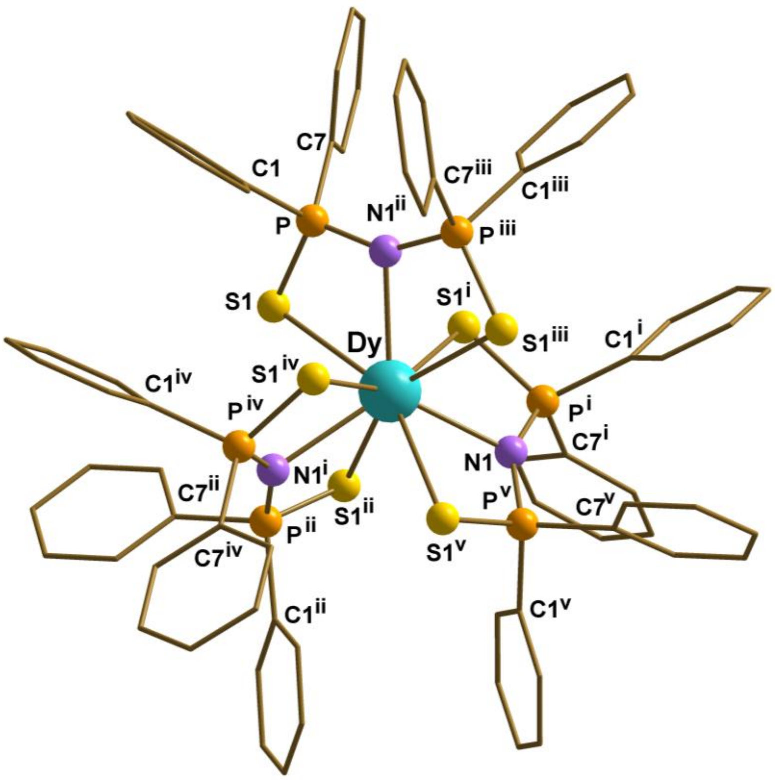

Figure S4. Ball-and-stick representation of the molecular structure of product **2-Dy·tol**, [Dy{N($SPPh_2$)$_2$}$_3$]·tol, with the atom numbering scheme. Hydrogen atoms and the disordered toluene solvate were omitted for clarity.

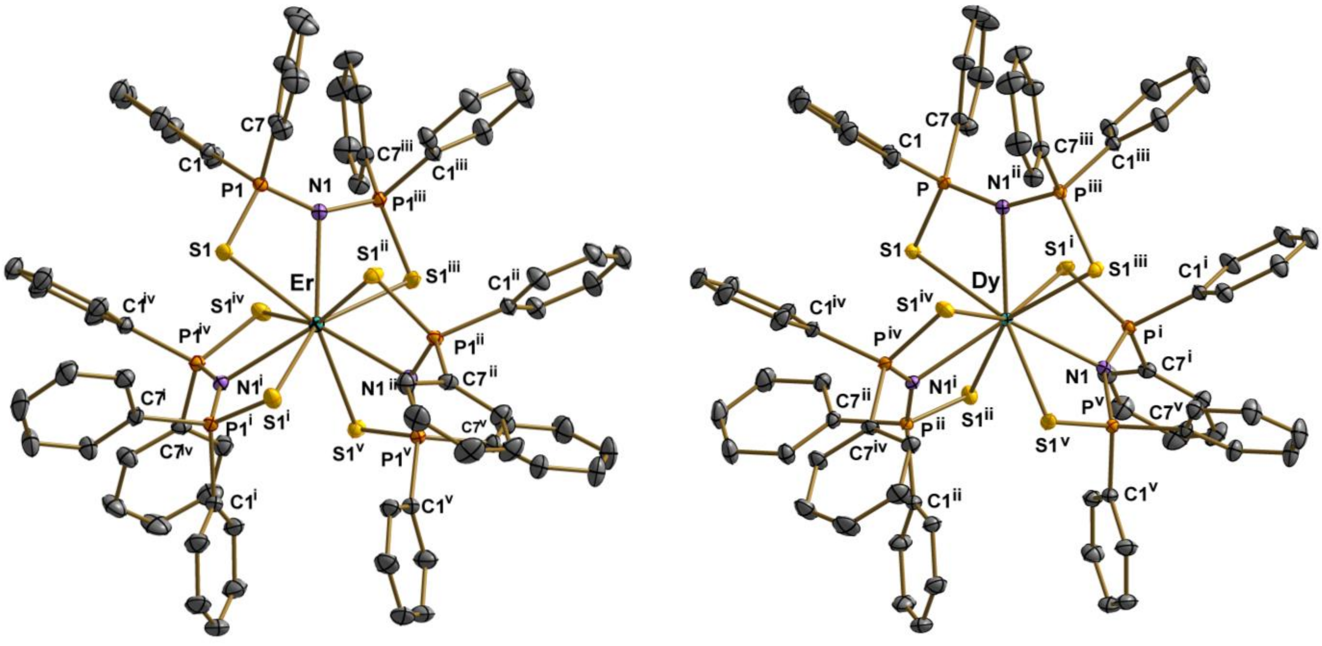

Figure S5. Ellipsoid plots for **2-Er·tol** and **2-Dy·tol**, drawn at the 50% probability level.

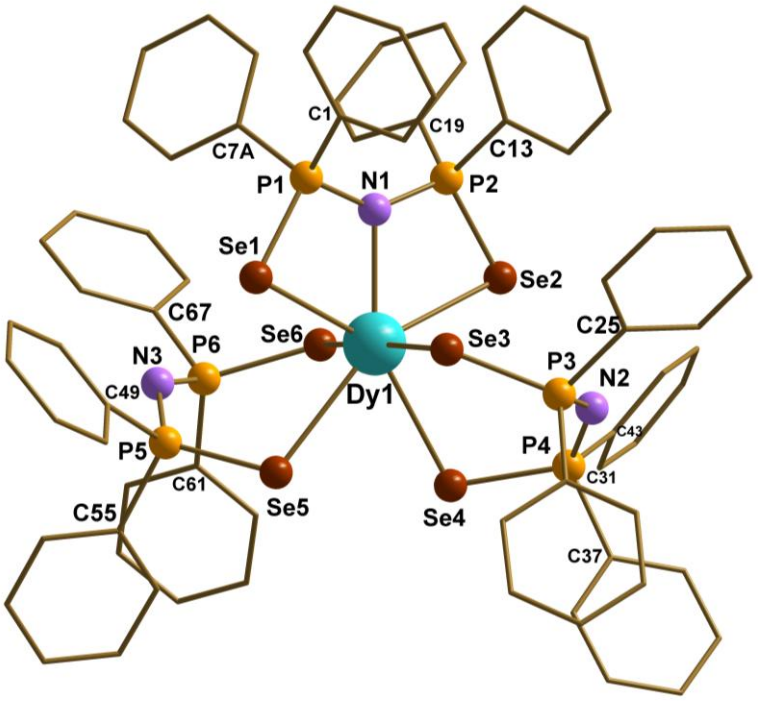

Figure S6. Representation of the molecular structure of product **3-Dy·2thf**, $[Dy\{N(SePPh_2)_2\}_3]\cdot 2thf$, with the atom numbering scheme. The hydrogen atoms, the second disordered orientation of the C7-C12 phenyl ring, and the thf solvate molecules were omitted for clarity.

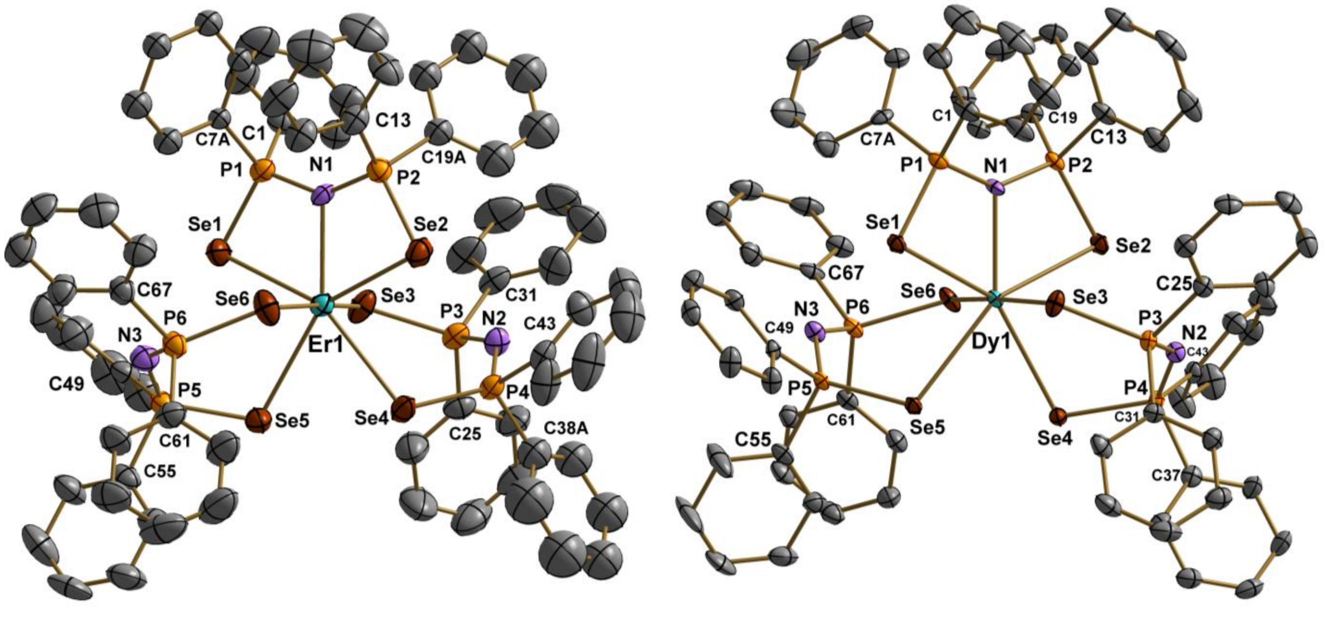

Figure S7. Ellipsoid plots for isostructural **3-Er·3thf** and **3-Dy·2thf**, drawn respectively at the 40% and 50% probability levels.

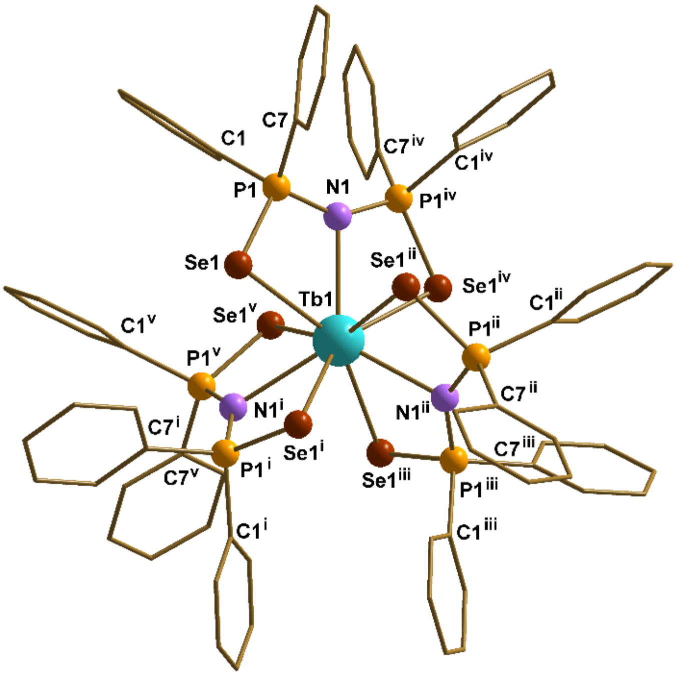

Figure S8. Ball-and-stick representation of the molecular structure of product **3-Tb·tol**, [Tb{N(SePPh$_2$)$_2$}$_3$]·tol, with the atom numbering scheme. Hydrogen atoms and the disordered toluene solvate were omitted for clarity.

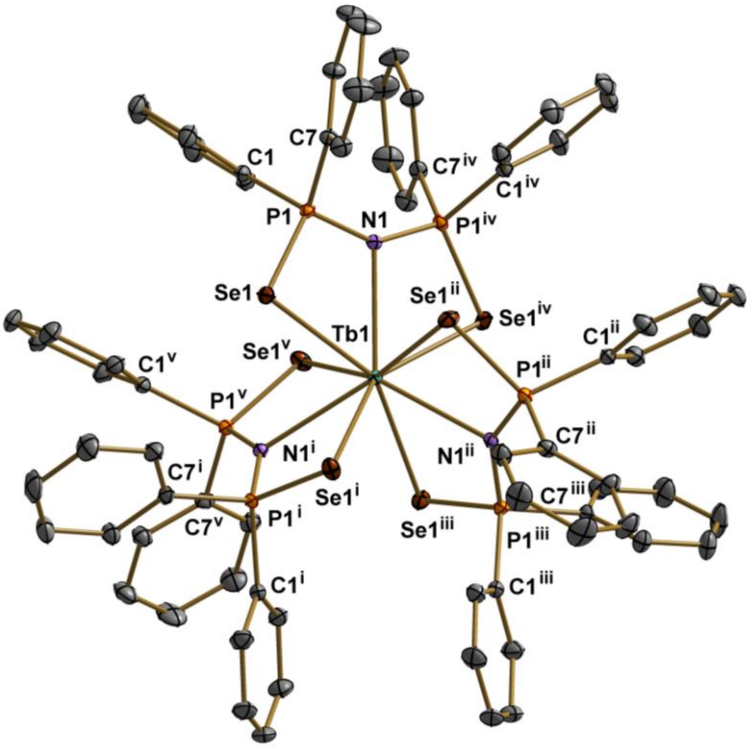

Figure S9. Ellipsoid plot for **3-Tb·tol**, drawn at the 50% probability level.

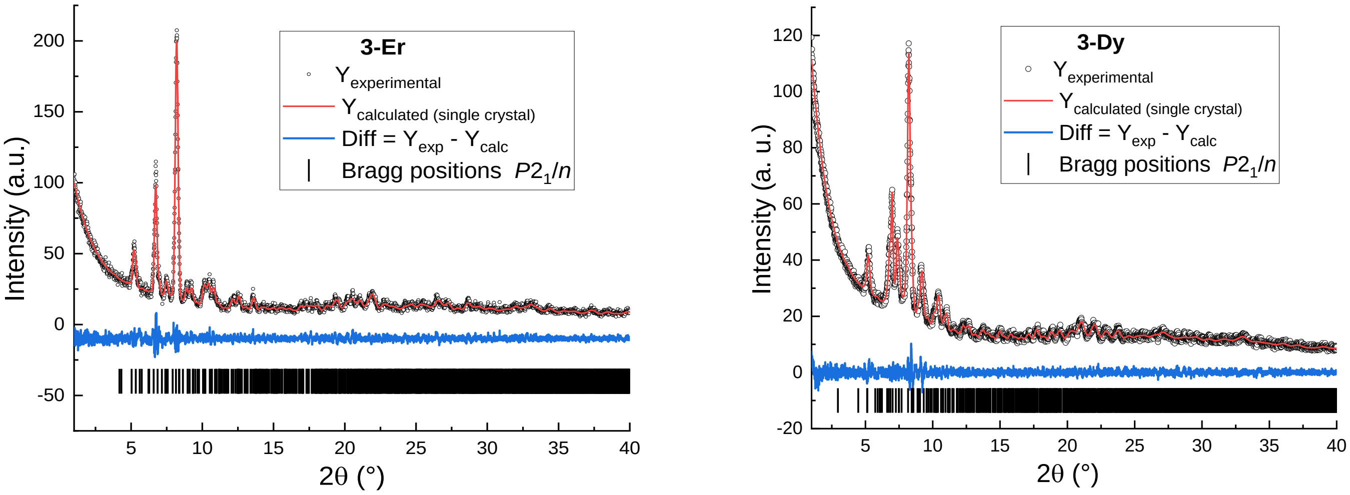

Figure S10. Calculated (red line) and experimental (black dots, Cu-Kα radiation, λ = 1.5406 Å) powder X-ray diffraction patterns for products 3-Er·3thf and 3-Dy·2thf. The experimental data were measured from the ground crystals sealed in Hilgenberg capillaries, as described in the Experimental. No change in the crystal phase profiles was observed over the measurement period of 24 h. Data refinement performed with the TOPAS software[7] confirms the presence of only one crystalline phase for each product, corresponding to those calculated from the single-crystal X-ray data (space group $P2_1/n$, Table S8).

Packing contacts in [Ln{N(SePPh$_2$)$_2$}$_3$] complexes, Ln = Dy^{3+}, Er^{3+}, and Tb^{3+}

Non-bonding Ln···Ln distances are large in all complexes, with the shortest ones ranging from about 10.8 to about 13.0 Å (Tables 1 and S12). This supports the mononuclear nature of the products and indicates that crystal packing primarily depends on weak, non-covalent contacts rather than significant Ln···Ln interactions. Due to differences in unit cell volumes, molecular geometries, lattice solvents, metal cations, and donor atoms, no clear trend appears in these distances, except that the smallest ones are observed in **1-Ln** compared to **2-Ln**. At the same time, in **3-Ln**, they depend significantly on the nature of the lattice solvent (Table S12).

Table S12. Main non-bonding intermetallic distances (Å) in the **1-Ln**, **2-Ln**, and **3-Ln** complexes

	**1-Er·0.5tol**	**1-Dy·0.5tol**	**2-Er·tol**	**2-Dy·tol**	**3-Er·3thf**	**3-Dy·2thf**	**3-Er·dcm**	**3-Dy·dcm**
Shortest distance in the unit cell	11.137	10.833	12.753	12.728	12.368	12.199	13.004	13.029
Along *a*	13.134	13.434	14.958	14.943	21.601	21.388	13.324	13.346
Along *b*	14.012	14.191	14.958	14.943	14.136	13.964	38.434	38.505
Along *c*	21.457	21.665	28.152	28.076	26.293	25.841	14.043	14.042

Tables S13-S15 and Figures S11 and S12 describe the non-covalent contacts in the [Ln{N(SePPh$_2$}$_3$] solid-state structures. For **1-Ln** and **3-Ln**, the molecular packing is stabilized by intra- and intermolecular C−H···A (A = acceptor = O, N, or Se atoms), C−H···π, and π-π interactions. These are similar in nature to those recently described by Rangarajan and co-workers for tris(imidotetraphenyldiphosphinate, tpip$^-$) chelates of Yb, Er, Gd, and Nd,[8] allowing for the effect of distinct space groups and packing arrangements on the structural parameters. Interestingly, the **2-Ln** molecules do not present any comparable interaction of these types, the shortest distances between the centroids of aromatic rings (Cg1···Cg1, with Cg1 = centroid for the C1 to C6 ring) being 4.4188(11) and 4.4117(15) Å for Ln = Dy and Er, respectively, compared to the range of 3.5672(14) to 3.795(3) Å for all other products described in this work (Table S14).

Since the crystal structures of **3-Ln** (Ln = Dy, Er, Tb) are described here for the first time, a brief discussion of their (intra- and inter)molecular interactions is provided below, with quantitative data for **3-Ln·dcm** and **3-Dy·2thf** presented in Tables S13-S15. In the case of **3-Er·3thf**, extensive disorder in the ligand Ph rings prevented anisotropic refinement of the ring carbon atoms, so contacts are only listed where anisotropy was successfully included.

Figures S11 (general view) and S12 (connections for three neighboring molecules) illustrate the intra- and intermolecular contacts involving the $[Ln\{N(SePPh_2)_2\}_3]$ compounds in the unit cell, employing **3-Er·dcm** as a representative product. Tables S13-S15, in turn, present numerical figures (distances and angles) for **1-Ln** and **3-Ln**, as the **2-Ln** complexes do not present comparable interactions.

The most significant C−H···A contacts in both **3-Er·dcm** and **3-Dy·dcm** have selenium as the hydrogen bond acceptor (A), namely the intermolecular C73−H73B···Se6^{iii} (Table S13) connecting a CH_2Cl_2 molecule and a neighboring Se atom, and two additional interactions nearly down the *b*-axis, involving aromatic hydrogens, H16 and H39, and Se3^{ii} and Se6^{i,} respectively.

C−H···π interactions, in turn, include one intramolecular and three intermolecular contacts (Table S15) for **3-Dy·dcm** and **3-Er·dcm**, with H···Cg distances of 2.75-2.97 Å, and C−H···Cg bond angles of 140-176°. A highly directional C29−H29···Cg2^{v} interaction is observed for these dcm solvates (C−H···Cg bond angles 177 and 179° for **3-Er** and **3-Dy**, respectively), which, combined with the intermolecular C16−H16···Se6^{i} contact, connects three adjacent **3-Er/Dy·dcm** molecules in the *bc*-plane (Figure S12). Connectivity between nearby molecules also exists in **3-Er·3thf** and **3-Dy·2thf** but arises solely from C−H···π contacts, as C−H···O/Se interactions are either intramolecular or involve thf molecules.

Face-to-face π-stacking is observed in all cases for **1-Er/Dy·tol**, **3-Er/Dy·dcm**, and **3-Er/Dy·thf**, with well consistent Cg···Cg distances in each set of isomorphous complexes. The shortest (strongest) contact of this type involves **3-Er·dcm** (3.5672(14) Å) and **3-Dy·dcm** molecules (3.571(6) Å, Table S14).

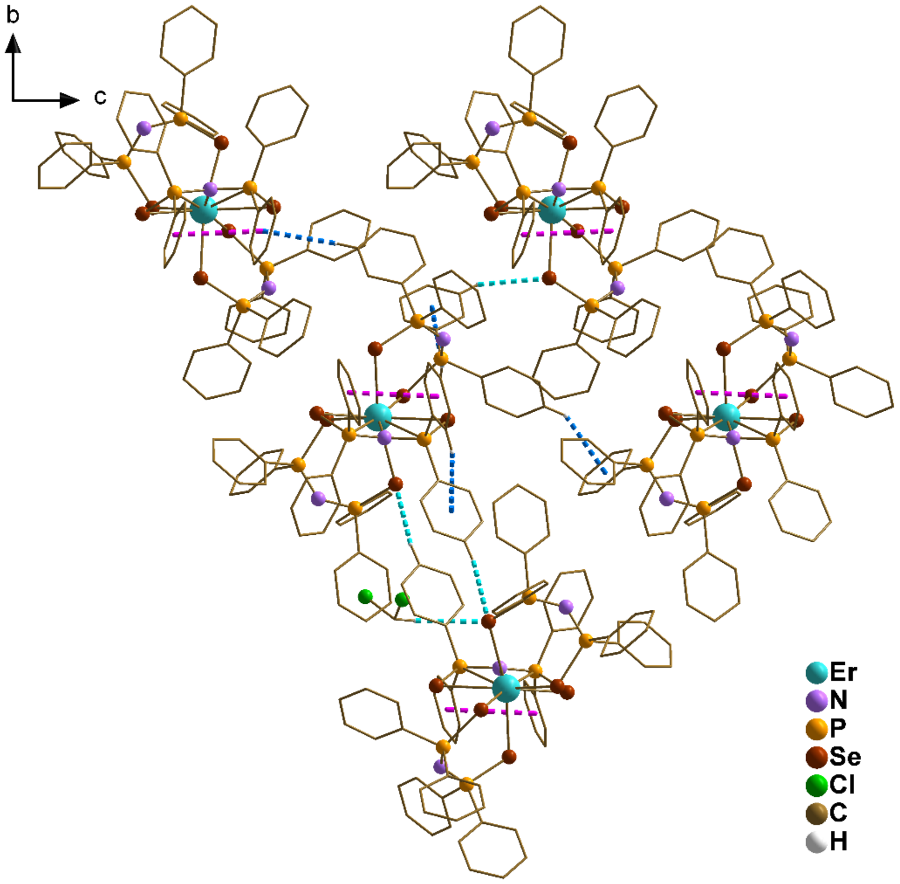

Figure S11. Ball-and-stick representation (general view) of the non-covalent intra- and intermolecular interactions (Tables S13-S15) in the three-dimensional packing of **3-Er·dcm**. The light-blue dashed lines represent the C–H···Se intermolecular contacts, while the dark-blue and the pink dashed lines depict the intra- and intermolecular C–H···π and the intramolecular π···π interactions, respectively. The hydrogen atoms not participating in the interactions were omitted for clarity.

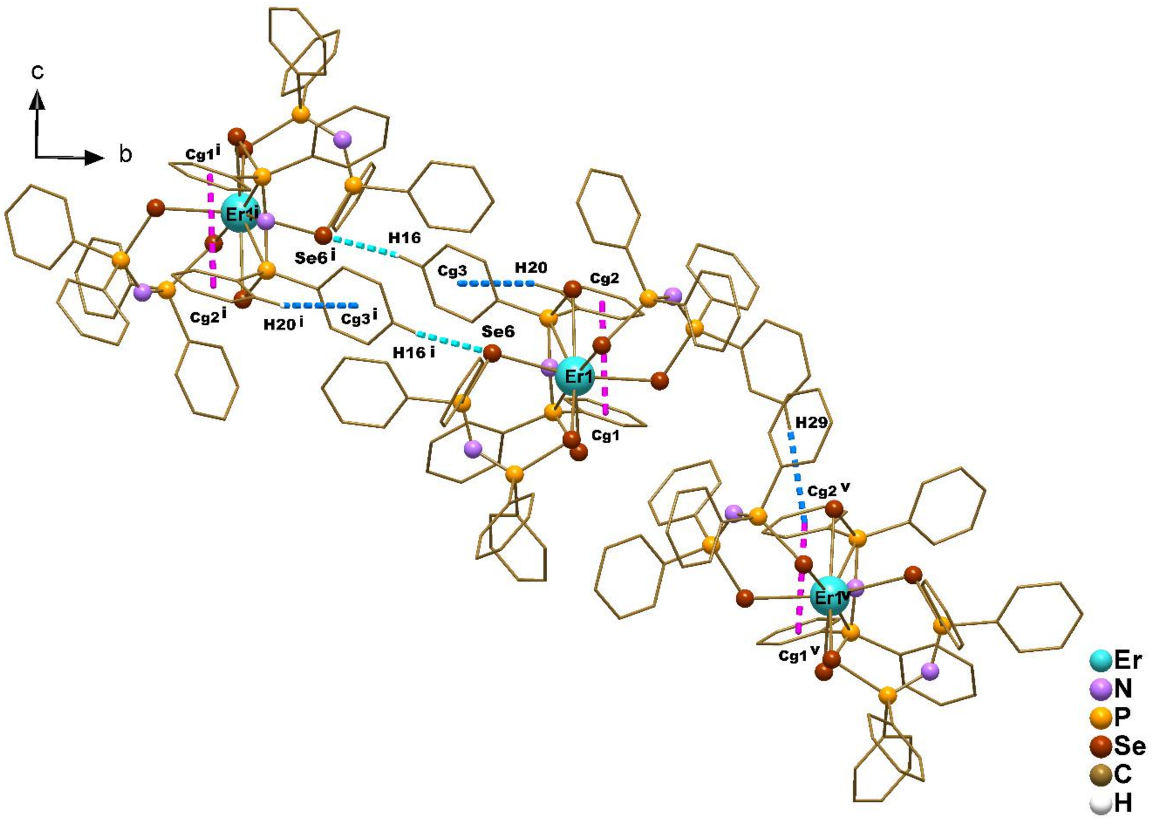

Figure S12. Detailed view of the non-covalent interactions connecting three adjacent **3-Er·dcm** molecules nearly down the *b*-axis. The light blue dashed lines represent the intermolecular C−H···Se contacts; the dark blue, the intramolecular C20−H20···Cg3 and intermolecular C29−H29···Cg2^{v} interactions (Cg2 = C19-C24; Cg3 = C13-C18), and the pink dashed lines represent the intramolecular π···π contacts between Cg1 and Cg2 (Cg1 = C1-C6). The hydrogen atoms not participating in interactions and the crystallizing solvent molecules were omitted for clarity. Symmetry codes: (i) 1−x,1−y,1−z; (v) 1/2+x,3/2-y,-1/2+z.

Table S13. Selected C−H···*acceptor* contacts for complexes **1-Dy·0.5tol**, **1-Er·0.5tol**, **3-Dy·dcm**, **3-Er·dcm**, **3-Dy·2thf**, **3-Er·3thf** and **3-Tb·tol**, with estimated standard deviations in parentheses (d = distance; D = donor; A = acceptor). Distances are expressed in Angstroms, angles in degrees. All numerical values involve anisotropically refined atoms.

**1-Dy·0.5tol**				
**D−H···A**	**d(D−H)**	**d(H···A)**	**d(D···A)**	**DHA**
**C18A−H18A···N1**	0.93	2.62	3.007(13)	106

**1-Er·0.5tol**				
**D−H···A**	**d(D−H)**	**d(H···A)**	**d(D···A)**	**DHA**
**C18−H18···N1**	0.95	2.58	2.994(7)	107
**C38−H38···O1**	0.95	2.59	3.519(3)	167

**3-Dy·dcm**				
**D−H···A**	**d(D−H)**	**d(H···A)**	**d(D···A)**	**DHA**
**C16−H16···Se6^{i}**	0.95	2.94	3.859(3)	163
**C39−H39···Se3^{ii}**	0.95	2.91	3.658(2)	137
**C73−H73b···Se6^{iii}**	0.99	2.93	3.832(4)	151

**3-Er·dcm**				
**D−H···A**	**d(D−H)**	**d(H···A)**	**d(D···A)**	**DHA**
**C16−H16···Se6^{i}**	0.95	2.94	3.860(11)	163
**C39−H39···Se3^{ii}**	0.95	2.90	3.657(8)	137
**C73−H73b···Se6^{iii}**	0.99	2.93	3.829(14)	151

**3-Dy·2thf**				
**D−H···A**	**d(D−H)**	**d(H···A)**	**d(D···A)**	**DHA**
**C2−H2···Se3**	0.95	2.94	3.833(5)	156
**C8A−H8A···O1^{i}**	0.95	2.46	3.225(15)	138
**C24−H24···Se6**	0.95	2.98	3.729(4)	137

**3-Er·3thf**				
**D−H···A**	**d(D−H)**	**d(H···A)**	**d(D···A)**	**DHA**
**C66−H66···Se6**	0.93	2.89	3.420(11)	117

**3-Tb·tol**				
**D−H···A**	**d(D−H)**	**d(H···A)**	**d(D···A)**	**<(DHA)**
**C12−H12···Se1**	0.95	2.95	3.460(2)	115

Symmetry transformations used to generate equivalent atoms:

3-Dy·CH$_2$Cl$_2$: (i) 1−x,1−y,1−z; (ii) 1/2+x,3/2− y,1/2+z; (iii) 2−x,1−y,1−z.

3-Er·CH$_2$Cl$_2$: (i) 2−x,−y,−z; (ii) −1/2+x,1/2− y,1/2−z; (iii) x,y,z+1.

3-Dy·thf: (i) 1−x,1−y,1−z.

Table S14. Selected π···π contacts (Angstroms) for complexes **1-Dy·0.5tol**, **1-Er·0.5tol**, **3-Dy·dcm**, **3-Er·dcm**, **3-Dy·2thf**, and **3-Er·3thf**, with estimated standard deviations in parentheses (d = distance; Cg = centroid of the aromatic ring). The carbon atoms in the rings involved in these π···π interactions were refined anisotropically.

**1-Dy·0.5tol**		**1-Er·0.5tol**	
**d(Cg···Cg)**	**d(Cg···Cg)**	**Cg···Cg**	**d(Cg···Cg)**
**Cg1···Cg1^{i}**	3.777(4)	**Cg1···Cg1^{i}**	3.7793(13)
**3-Dy·dcm**		**3-Er·dcm**	
**Cg···Cg**	**d(Cg···Cg)**	**Cg···Cg**	**d(Cg···Cg)**
**Cg1···Cg2**	3.5672(14)	**Cg1···Cg2**	3.571(6)
**3-Dy·2thf**		**3-Er·3thf**	
**Cg···Cg**	**d(Cg···Cg)**	**Cg···Cg**	**d(Cg···Cg)**
**Cg1···Cg2**	3.795(3)	**Cg1···Cg2**	3.776(7)

For **1-Dy** and **1-Er**, Cg1 = centroid for the C43 to C48 ring.
For **3-Dy·dcm** and **3-Er·dcm**, Cg1 = C1 to C6; Cg2 = C19 to C24.
For **3-Dy·thf** and **3-Er·thf**, Cg1 = C1 to C6; Cg2 = C13 to C18.
Symmetry transformations used to generate equivalent atoms (**1-Dy** and **1-Er**):
x,1−y,1−z.

Table S15. Selected C−H···π contacts for complexes **1-Dy·0.5tol**, **1-Er·0.5tol**, **3-Dy·dcm**, **3-Er·dcm**, **3-Dy·2thf** and **3-Er·3thf** (d = distance; Cg = centroid of the aromatic ring). Distances are expressed in Angstroms, angles in degrees. The carbon atoms involved in these C−H···π interactions were refined anisotropically.

**1-Dy·0.5tol**		
**C−H···Cg**	**d(H···Cg)**	**X−H···Cg**
**C6−H6···Cg2^{i}**	2.79	152
**C22−H22···Cg3^{ii}**	2.79	146
**C28−H28···Cg4^{iii}**	2.86	151
**C39−H39···Cg5**	2.86	146
**C41−H41···Cg6^{iv}**	2.91	157

**1-Er·0.5tol**		
**C−H···Cg**	**d(H···Cg)**	**X−H···Cg**
**C6−H6···Cg2^{i}**	2.67	149
**C22−H22···Cg3^{ii}**	2.59	145
**C27−H27···Cg5^{iii}**	2.91	160
**C28−H28···Cg4^{iii}**	2.70	154
**C39−H39···Cg5**	2.80	146
**C41−H41···Cg6^{iv}**	2.77	155
**C45−H45···Cg7^{v}**	2.91	143
**C70−H70···Cg2^{ii}**	2.93	153

**3-Dy·dcm**		
**C−H···Cg**	**d(H···Cg)**	**X−H···Cg**
**C20−H20···Cg3**	2.97	140
**C22−H22···Cg4^{iv}**	2.75	150
**C29−H29···Cg2^{v}**	2.93	179
**C46−H46···Cg5^{vi}**	2.84	149

**3-Er·dcm**		
**C−H···Cg**	**d(H···Cg)**	**X−H···Cg**
**C20−H20···Cg3**	2.97	140
**C22−H22···Cg4^{iv}**	2.75	150
**C29−H29···Cg2^{v}**	2.96	177
**C46−H46···Cg5^{vi}**	2.82	148

**3-Dy·2thf**		
**C−H···Cg**	**d(H···Cg)**	**X−H···Cg**
**C53−H53···Cg2^{ii}**	2.71	139
**C58−H58···Cg3^{iii}**	2.76	139
**C70−H70···Cg4^{iv}**	2.92	148
**C75−H75b···Cg5^{iv}**	2.65	148

For **1-Dy** and **1-Er**: Cg2 = centroid for the C25 to C30 ring; Cg3 = C37 to C42; Cg4 = C7 to C12; Cg5 = C1 to C6; Cg6 = C49 to C54; Cg7 = C31 to C36.
For **3-Dy·CH_2Cl_2** and **3-Er·CH_2Cl_2**: Cg2 = C19 to C24, Cg3 = C13 to C18, Cg4 = C37 to C42, Cg5 = C55 to C60.
For **3-Dy·2thf**: Cg2 = C13 to C18, Cg3 = C61 to C66, Cg4 = C25 to C30, Cg5 = C31 to C36.

Symmetry transformations used to generate equivalent atoms:
1-Dy and **1-Er**: (ii) 1+x,y,z; (iii) -x,-y+2,-z; (iv) x-1,y+1,z ; (v) -x,-y+1+1,-z+1.
3-Dy·dcm: (iv) -1+x,y,z; (v) 1/2+x,3/2-y,-1/2+z ; (vi) x,y,1+z.
3-Er·dcm: (iv) 1+x,y,z; (v) −1/2+x,1/2−y,1/2+z; (vi) x,y,−1+z;
3-Dy·2thf: (ii) x,-1+y,z; (iii) 1/2-x,-1/2+y,1/2-z; (iv) -1/2+x,1/2-y,1/2+z.

3. Infrared Spectroscopy

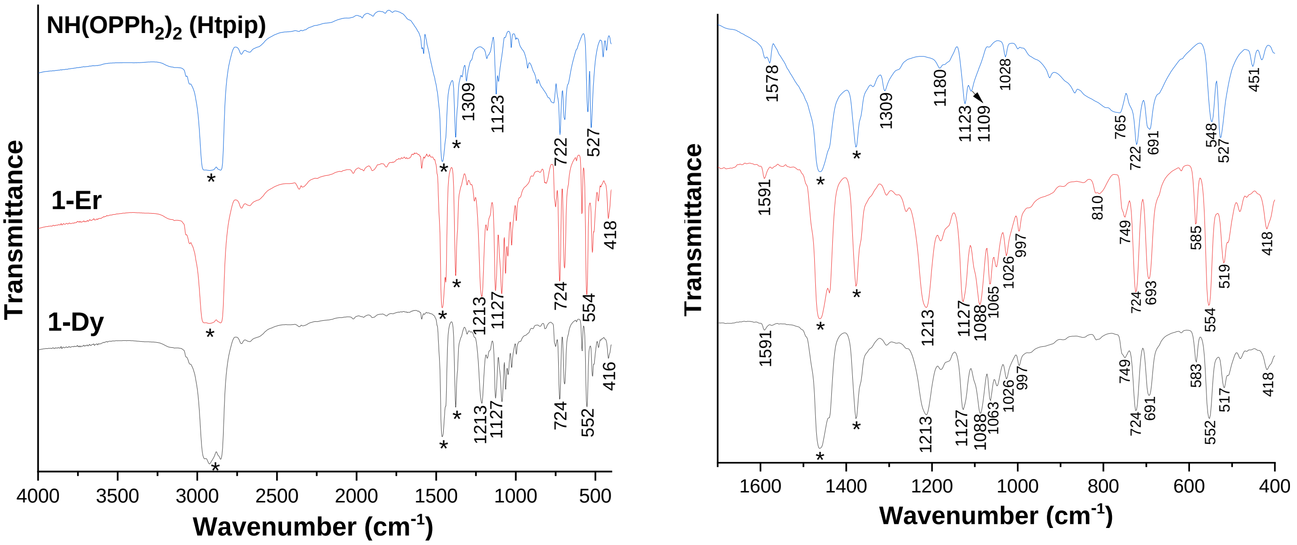

Figure S13. Left: Overview of the infrared absorption spectra (4000–400 cm^{-1}) of $NH(OPPh_2)_2$ (Htpip) and products **1-Er** and **1-Dy**. Right: Expansion of the region between 1650 and 400 cm^{-1}. The asterisks identify the absorption bands of the mineral oil (Nujol).

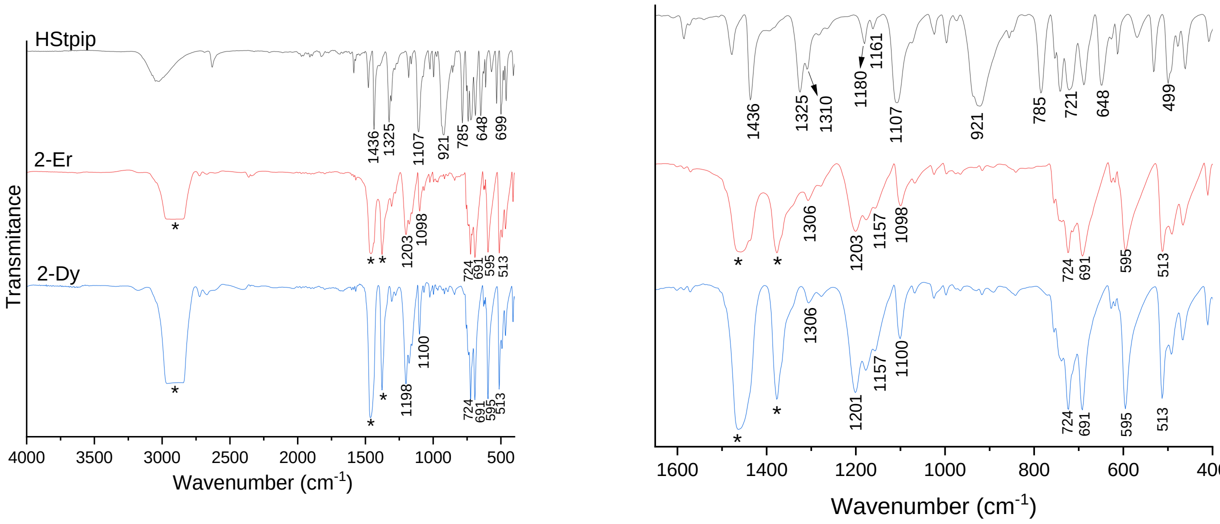

Figure S14. Comparison of the FTIR spectra of the HStpip proligand and the derived 2-Er and 2-Dy complexes in the 4000 to 400 cm^{-1} range (left) and expanded fingerprint region (1650 to 400 cm^{-1}, right). The asterisks identify the absorption bands of the mineral oil (Nujol).

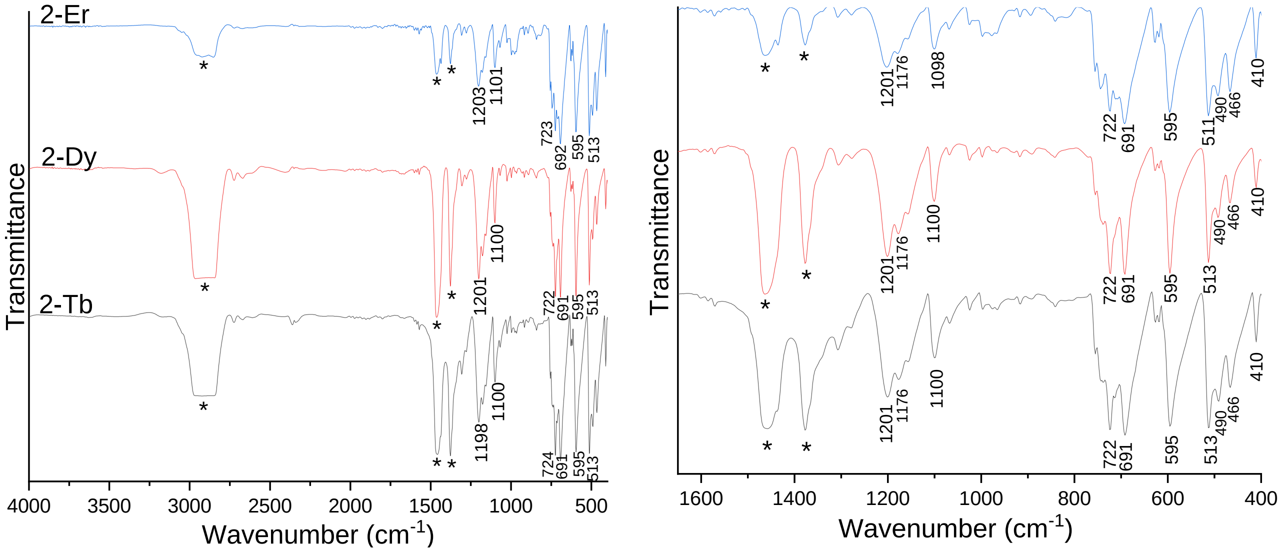

Figure S15. Left: Overview of the medium infrared absorption spectra of **2-Er**, **2-Dy**, and **2-Tb**. Right: Expanded view between 1650 and 400 cm^{-1}. The asterisks identify the absorption bands of the mineral oil (Nujol).

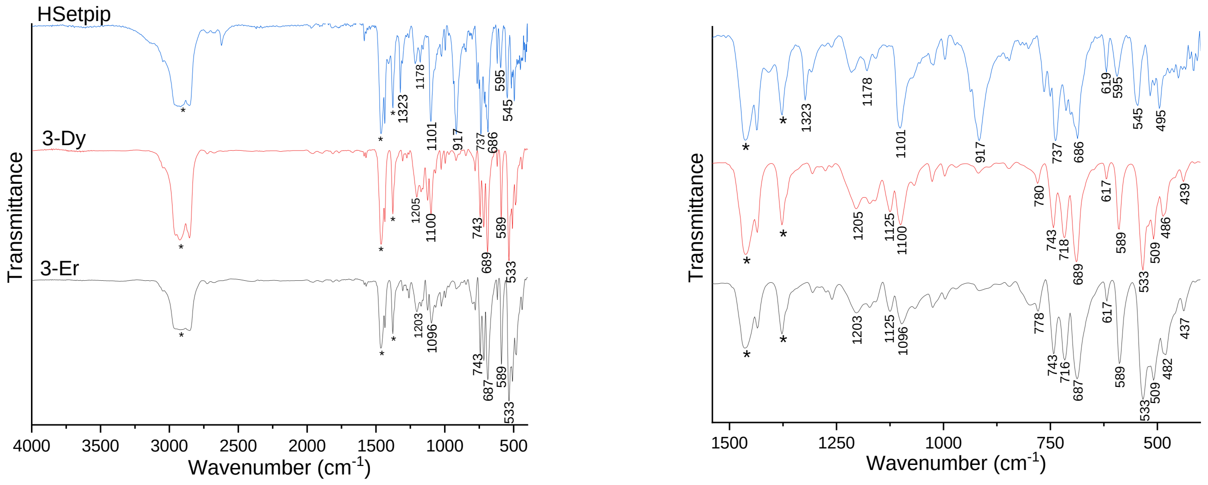

Figure S16. Left: FTIR spectra (4000–400 cm^{-1}) of products **3-Er** and **3-Dy**, recorded in Nujol mulls. Right: Detailed view of the fingerprint region (1550-400 cm^{-1}). Asterisks indicate the mineral oil absorption bands (Nujol).

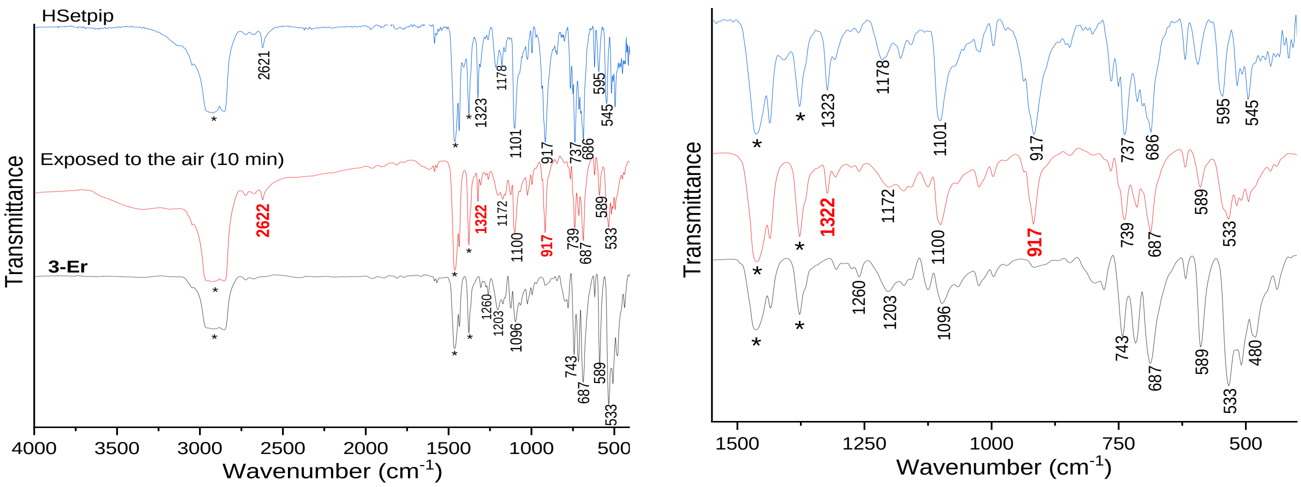

Figure S17. Left: FTIR spectrum (4000-400 cm^{-1}) of product 3-Er after exposure to the air for 10 min, compared with the proligand (HSetpip) and a freshly prepared sample of the same complex. Right: Detailed view of the fingerprint region (1550-400 cm^{-1}). The highlighted (bold red) wavenumbers mark the free HSetpip bands at 2622 and 1322 cm^{-1}, ν(N–H) and δ(N–H) respectively, and 917 cm^{-1}, $\nu_{as}(P_2NH)$,[9] which appear in the spectrum of the exposed product. Asterisks indicate the mineral oil absorption bands (Nujol).

4. DC Magnetometry

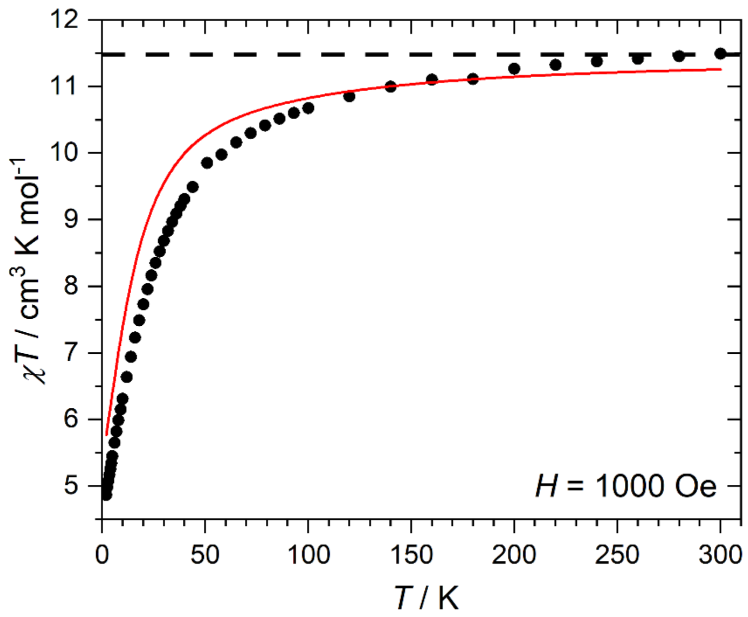

Figure S18. Temperature dependence of χT for **1-Er·0.5tol** (black circles), with CASSCF calculated curve (solid red line) and theoretical Er^{III} single ion contribution (dashed black line).

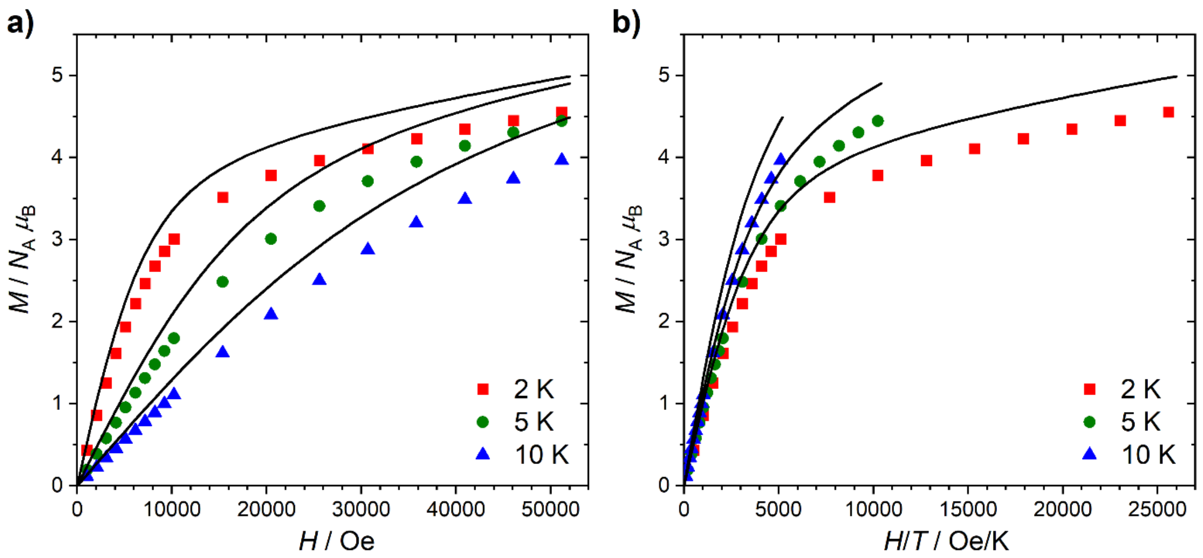

Figure S19. (a) Magnetisation *vs* field and **(b)** Reduced magnetisation *vs* field for **1-Er·0.5tol** at 2, 5 and 10 K, with CASSCF calculated curves (solid black lines).

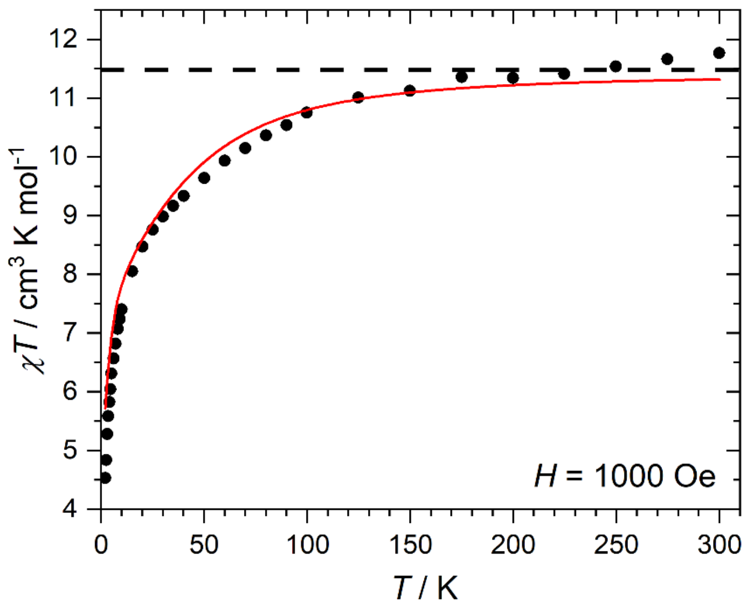

Figure S20. Temperature dependence of χT for **2-Er·tol** (black circles), with CASSCF calculated curve (solid red line) and theoretical Dy^{III} single ion contribution (dashed black line).

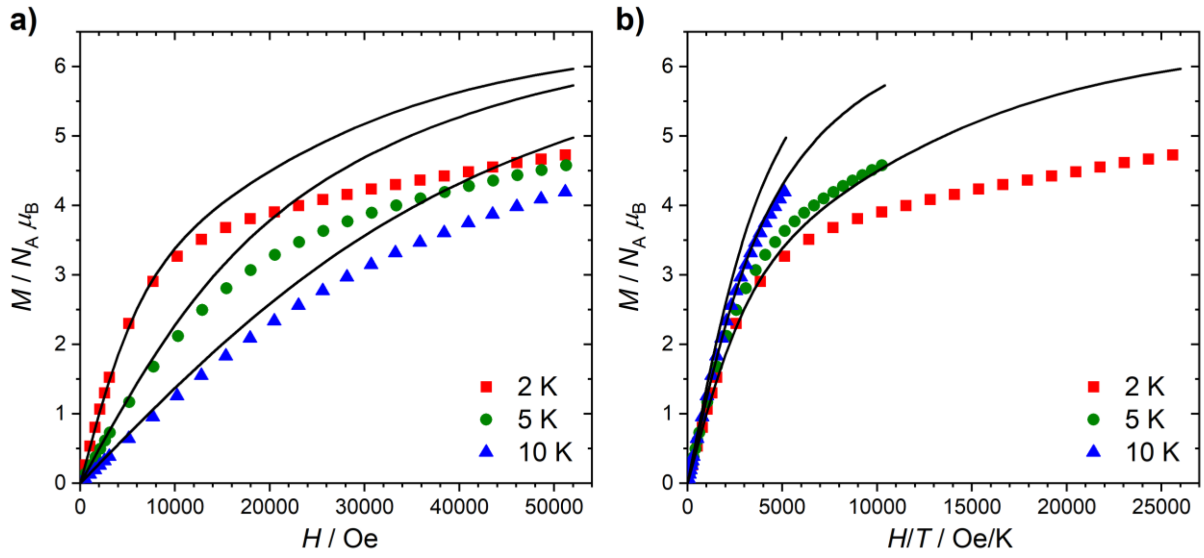

Figure S21. (a) Magnetisation *vs* field and **(b)** Reduced magnetisation *vs* field for **2-Er·tol** at 2, 5 and 10 K, with CASSCF calculated curves (solid black lines).

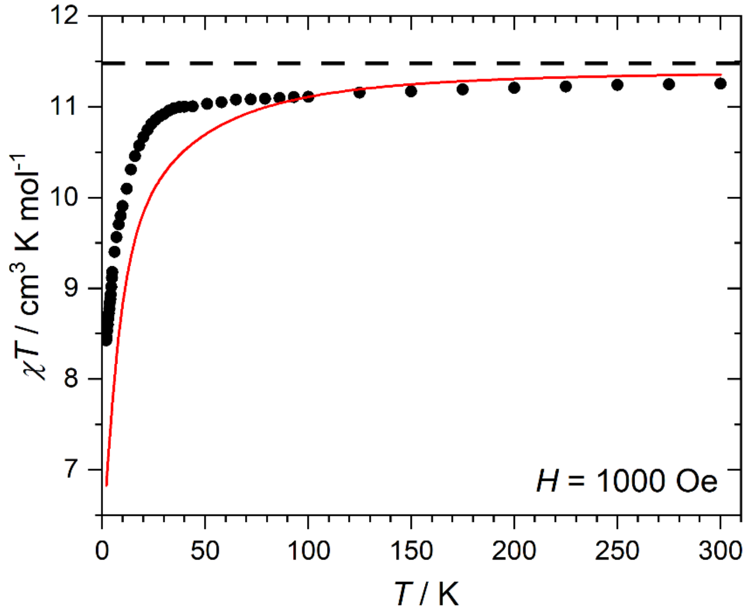

Figure S22. Temperature dependence of χT for **3-Er·dcm** (black circles), with CASSCF calculated curve (solid red line) and theoretical Dy^{III} single ion contribution (dashed black line).

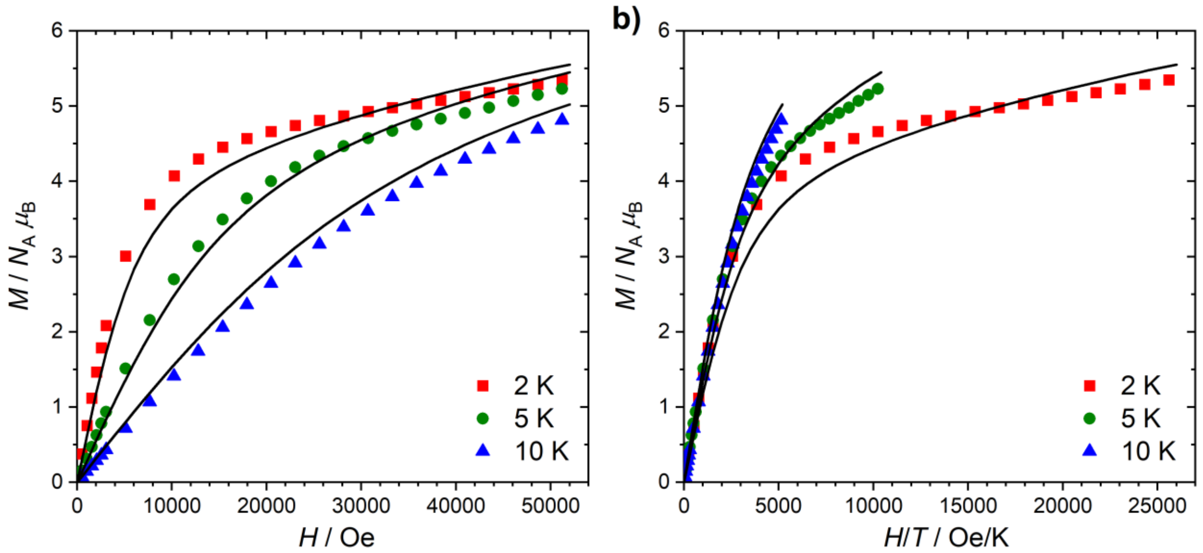

Figure S23. (a) Magnetisation *vs* field and **(b)** Reduced magnetisation *vs* field for **3-Er·dcm** at 2, 5 and 10 K, with CASSCF calculated curves (solid black lines).

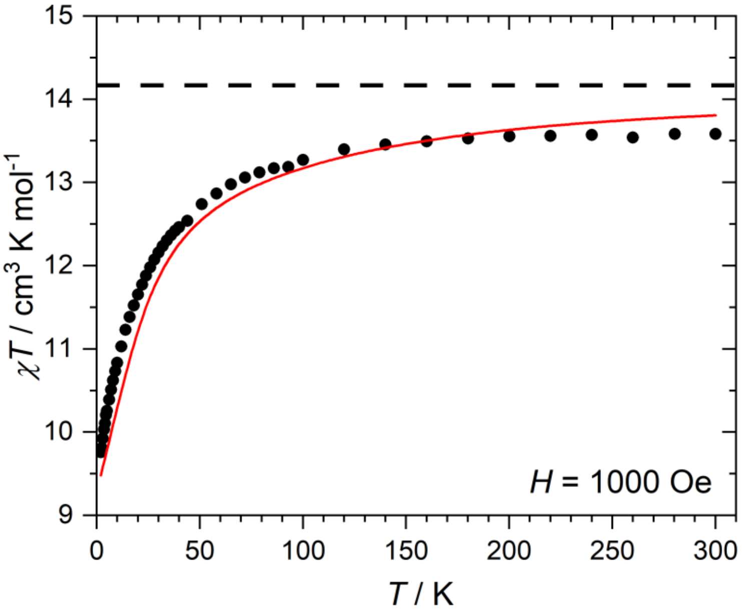

Figure S24. Temperature dependence of χT for **1-Dy·0.5tol** (black circles), with CASSCF calculated curve (solid red line) and theoretical Dy^{III} single ion contribution (dashed black line).

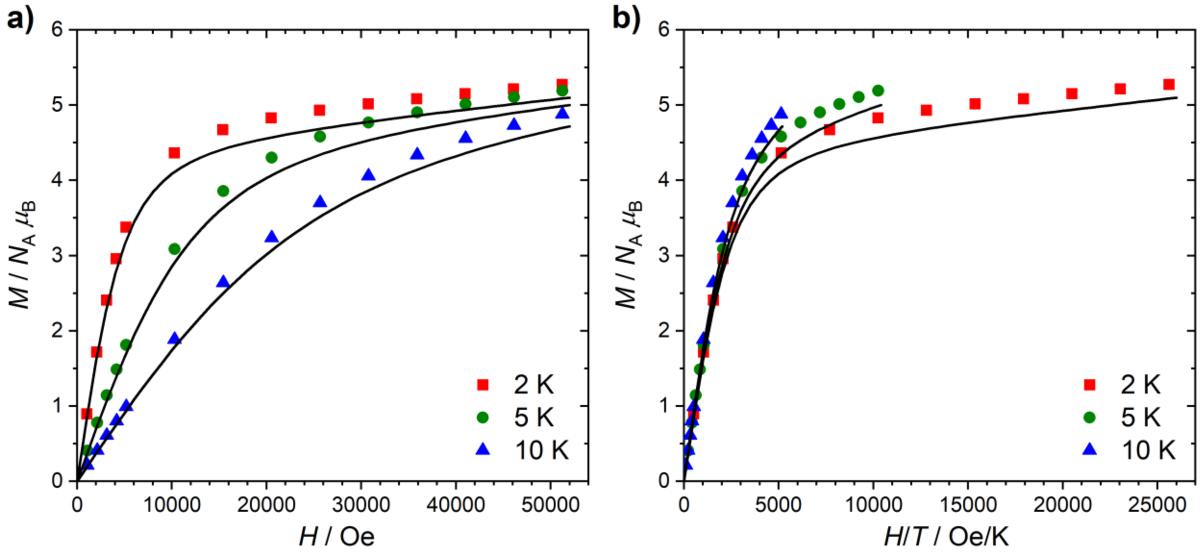

Figure S25. (a) Magnetisation *vs* field and **(b)** Reduced magnetisation *vs* field for **1-Dy·0.5tol** at 2, 5 and 10 K, with CASSCF calculated curves (solid black lines).

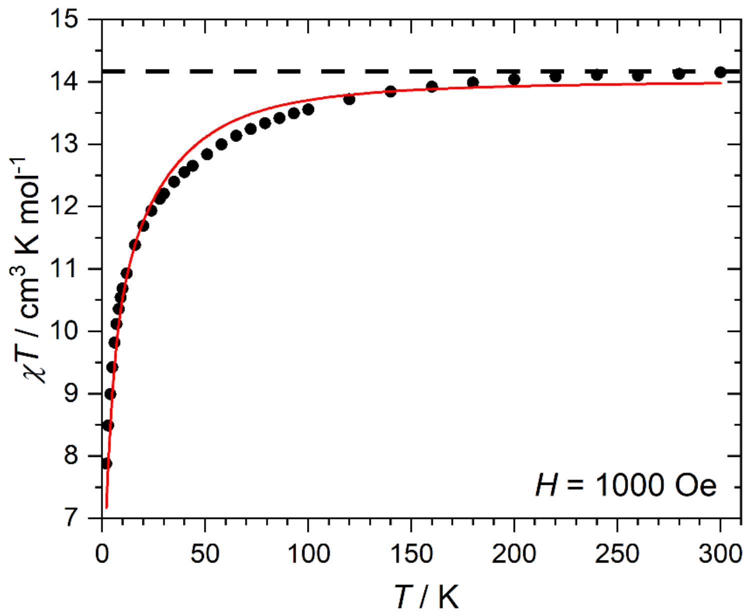

Figure S26. Temperature dependence of χT for **2-Dy·tol** (black circles), with CASSCF calculated curve (solid red line) and theoretical Dy^{III} single ion contribution (dashed black line).

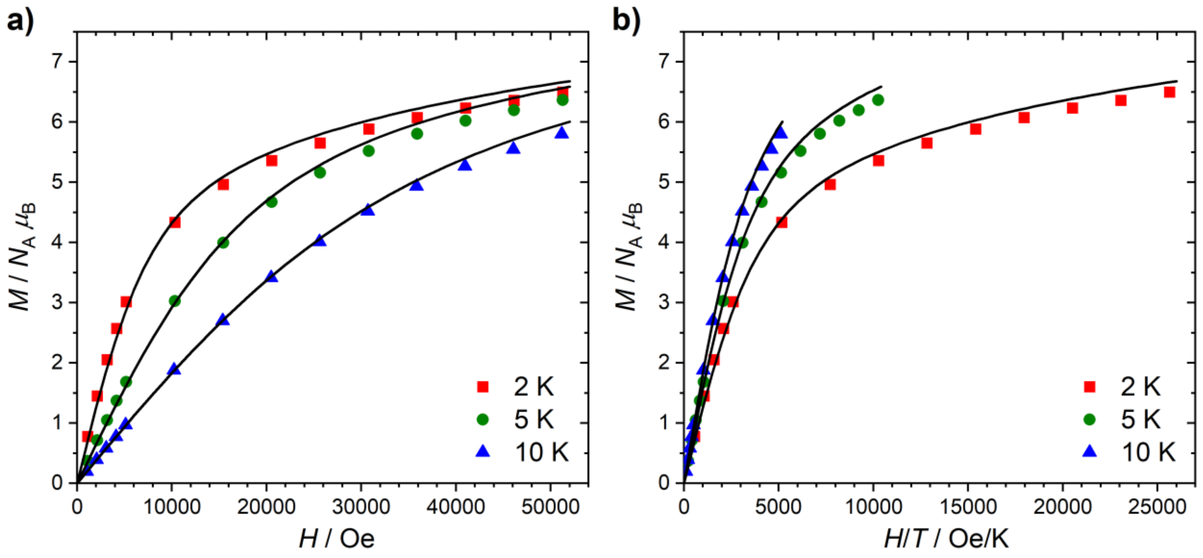

Figure S27. (a) Magnetisation *vs* field and **(b)** Reduced magnetisation *vs* field for **2-Dy·tol** at 2, 5 and 10 K, with CASSCF calculated curves (solid black lines).

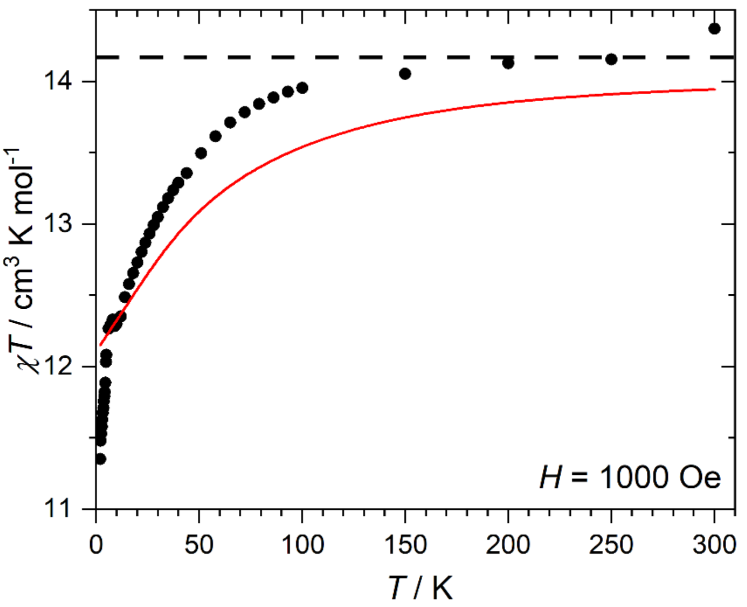

Figure S28. Temperature dependence of χT for **3-Dy·2thf** (black circles), with CASSCF calculated curve (solid red line) and theoretical Dy^{III} single ion contribution (dashed black line).

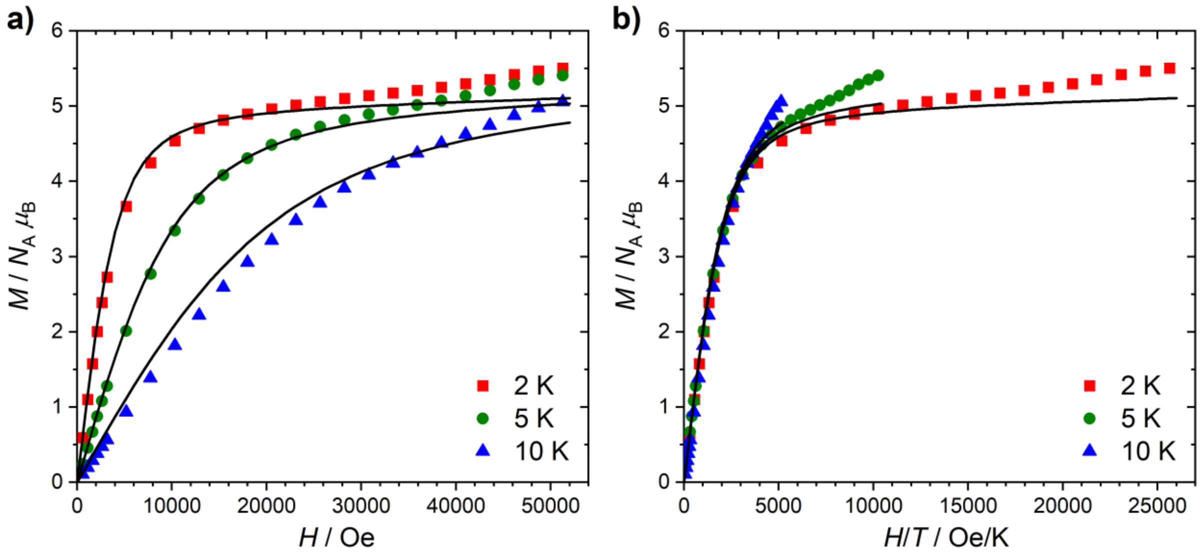

Figure S29. **(a)** Magnetisation *vs* field and **(b)** Reduced magnetisation *vs* field for **3-Dy·2thf** at 2, 5 and 10 K, with CASSCF calculated curves (solid black lines).

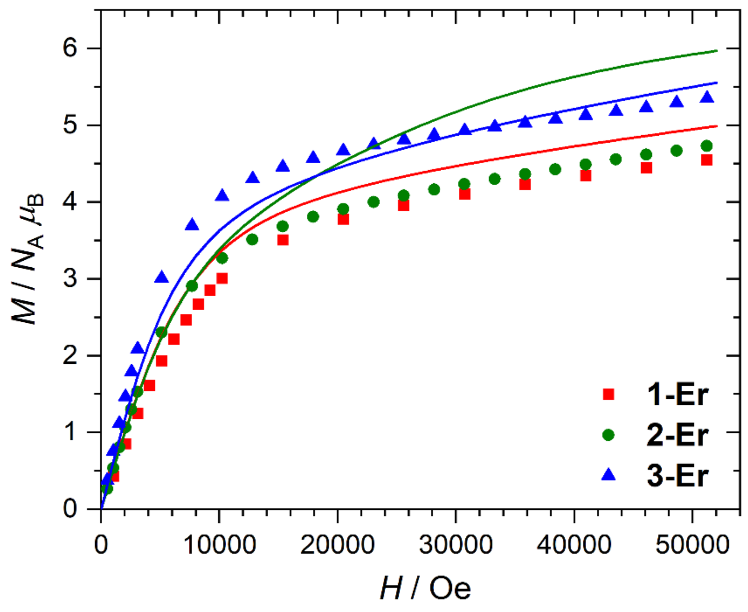

Figure S30. Magnetisation *vs* field for **1-Er·0.5tol**, **2-Er·tol** and **3-Er·dcm** at 2 K, with CASSCF calculated curves.

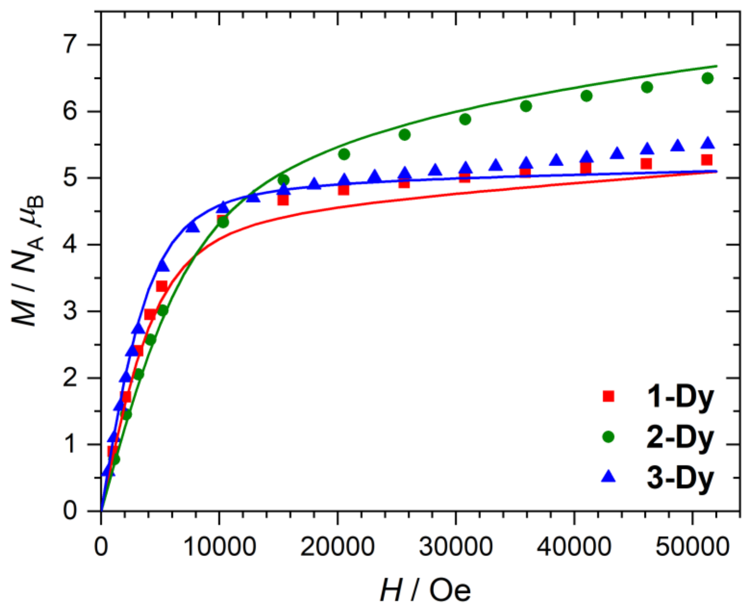

Figure S31. Magnetisation *vs* field for **1-Dy·0.5tol**, **2-Dy·tol** and **3-Dy·2thf** at 2 K, with CASSCF calculated curves.

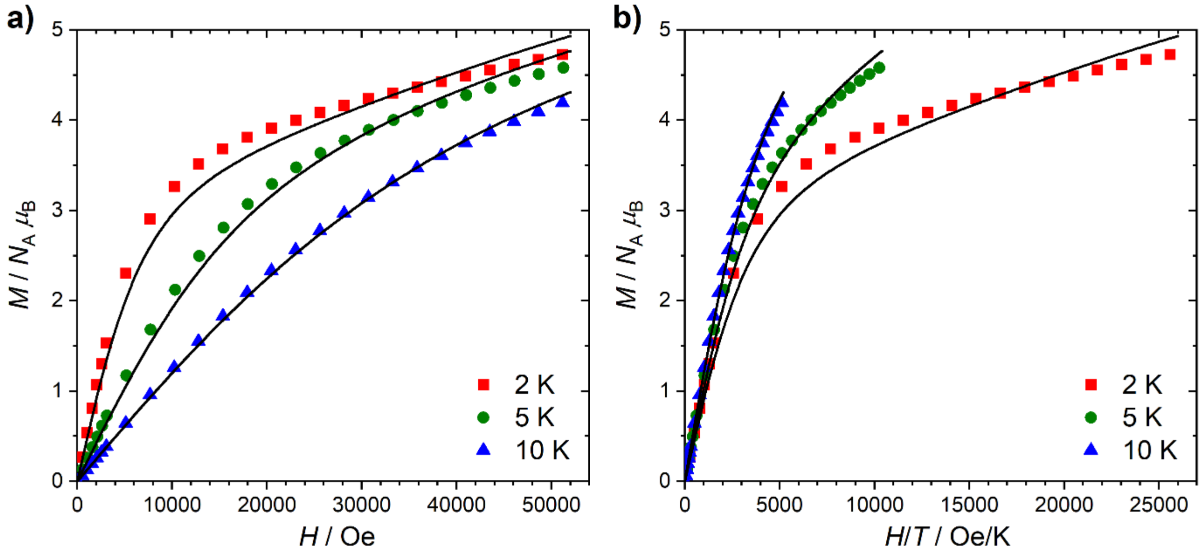

Figure S32. (a) Magnetisation *vs* field and **(b)** Reduced magnetisation *vs* field for **2-Er·tol** at 2, 5 and 10 K, with magnetisation calculated from CASSCF. CF parameters scaled by 2.15 (solid black lines), corresponding to a first excited state at 19.1 cm^{-1}.

5. CASSCF-SO Calculations

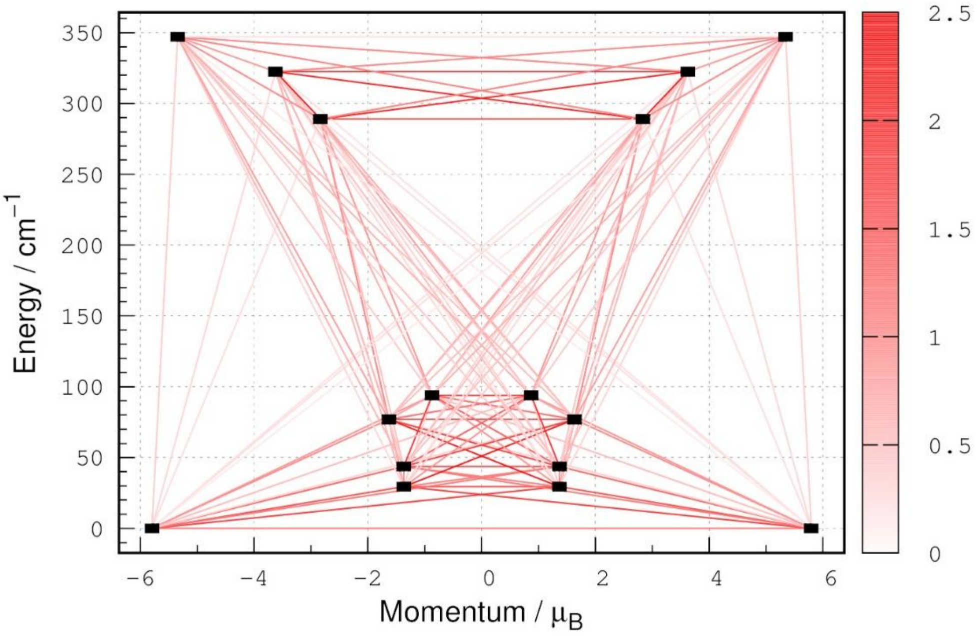

Figure S33. Computed transition matrix elements for **1Er·0.5tol** based on CASSCF-SO calculations. The thick black lines represent the Kramers doublets and the shade of red of the line is proportional to the transition probability between the states connected by the line.

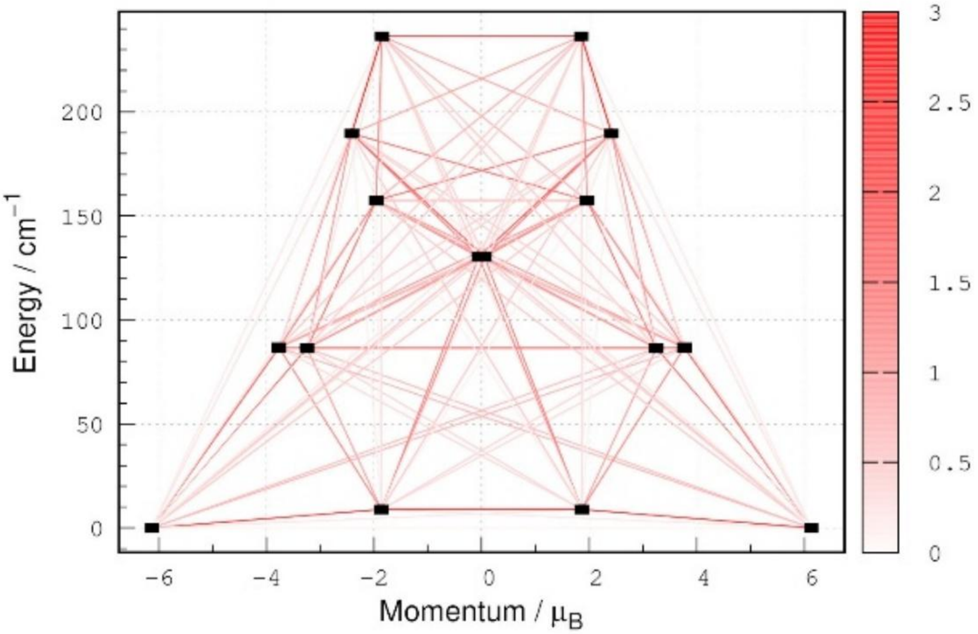

Figure S34. Computed transition matrix elements for **2Er·tol** based on CASSCF-SO calculations. The thick black lines represent the Kramers doublets and the shade of red of the line is proportional to the transition probability between the states connected by the line.

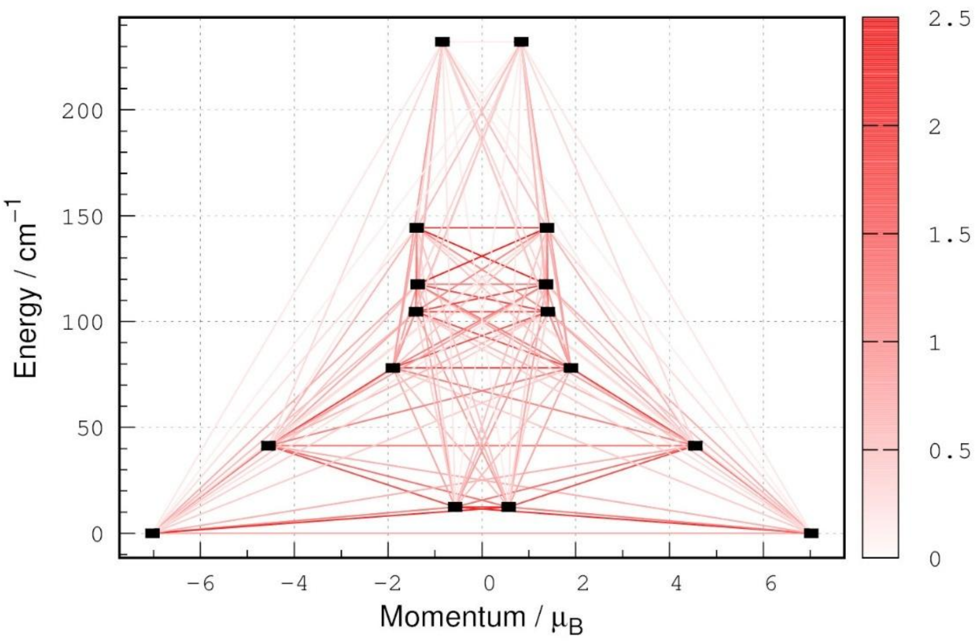

Figure S35. Computed transition matrix elements for **3Er·3thf** based on CASSCF-SO calculations. The thick black lines represent the Kramers doublets and the shade of red of the line is proportional to the transition probability between the states connected by the line.

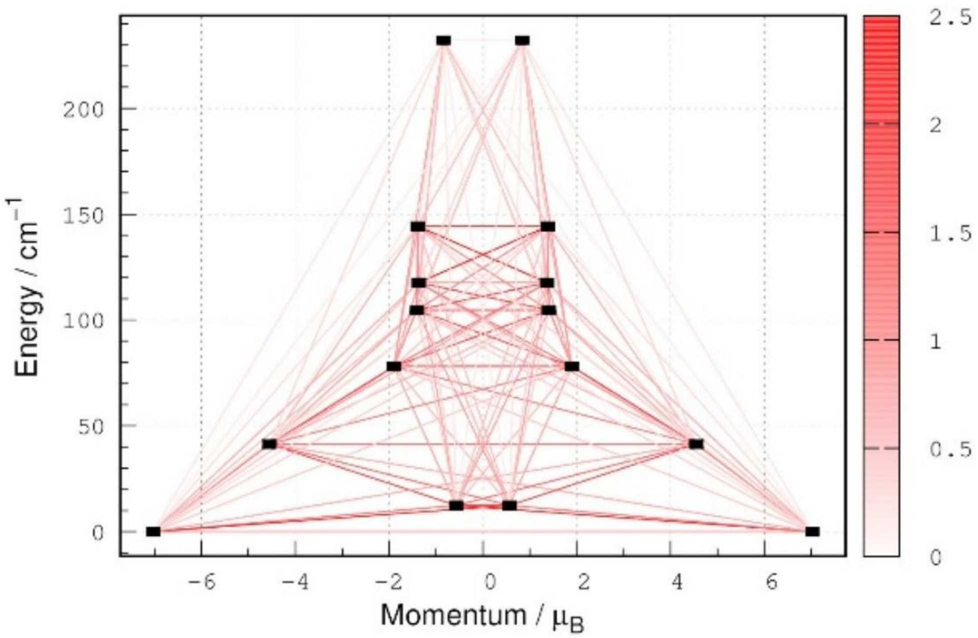

Figure S36. Computed transition matrix elements for **3Er·dcm** based on CASSCF-SO calculations. The thick black lines represent the Kramers doublets and the shade of red of the line is proportional to the transition probability between the states connected by the line.

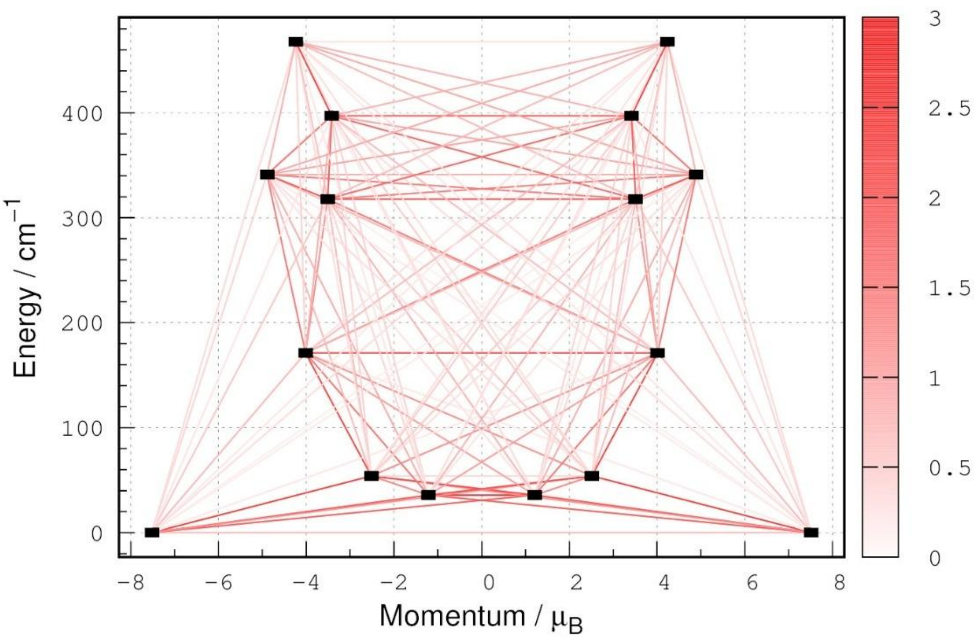

Figure S37. Computed transition matrix elements for **1Dy ·0.5tol** based on CASSCF-SO calculations. The thick black lines represent the Kramers doublets and the shade of red of the line is proportional to the transition probability between the states connected by the line.

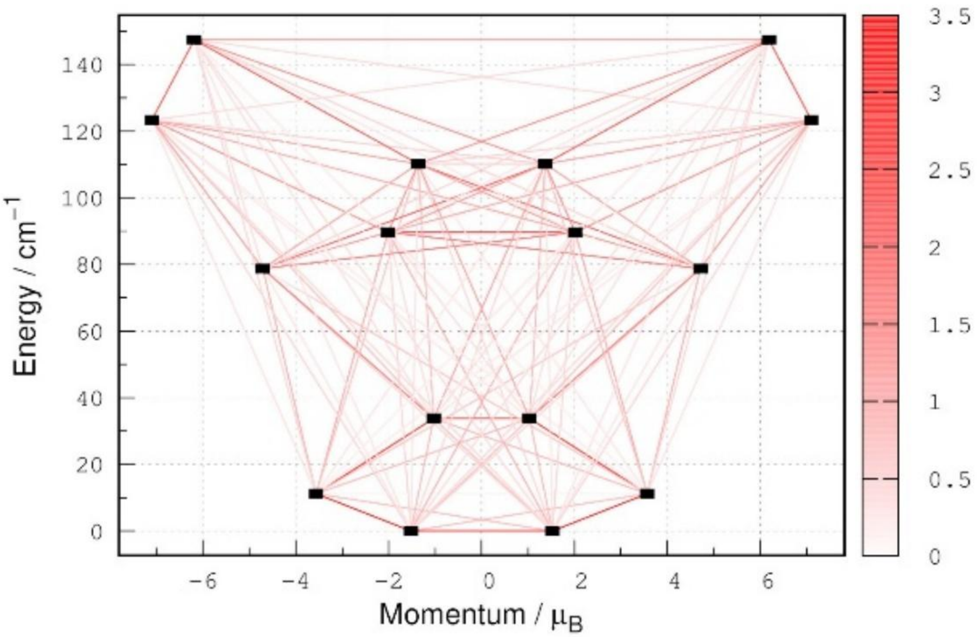

Figure S38. Computed transition matrix elements for **2Dy·tol** based on CASSCF-SO calculations. The thick black lines represent the Kramers doublets and the shade of red of the line is proportional to the transition probability between the states connected by the line.

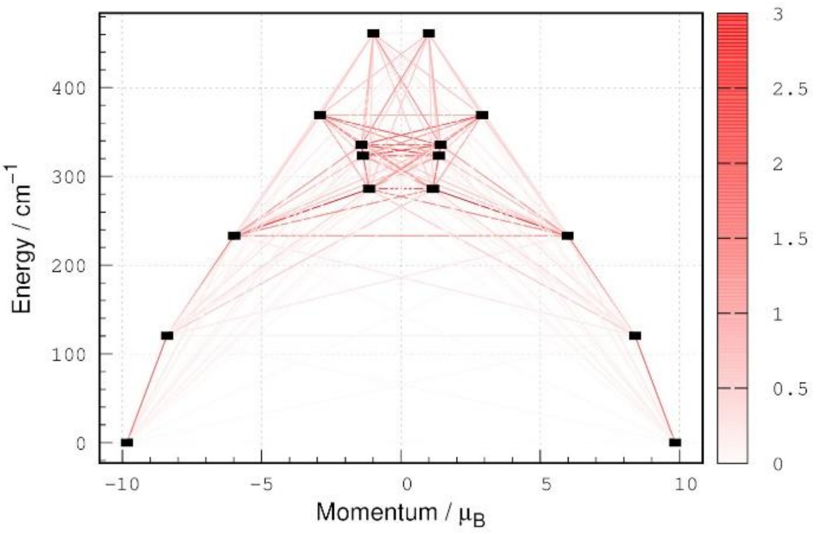

Figure S39. Computed transition matrix elements for **3Dy·2thf** based on CASSCF-SO calculations. The thick black lines represent the Kramers doublets and the shade of red of the line is proportional to the transition probability between the states connected by the line.

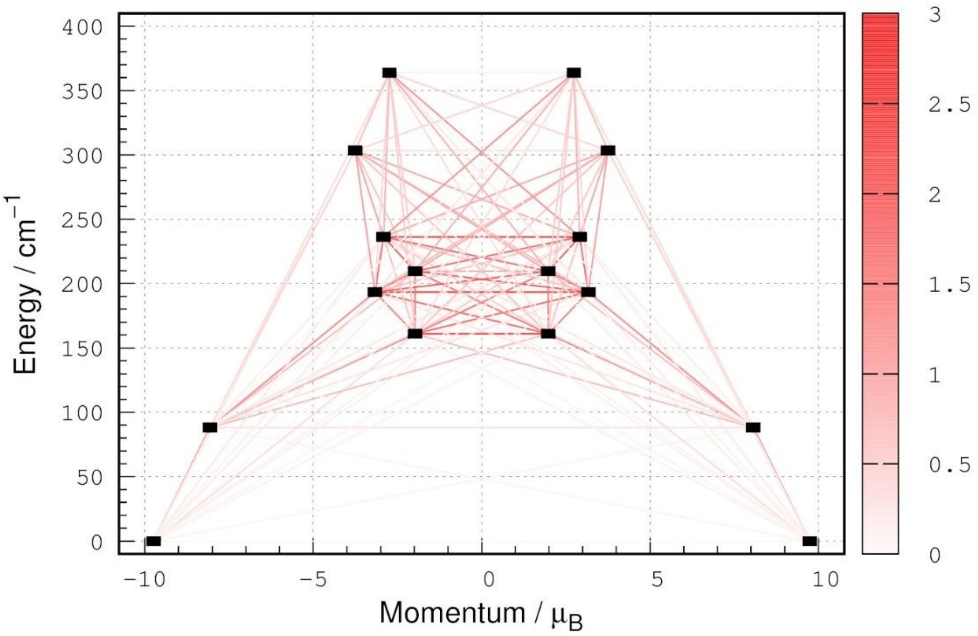

Figure S40. Computed transition matrix elements for **3Dy·dcm** based on CASSCF-SO calculations. The thick black lines represent the Kramers doublets and the shade of red of the line is proportional to the transition probability between the states connected by the line.

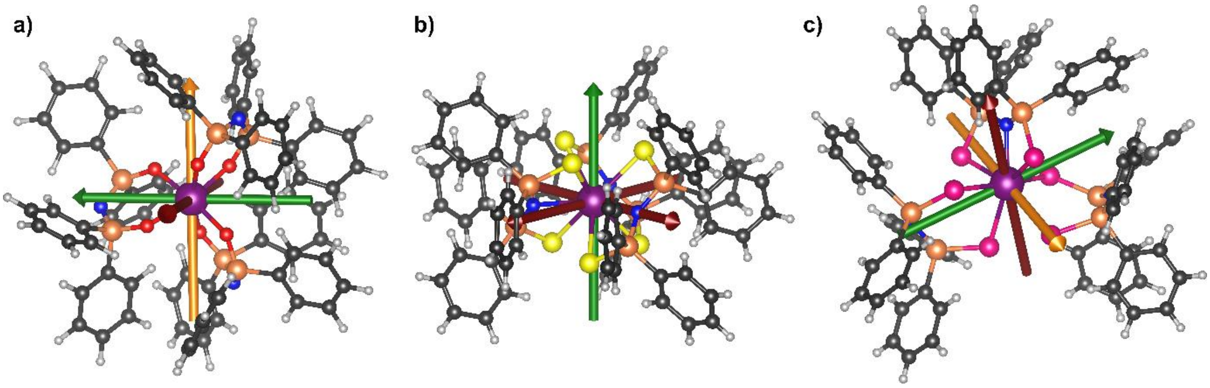

Figure S41. Ground-state anisotropy axes for (a) **1-Er·0.5tol**, (b) **2-Er·tol** and (c) **3-Er·dcm** calculated by CASSCF-SO. Easy axis (green), intermediate axis (orange), hard axis (maroon). Color code: Er (purple), N (blue), P (peach), O (red), S (yellow), Se (pink), C (dark grey), H (light grey).

Table S16. CASSCF-SO calculated states of the ground $^4I_{15/2}$ term for **1-Er·0.5tol**, showing g-tensor components, ground state composition, angle between the ground state z axis and the excited states z axis, and expectation value of the J_z operator.

Energy / cm^{-1}	Energy / K	g_z	g_y	g_x	Composition[a]	g_z angle / °	$<J_z>$
0	0	11.5909	5.8412	1.5629	45%|±15/2> + 19%|±11/2> + 5.7%|±5/2> + 5.6%|±3/2> + 4.7%|±1/2> + 4.5%|∓3/2> + 3.2%|∓15/2> + 2.7%|±9/2> + 2.2%|∓9/2>	-	±5.7955
29.4365	20.4594	8.2019	4.3582	1.6878	24%|±7/2> + 10%|∓15/2> + 10%|±9/2> + 9.8%|∓5/2> + 7.0%|±1/2> + 6.1%|∓13/2> + 6.0%|∓9/2> + 5.4%|±13/2> + 4.2%|∓7/2> + 3.6%|±5/2> + 3.5%|∓11/2> + 3.3%|±15/2> + 3.2%|∓1/2> + 2.1%|±11/2>	76.5	±1.3672
43.6104	30.3107	8.3257	3.4300	0.9351	17%|±9/2> + 17%|±3/2> + 15%|∓5/2> + 10%|±7/2> + 7.8%|±5/2> + 5.4%|±11/2> + 5.0%|±1/2> + 4.7%|∓1/2> + 4.4%|∓3/2> + 3.9%|∓15/2> + 3.1%|∓13/2>	80.2	±1.3688
76.9681	53.4955	12.3031	2.6987	0.9207	16%|±13/2> + 16%|±5/2> + 10%|±9/2> + 9.7%|±7/2> + 9.0%|∓3/2> + 8.5%|∓7/2> + 7.8%|∓15/2> + 5.0%|∓9/2> + 4.4%|±3/2> + 3.8%|∓13/2> + 3.2%|∓11/2> + 2.8%|∓1/2>	85.6	±1.6329
93.9067	65.2684	2.2410	7.2959	8.1944	21%|±3/2> + 20%|∓1/2> + 17%|±1/2> + 9.9%|∓5/2> + 6.3%|±15/2> + 5.6%|±7/2> + 4.4%|∓7/2> + 3.3%|∓9/2> + 3.3%|±5/2> + 3.2%|∓13/2>	48.5	±0.8707
289.0283	200.8847	0.9646	8.2789	9.7035	15%|±9/2> + 13%|±7/2> + 10%|∓1/2> + 10%|±11/2> + 8.8%|±15/2> + 8.2%|∓9/2> + 8.0%|±13/2> + 5.7%|∓3/2> + 5.7%|±3/2> + 5.2%|±1/2> + 3.5%|∓5/2> + 2.1%|∓7/2>	50.7	±2.8339
322.5197	224.1624	15.9893	0.7905	0.3845	22%|±11/2> + 20%|±13/2> + 11%|±5/2> + 9.2%|±1/2> + 8.0%|∓1/2> + 6.1%|±3/2> + 5.1%|∓3/2> + 4.7%|±7/2> + 3.5%|±15/2> + 2.5%|∓5/2> + 2.4%|±9/2> + 2.3%|∓7/2> + 2.1%|∓9/2>	48.1	±3.6243
347.0194	241.1906	11.5909	5.8412	1.5629	26%|±13/2> + 25%|±11/2> + 11%|±9/2> + 8.5%|±3/2> + 8.5%|±5/2> + 7.3%|±7/2> + 5.7%|±15/2> + 2.8%|∓9/2> + 2.3%|∓13/2>	76.5	±5.3453

[a] Contributions > 2%

Table S17. CASSCF-SO calculated states of the ground $^4I_{15/2}$ term for **2-Er·tol,** showing g-tensor components, ground state composition, angle between the ground state z axis and the excited states z axis, and expectation value of the J_z operator.

Energy / cm^{-1}	Energy / K	g_z	g_y [a]	g_x [a]	Composition [b]	g_z angle / °	$\langle J_z \rangle$
0.0000	0.0000	12.2613	0.2306	0.2120	29%|±15/2> + 29%|±9/2> + 20%|∓9/2> + 12%|∓15/2> + 7.6%|∓3/2> + 2.3%|±3/2>	-	±6.1307
8.8812	12.7781	3.7284	7.9677	8.4094	52%|±7/2> + 29%|∓5/2> + 9.7%|∓7/2> + 5.0%|±5/2> + 3.2%|±13/2>	0.2	±1.8642
86.3421	124.2270	10.3602	2.8228	0.6646	29%|±11/2> + 25%|∓1/2> + 17%|±15/2> + 6.8%|∓3/2> + 5.7%|±9/2> + 5.1%|±3/2> + 4.5%|±5/2>	54.3	±3.2481
86.7887	124.8696	1.6654	5.4629	8.8539	31%|±15/2> + 19%|∓3/2> + 13%|±11/2> + 10%|∓1/2> + 9.6%|±9/2> + 4.1%|∓11/2> + 4.1%|±1/2> + 3.5%|±3/2>	3.7	±3.7693
130.2453	187.3939	0.0867	7.3038	7.3316	39%|±5/2> + 15%|∓13/2> + 14%|∓7/2> + 11%|∓5/2> + 8.9%|±11/2> + 4.4%|±13/2> + 3.7%|±7/2> + 2.5%|∓11/2>	58.1	±0.0511
157.3901	226.4492	3.9295	3.7480	3.6621	50%|±13/2> + 33%|∓11/2> + 14%|±1/2> + 3.5%|±7/2>	9.9	±1.9606
189.6663	272.8875	4.8317	0.0473	0.0232	52%|±3/2> + 19%|∓9/2> + 16%|±9/2> + 6.6%|±15/2> + 3.4%|∓3/2> + 3.0%|∓15/2>	10.3	±2.4158
236.3974	340.1231	3.7079	8.0373	8.0564	40%|±1/2> + 21%|±13/2> + 13%|±7/2> + 8.3%|∓5/2> + 7.9%|∓11/2> + 4.1%|∓1/2> + 2.4%|∓13/2>	0.4	±1.8542

[a] By symmetry, g_x and g_y should be equivalent. Any deviation is because of rounding of coordinates, or failure of the S_{eff} = ½ model in the case of near-degenerate states. [b] Contributions > 2%.

Table S18. CASSCF-SO calculated states of the ground $^4I_{15/2}$ term for **3-Er·3thf,** showing g-tensor components, ground state composition, angle between the ground state z axis and the excited states z axis, and expectation value of the J_z operator.

<table>
<tr><th>Energy / cm^{-1}</th><th>Energy / K</th><th>g_z</th><th>g_y</th><th>g_x</th><th>Composition[a]</th><th>g_z angle / °</th><th>$\langle J_z \rangle$</th></tr>
<tr><td>0</td><td>0</td><td>14.0481</td><td>3.3265</td><td>1.3901</td><td>40%|±15/2> + 30%|∓15/2> + 7.7%|±9/2> + 3.6%|∓9/2> + 3.6%|±5/2> + 2.9%|±11/2> + 2.4%|±7/2></td><td>-</td><td>±7.0240</td></tr>
<tr><td>12.3814</td><td>17.8141</td><td>11.7047</td><td>2.6566</td><td>0.8500</td><td>16%|±3/2> + 13%|∓15/2> + 13%|±7/2> + 13%|±5/2> + 9.9%|∓1/2> + 6.2%|±13/2> + 5.9%|∓5/2> + 5.9%|∓3/2> + 5.7%|∓11/2> + 4.6%|±1/2></td><td>86.5</td><td>±0.5692</td></tr>
<tr><td>41.3593</td><td>59.50676</td><td>9.6373</td><td>5.2898</td><td>2.8866</td><td>44%|±13/2> + 12%|±13/2> + 9.3%|∓1/2> + 8.6%|∓11/2> 6.2%|±7/2> + 4.0%|±1/2> + 3.9%|±9/2> + 2.4%|±15/2> + 2.3%|∓5/2></td><td>21.3</td><td>±4.5473</td></tr>
<tr><td>78.0707</td><td>112.3263</td><td>9.4965</td><td>3.8096</td><td>0.4543</td><td>20%|±11/2> + 14%|∓5/2> + 14%|∓1/2> + 8.1%|∓11/2> + 8.0%|∓3/2> + 7.4%|±3/2> + 6.8%|±9/2> + 6.7%|∓13/2> + 5.7%|±7/2> + 2.3%|∓9/2> + 2.0%|±1/2></td><td>90.2</td><td>±1.8971</td></tr>
<tr><td>104.6899</td><td>150.6253</td><td>8.9925</td><td>4.2693</td><td>1.8389</td><td>20%|±9/2> + 11%|∓11/2> + 10%|±11/2> + 9.8%|∓7/2> + 8.9%|∓1/2> + 8.5%|∓3/2> + 7.9%|±5/2> + 7.8%|±3/2> + 5.9%|∓13/2> + 3.7%|±7/2> + 2.3%|∓15/2></td><td>81.2</td><td>±1.4071</td></tr>
<tr><td>117.6602</td><td>169.2867</td><td>9.7513</td><td>3.4171</td><td>0.7285</td><td>17%|±9/2> + 14%|∓7/2> + 13%||±1/2> + 12%|±11/2> + 11%|∓9/2> + 11%|±13/2> + 4.6%|±15/2> + 3.9%|∓11/2> + 3.4%|∓13/2> + 3.1%|∓5/2> + 2.5%|±7/2></td><td>75.4</td><td>±1.3705</td></tr>
<tr><td>144.2831</td><td>207.5911</td><td>13.2408</td><td>2.2485</td><td>1.6207</td><td>17%|±7/2> + 14%|∓5/2> + 13%|∓9/2> + 12%|∓11/2> + 11%|±3/2> + 8.8%|±5/2> + 5.3%|±13/2> + 5.2%|±1/2> + 4.7%|∓7/2> + 3.6%|∓3/2> + 3.1%|∓15/2></td><td>80.9</td><td>±1.3903</td></tr>
<tr><td>232.1320</td><td>333.9861</td><td>17.6634</td><td>0.0639</td><td>0.0331</td><td>19%|±1/2> + 19%|±5/2> + 14%|∓3/2> + 11%|±3/2> + 8.5%|∓7/2> + 7.6%|±7/2> + 7.1%|±9/2> + 6.5%|∓1/2> + 4.0%|∓5/2></td><td>84.5</td><td>±0.8394</td></tr>
</table>

[a] Contributions > 2%

Table S19. CASSCF-SO calculated states of the ground $^{4}I_{15/2}$ term for **3-Er·dcm**, showing g-tensor components, ground state composition, angle between the ground state z axis and the excited states z axis, and expectation value of the J_z operator.

Energy / cm^{-1}	Energy / K	g_z	g_y	g_x	Composition[a]	g_z angle / °	$\langle J_z \rangle$
0	0	13.6645	3.3581	0.2023	51%|±15/2> + 24%|±11/2> + 5.0%|±7/2> + 4.0%|±13/2> + 2.8%|∓9/2> + 2.5%|∓15/2> + 2.1%|±5/2>	-	±6.8322
16.0510	23.0938	9.5589	3.9237	1.0082	22%|±9/2> + 19%|±13/2> + 16%|±5/2> + 11%|±7/2> + 6.7%|∓7/2> + 6.6%|∓15/2> + 5.7%|∓9/2> + 3.8%|∓3/2> + 2.2%|∓5/2>	48.3	±3.3224
32.7040	47.0538	7.4759	3.8247	1.0626	17%|±13/2> + 14%|∓13/2> + 11%|±3/2> + 7.9%|±5/2> + 7.6%|∓11/2> + 7.2%|±11/2> + 7.0%|±7/2> + 6.3%|±15/2> + 5.4%|±9/2> + 5.2%|∓3/2> + 4.5%|∓7/2> + 4.4%|∓5/2>	66.5	±3.4693
56.9070	81.8765	12.0570	2.6256	1.4181	34%|±1/2> + 12%|∓3/2> + 8.9%|±11/2> + 7.8%|∓7/2> + 7.8%|±9/2> + 6.6%|∓9/2> + 4.7%|±3/2> + 4.0%|±7/2> + 3.7%|∓5/2> + 3.6%|∓13/2> + 3.0%|∓11/2> + 3.0%|±15/2>	61.1	±1.4304
113.3200	163.0422	9.9889	4.2851	1.8512	15%|±15/2> + 14%|∓1/2> + 14%|±11/2> + 13%|±13/2> + 9.8%|∓7/2> + 6.7%|±5/2> 6.7%|±9/2> + 6.0%|∓9/2> + 4.9%|±7/2> + 3.2%|±1/2> + 2.8%|∓11/2>	79.8	±3.1338
125.8743	181.1050	8.2362	4.9701	1.9177	25%|±3/2> + 16%|∓5/2> + 14%|±5/2> + 7.8%|∓13/2> + 7.5%|±7/2> + 6.9%|∓7/2> + 6.4%|∓15/2> + 3.5%|∓9/2> + 3.3%|±13/2> + 2.6%|∓3/2> + 2.5%|∓11/2>	50.8	±1.0266
149.1800	214.6367	12.3488	4.0945	1.4973	22%|±1/2> + 20%|∓3/2> + 9.9%|±11/2> + 9.1%|∓1/2> + 7.3%|±13/2> + 5.7%|±9/2> + 5.3%|±15/2> + 5.0%|∓5/2> + 4.5%|∓9/2> + 3.1%|±5/2> + 3.0%|∓11/2> + 2.4%|±7/2>	19.4	±1.5910
219.5057	315.8197	17.5690	0.0579	0.0308	19%|±7/2> + 19%|±9/2> + 15%|±5/2> + 14%|±11/2> + 7.8%|±3/2> + 6.6%|±1/2> + 5.9%|±13/2> + 4.9%|∓3/2> + 3.9%|∓1/2>	85.2	±3.7259

[a] Contributions > 2%

Table S20. CASSCF-SO calculated states of the ground $^6H_{15/2}$ term for **1-Dy·0.5tol**, showing g-tensor components, ground state composition, angle between the ground state z axis and the excited states z axis, and expectation value of the J_z operator.

<table>
<tr><th>Energy / cm^{-1}</th><th>Energy / K</th><th>g_z</th><th>g_y</th><th>g_x</th><th>Composition[a]</th><th>g_z angle / °</th><th><J_z></th></tr>
<tr><td>0</td><td>0</td><td>17.1585</td><td>1.3073</td><td>0.8168</td><td>80%|±15/2> + 4.1%|±1/2> + 3.1%|±7/2> + 3.1%|±9/2> + 2.1%|∓1/2> + 2.1%|±13/2></td><td>-</td><td>±8.5792</td></tr>
<tr><td>47.0356</td><td>67.6737</td><td>15.2932</td><td>2.9132</td><td>0.7475</td><td>29%|±1/2> + 15%|∓3/2> + 14%|±3/2> + 11%|∓1/2> + 10%|±5/2> + 5.9%|±15/2> + 3.2%|∓15/2> + 3.1%|∓7/2> + 2.5%|±7/2> + 2.2%|±11/2></td><td>84.1</td><td>±0.9403</td></tr>
<tr><td>66.5257</td><td>95.7156</td><td>14.0044</td><td>1.6556</td><td>0.5069</td><td>22%|±1/2> + 19%|∓3/2> + 13%|∓5/2> + 10%|±5/2> + 8.1%|∓13/2> + 6.6%|∓7/2> + 3.7%|∓1/2> + 3.7%|±13/2> + 3.4%|∓15/2> + 2.6%|∓11/2> + 2.6%|±9/2> + 2.4%|±3/2></td><td>71.5</td><td>±2.2483</td></tr>
<tr><td>178.0331</td><td>256.1499</td><td>5.8912</td><td>7.3099</td><td>8.5991</td><td>44%|±13/2> + 11%|∓3/2> + 8.7%|∓1/2> + 7.5%|±3/2> + 5.3%|±5/2> + 5.2%|∓11/2> + 5.1%|±7/2> + 3.9%|±1/2> + 3.8%|±11/2></td><td>73.7</td><td>±4.0769</td></tr>
<tr><td>325.8672</td><td>468.8502</td><td>2.0730</td><td>6.4179</td><td>8.9077</td><td>22%|±13/2> + 20%|±5/2> + 11%|∓7/2> + 9.5%|∓11/2> + 8.1%|±7/2> + 7.4%|∓13/2> + 4.4%|∓9/2> + 4.2%|±9/2> + 4.1%|∓5/2> + 3.5%|±11/2> + 2.4%|±3/2></td><td>53.4</td><td>±3.3824</td></tr>
<tr><td>350.7528</td><td>504.6550</td><td>13.4522</td><td>4.1655</td><td>0.3101</td><td>32%|±9/2> + 20%|±11/2> + 17%|±7/2> + 4.9%|∓5/2> + 4.0%|±15/2> + 3.6%|±13/2> + 3.3%|∓13/2> + 2.9%|±1/2> + 2.7%|∓9/2> + 2.4%|±5/2> + 2.0%|∓11/2></td><td>45.6</td><td>±4.7126</td></tr>
<tr><td>406.5054</td><td>584.8706</td><td>11.6617</td><td>4.9455</td><td>1.8672</td><td>36%|±11/2> + 18%|±3/2> + 11%|±7/2> + 10%|∓5/2> + 6.4%|±5/2> + 3.1%|±9/2> + 2.9%|∓3/2> + 2.7%|∓11/2> + 2.7%|±1/2> + 2.1%|∓1/2></td><td>61.8</td><td>±3.4899</td></tr>
<tr><td>478.4977</td><td>688.4514</td><td>16.9308</td><td>0.6512</td><td>0.2131</td><td>34%|±9/2> + 30%|±7/2> + 10%|±5/2> + 9.1%|±11/2> + 6.8%|∓9/2> + 4.7%|∓1/2></td><td>56.4</td><td>±4.6923</td></tr>
</table>

[a] Contributions > 2%

Table S21. CASSCF-SO calculated states of the ground $^6H_{15/2}$ term for **2-Dy·tol**, showing g-tensor components, ground state composition, angle between the ground state z axis and the excited states z axis, and expectation value of the J_z operator.

Energy / cm^{-1}	Energy / K	g_z	g_y[a]	g_x[a]	Composition[b]	g_z angle / °	$\langle J_z \rangle$
0	0	3.0459	9.1382	9.4474	60%|±7/2> + 39%|∓5/2>	0.8	±1.5229
11.2125	16.1323	7.1304	0.1644	0.1129	56%|±9/2> + 20%|∓3/2> + 18%|±3/2> + 3.5%|±15/2> + 2.3%|∓9/2>	0.9	±3.5649
33.7532	48.5633	2.0627	8.7944	8.8441	31%|±1/2> + 20%|∓1/2> + 15%|∓5/2> + 9.5%|±5/2> + 8.9%|∓11/2> + 5.8%|±7/2> + 5.7%|±11/2> + 3.8%|∓7/2>	0.3	±1.0314
78.7668	113.3279	9.4242	0.1059	0.0983	41%|±15/2> + 26%|±3/2> + 18%|∓3/2> + 12%|±9/2> + 3.3%|∓9/2>	0.2	±4.7121
89.6790	129.0281	4.0298	7.7438	7.8871	32%|±11/2> + 31%|±5/2> + 20%|∓7/2> + 6.0%|∓1/2> + 5.7%|∓13/2>	4.0	±2.0149
110.2221	158.5850	2.7419	1.5004	1.3895	38%|±11/2> + 19%|∓1/2> + 17%|∓13/2> + 10%|∓11/2> + 5.4%|±1/2> + 5.0%|±13/2> + 2.3%|∓7/2>	3.8	±1.3685
123.3721	177.5049	14.1947	0.0783	0.0567	43%|±15/2> + 18%|±9/2> + 17%|±3/2> + 12%|∓15/2> + 9.2%|∓9/2>	1.2	±7.0973
147.4555	212.1556	12.3763	4.6872	4.6465	71%|±13/2> + 17%|±1/2> + 6.9%|±7/2> + 3.2%|∓11/2> + 2.4%|∓5/2>	0.8	±6.1874

[a] By symmetry, g_x and g_y should be equivalent; any deviation is because of rounding of coordinates. [b] Contributions > 2%

Table S22. CASSCF-SO calculated states of the ground $^6H_{15/2}$ term for **3-Dy·2thf**, showing g-tensor components, ground state composition, angle between the ground state z axis and the excited states z axis, and expectation value of the J_z operator.

<table>
<tr><th>Energy / cm^{-1}</th><th>Energy / K</th><th>g_z</th><th>g_y</th><th>g_x</th><th>Composition[a]</th><th>g_z angle / °</th><th><J_z></th></tr>
<tr><td>0</td><td>0</td><td>19.6800</td><td>0.0433</td><td>0.0245</td><td>94%|±15/2> + 3.0%|∓15/2> + 2.9%|±11/2></td><td>-</td><td>±9.8400</td></tr>
<tr><td>120.4276</td><td>173.2684</td><td>16.8004</td><td>0.3670</td><td>0.2882</td><td>85%|±13/2> + 10%|∓13/2> + 2.9%|±9/2></td><td>0.6</td><td>±8.3997</td></tr>
<tr><td>233.0315</td><td>335.2803</td><td>12.0201</td><td>4.3121</td><td>2.8254</td><td>76%|±11/2> + 8.0%|±3/2> + 4.9%|±7/2> + 4.7%|∓1/2> + 3.0%|±15/2></td><td>5.0</td><td>±5.9859</td></tr>
<tr><td>285.6587</td><td>410.9991</td><td>1.8434</td><td>7.1741</td><td>9.3745</td><td>33%|±1/2> + 29%|±9/2> + 14%|∓11/2> + 6.3%|±5/2> + 6.1%|∓3/2> + 5.9%|∓7/2> + 2.8%|±13/2></td><td>16.6</td><td>±1.1468</td></tr>
<tr><td>323.2504</td><td>465.0852</td><td>12.8490</td><td>5.2457</td><td>1.0630</td><td>38%|±3/2> + 18%|∓5/2> + 12%|∓1/2> + 10%|±9/2> + 9.2%|±5/2> + 4.4%|±7/2> + 2.3%|±11/2> + 2.2%|∓9/2> + 2.1%|∓3/2></td><td>85.7</td><td>±1.3631</td></tr>
<tr><td>335.4307</td><td>482.6099</td><td>1.6174</td><td>5.5489</td><td>7.7177</td><td>25%|±9/2> + 20%|±7/2> + 14%|∓9/2> + 10%|±1/2> + 8.6%|∓7/2> + 7.7%|∓5/2> + 6.2%|±5/2> + 3.0%|±3/2> + 2.4%|∓3/2></td><td>76.1</td><td>±1.4191</td></tr>
<tr><td>368.7191</td><td>530.5045</td><td>17.6114</td><td>1.8641</td><td>1.2371</td><td>36%|±7/2> + 16%|∓5/2> + 11%|±3/2> + 10%|±5/2> + 5.8%|±1/2> + 5.2%|∓9/2> + 5.2%|±9/2> + 4.8%|∓7/2> + 2.9%|∓1/2></td><td>49.9</td><td>±2.9056</td></tr>
<tr><td>460.7798</td><td>662.9593</td><td>19.7066</td><td>0.0697</td><td>0.0410</td><td>20%|±3/2> + 16%|∓1/2> + 14%|±5/2> + 12%|±1/2> + 12%|±7/2> + 11%|∓5/2> + 8.1%|∓3/2> + 2.4%|±9/2> + 2.3%|∓7/2></td><td>84.5</td><td>±0.9939</td></tr>
</table>

[a] Contributions > 2%

Table S23. CASSCF-SO calculated states of the ground $^6H_{15/2}$ term for **3-Dy·dcm**, showing g-tensor components, ground state composition, angle between the ground state z axis and the excited states z axis, and expectation value of the J_z operator.

<table>
<tr><th>Energy / cm^{-1}</th><th>Energy / K</th><th>g_z</th><th>g_y</th><th>g_x</th><th>Composition[a]</th><th>g_z angle / °</th><th><J_z></th></tr>
<tr><td>0</td><td>0</td><td>19.4810</td><td>0.1388</td><td>0.0671</td><td>71%|±15/2> + 23%|∓15/2> + 4.0%|±11/2></td><td>4.9</td><td>±9.7405</td></tr>
<tr><td>88.1370</td><td>126.8095</td><td>16.1900</td><td>1.0265</td><td>0.6812</td><td>53%|±13/2> + 35%|∓13/2> + 3.5%|±9/2> + 2.4%|∓9/2></td><td>85.6</td><td>±8.0655</td></tr>
<tr><td>161.0938</td><td>231.7780</td><td>13.1515</td><td>3.8946</td><td>3.3194</td><td>23%|±11/2> + 23%|±3/2> + 18%|∓1/2> + 9.5%|±7/2> + 9.5%|∓5/2> + 4.3%|∓13/2> + 3.5%|±1/2> + 2.9%|±15/2> + 2.2%|∓9/2></td><td>75.3</td><td>±1.9813</td></tr>
<tr><td>193.3549</td><td>278.1946</td><td>11.4850</td><td>5.8231</td><td>0.9909</td><td>33%|±11/2> + 20%|±1/2> + 13%|±5/2> + 9.4%|∓5/2> + 8.5%|∓3/2> + 4.6%|±7/2> + 4.5%|±3/2></td><td>71.9</td><td>±3.1696</td></tr>
<tr><td>209.6967</td><td>301.7068</td><td>11.9997</td><td>1.9952</td><td>1.3962</td><td>25%|±11/2> + 14%|±1/2> + 13%|±9/2> + 11%|±7/2> + 10%|±3/2> + 5.4%|±9/2> + 4.8%|±5/2> + 4.2%|±5/2> + 3.9%|±1/2> + 3.2%|±7/2></td><td>71.1</td><td>±1.9766</td></tr>
<tr><td>236.8244</td><td>340.7375</td><td>14.4534</td><td>4.4085</td><td>2.8387</td><td>26%|±9/2> + 18%|∓9/2> + 11%|±3/2> + 9.9%|±7/2> + 9.8%|∓7/2> + 9.3%|∓1/2> + 7.3%|±5/2> + 3.7%|±11/2></td><td>66.8</td><td>±2.9122</td></tr>
<tr><td>303.4693</td><td>436.6246</td><td>19.0536</td><td>0.4628</td><td>0.2871</td><td>23%|±7/2> + 17%|±5/2> + 16%|±3/2> + 13%|±9/2> + 7.5%|±1/2> + 5.9%|∓5/2> + 5.7%|∓9/2> + 4.0%|±11/2> + 3.8%|∓7/2></td><td>73.8</td><td>±3.7513</td></tr>
<tr><td>363.8244</td><td>523.4621</td><td>19.6542</td><td>0.1540</td><td>0.0769</td><td>23%|±5/2> + 22%|±7/2> + 18%|±3/2> + 9.8%|±1/2> + 9.3%|±9/2> + 8.3%|∓1/2> + 3.5%|∓3/2> + 2.9%|∓5/2> + 2.3%|±11/2></td><td>4.9</td><td>±2.7366</td></tr>
</table>

[a] Contributions > 2%

Table S24. CF parameters (B_k^q, k = 2, 4, 6) used to parametrize **1-3-Er** CASSCF results

k	Q	K^2	**1-Er·0.5tol**	**2-Er·tol**	**3-Er·3thf**	**3-Er·dcm**
2	-2	1.50	0.22404296442973	0.69698477648214E-3 [a]	-0.52115118272978	0.62447397254434
2	-1	6.00	0.68072116745680	-0.31568160360124E-4 [a]	-0.41492672279244	0.19474157265577
2	0	1.00	-0.56956147356845E-3	0.12742949385054	-0.38179270497316	-0.53891423222019
2	+1	6.00	0.70301194814302E-1	-0.24690168179385E-4 [a]	0.80737157148785	-0.21562240593298
2	+2	1.50	-0.19859971872516	-0.37812982077973 [a]	0.45861595000017	0.77007250679198
4	-4	17.50	0.86455428851152E-2	-0.24437336298372E-2 [a]	-0.49661775450072E-3	0.86370345885514E-2
4	-3	140.00	0.36752409930150E-1	0.19697264947715E-5	-0.32986426896039E-1	-0.40958534442039E-2
4	-2	10.00	0.38703227253562E-1	-0.48824622096569E-2 [a]	0.29408576294775E-4	-0.65390953472310E-2
4	-1	20.00	-0.13427775858990E-1	-0.66868163148094E-6 [a]	-0.93338447236652E-2	-0.96635896573089E-2
4	0	1.00	-0.27845722047821E-2	-0.57666700985447E-5	-0.33004080485319E-3	-0.10275433687236E-2
4	+1	20.00	0.57350459039864E-2	0.36450424595428E-5 [a]	-0.14464907753008E-1	0.68378137307235E-2
4	+2	10.00	0.20453321579561E-2	0.44040631606812E-4 [a]	0.66211287111418E-2	-0.67476006190386E-2
4	+3	140.00	0.39890770238934E-1	0.96551466753245E-5	-0.21887027490003E-2	-0.16878619512180E-1
4	+4	17.50	-0.29102933813311E-1	0.59986258416766E-4 [a]	-0.52333321420683E-2	-0.83567521910456E-3
6	-6	57.75	0.24539740019061E-3	-0.11946231384123E-2	0.31671388144367E-4	0.24641238330353E-3
6	-5	693.00	-0.50656561558630E-3	0.91842126743188E-6 [a]	-0.20750364149669E-3	-0.10972471923271E-3
6	-4	31.50	-0.37340892338152E-4	-0.13609928482823E-2 [a]	-0.20579721268080E-4	-0.50802407645496E-4
6	-3	105.00	0.12990583961412E-3	0.12145506900857E-5	-0.11113641860566E-3	0.15150577032952E-3
6	-2	26.25	0.14075989280810E-4	0.98221142224771E-3 [a]	0.18314261440761E-3	-0.53102149181804E-4
6	-1	42.00	0.16134253303584E-3	0.18549840271772E-5 [a]	0.10983604962320E-3	0.10910190718777E-3
6	0	1.00	-0.31340977173268E-4	0.26903245787503E-3	-0.31135272042918E-5	0.96459579498531E-7
6	+1	42.00	-0.13746772616421E-3	-0.59686357857411E-6 [a]	-0.43812404154700E-4	-0.11155227058094E-3
6	+2	26.25	-0.13779612159847E-4	0.83395387961000E-3 [a]	0.11037066303951E-4	-0.12063431558538E-4
6	+3	105.00	0.48680551504270E-4	0.16004005731895E-6	-0.14374479484278E-3	-0.26141272770184E-3
6	+4	31.50	-0.34388666144939E-3	0.14193696228769E-2 [a]	-0.30593452668672E-4	-0.46272948493572E-4
6	+5	693.00	-0.64961138781068E-3	0.23067112147559E-5 [a]	-0.10665922726764E-2	0.40082176425455E-3
6	+6	57.75	0.23019036165826E-3	-0.14432596566566E-2	-0.26087488232781E-3	-0.22046182434924E-3

[a] By symmetry should be zero; deviations are due to rounding errors in the atomic positions and fitting of the ab-initio states to a crystal field model.

Table S25. CF parameters (B_k^q, k = 2, 4, 6) used to parametrize **1-3-Dy** CASSCF results

k	*Q*	K^2	1-Dy·0.5tol	2-Dy·tol	3-Dy·2thf	3-Dy·dcm
2	-2	1.50	-0.32166167810231	0.26653601967089E-4 [a]	0.62394828773325	0.53629434092353
2	-1	6.00	-0.92552346892350	0.77300354105921E-2 [a]	0.14555619878149	-0.17700667201649
2	0	1.00	-0.50402341341244	0.39845228107106	-2.0991759710180	-1.3867632755198
2	+1	6.00	1.6693844707014	0.77137911603230E-4 [a]	0.50946904312292	-1.1982507539076
2	+2	1.50	0.16940143123524	-0.34100165803723E-3 [a]	0.58476861721814	0.60023916651736
4	-4	17.50	0.50254734537043E-1	0.27684759075217E-7 [a]	0.13767366839933E-2	0.20422131655876E-2
4	-3	140.00	-0.44493364465922E-1	0.12189794861405E-1	-0.26264449061963E-2	0.21762565284838E-2
4	-2	10.00	0.53730020521474E-2	-0.85657641090610E-6 [a]	-0.62878379109530E-2	-0.38102816612017E-2
4	-1	20.00	0.28004583760905E-1	0.20499775464309E-4 [a]	-0.17671348340497E-2	0.76978002250383E-3
4	0	1.00	-0.10250626692419E-1	0.25509906127787E-3	-0.30199624320337E-2	-0.30008910333163E-2
4	+1	20.00	-0.20739514374107E-1	0.17728810452468E-6 [a]	-0.50634581078808E-2	0.14227638412574E-1
4	+2	10.00	-0.23222175219430E-2	-0.45986766479187E-4 [a]	0.83995565527961E-2	0.77780678053882E-2
4	+3	140.00	0.20332292071781E-1	-0.12848146422113E-3	-0.65010311086703E-2	0.29120715768314E-1
4	+4	17.50	0.25576390788297E-1	-0.16223788273215E-4 [a]	-0.22164996192860E-1	-0.21322704127936E-1
6	-6	57.75	0.61595070517519E-4	0.67931722100569E-5	-0.10342541355149E-3	-0.53593426963147E-4
6	-5	693.00	0.26463695417158E-3	-0.64242315074214E-5 [a]	-0.36709846158033E-4	0.12413626535212E-3
6	-4	31.50	0.19884347010937E-3	-0.27840293542603E-7 [a]	-0.73964330021599E-5	-0.98895559854292E-5
6	-3	105.00	-0.21722944664349E-3	-0.37669726781536E-3	-0.15923100557304E-4	0.81112340627495E-4
6	-2	26.25	0.66889280885016E-4	0.26119821788274E-7 [a]	0.72320582282956E-4	0.30133927214933E-4
6	-1	42.00	-0.88557614366491E-4	0.52827227713914E-7 [a]	0.10151314855581E-4	0.39091872091752E-4
6	0	1.00	0.19905935803469E-4	-0.22094306659553E-4	0.13146304614497E-4	0.13211323442474E-4
6	+1	42.00	0.38603899453028E-5	-0.40855387663750E-7 [a]	0.36881045194708E-4	-0.10585131420700E-3
6	+2	26.25	-0.10476194683722E-3	0.27755608954543E-6 [a]	0.87016979917862E-4	0.10145737831143E-3
6	+3	105.00	0.91541000552280E-4	0.38527957190432E-5	0.53541786472439E-4	-0.80283536685399E-4
6	+4	31.50	0.13744285726015E-3	-0.20286019359384E-6 [a]	-0.12948777498993E-4	-0.38227351536006E-4
6	+5	693.00	0.47353915689186E-3	0.29730791788118E-7 [a]	-0.88314358835290E-4	0.27897588966946E-3
6	+6	57.75	0.41979585741439E-4	0.33407724075017E-3	0.10403503885124E-3	0.91266496592272E-4

[a] By symmetry should be zero; deviations are due to rounding errors in the atomic positions and fitting of the ab-initio states to a crystal field model.

6. Cantilever Torque Magnetometry

In the initial torque model, the two Er^{III} ground doublets were described as an effective spin ½ with rhombic g values [g_x, g_y, g_z] fixed to the CASSCF values. The orientations of the g-tensors in the molecular frame (ab′c*) are defined by Euler angles (α, β, γ) and (360–α, β, 180–γ), using the EasySpin convention of active intrinsic rotations z-y′-z″. In the second model, the CASSCF crystal field parameters (B_k^q, k = 2, 4, 6, Table S24) were used to define each Er^{III} centre, with the Euler angles of the CF parameters kept the same as the S_{eff} = ½ model.

Under a given magnetic field vector, the Hamiltonian of the system was constructed using the EasySpin function *ham* and diagonalised, which was used to calculate the free energy of the system assuming a Boltzmann population at the measured temperature. The torque was calculated as the negative partial derivative of the free energy with respect to θ in radians, calculated using the finite difference method with Δθ = 0.001° according to equation 1:

$$\tau = -\frac{\delta F}{\delta \theta} \approx \frac{F(\theta + \Delta\theta) - F(\theta - \Delta\theta)}{2\Delta\theta}$$

The torque was fitted in the fitting program *esfit*, using the Nelder-Mead simplex algorithm and a customized function with each dataset normalised to the range of torque values.

Table S26. Initial orientations for torque measurements

Orientation	Rotation axis			Magnetic field at θ = 0°		
	a	b′	c*	a	b′	c*
Rotation 1	-0.59379	-0.06419	-0.80206	-0.45753	-0.79304	0.40219
Rotation 2	-0.66188	0.60578	0.44153	-0.45753	-0.79304	0.40219
Rotation 3	-0.45753	-0.79304	0.40219	0.59379	0.06419	0.80206

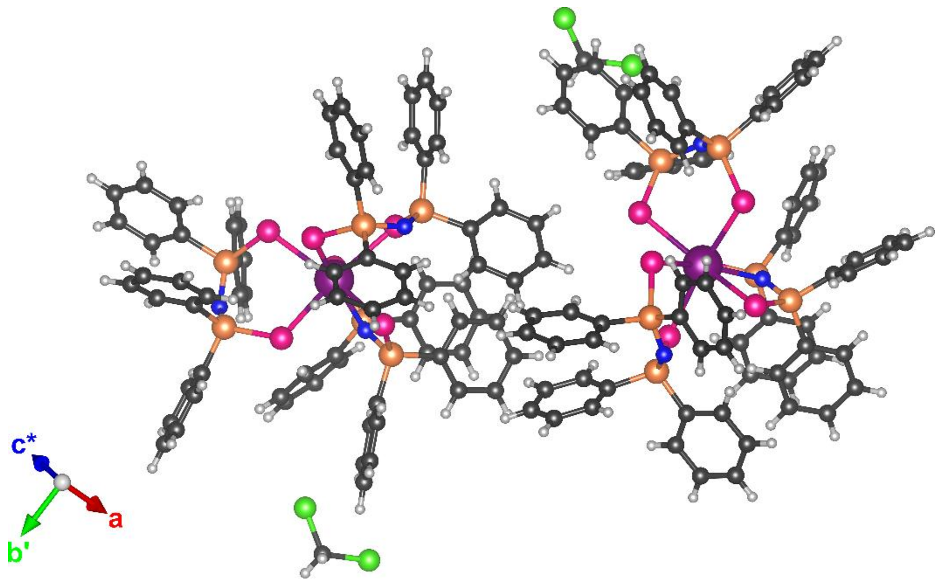

Figure S42. Orientation 1 of the **3-Er·dcm** crystal for torque magnetometry in the orthogonal ab′c* coordinate frame; the two magnetically unique molecules are shown. Color code: Er (purple), N (blue), P (peach), Se (pink), C (dark grey), H (light grey), Cl (green).

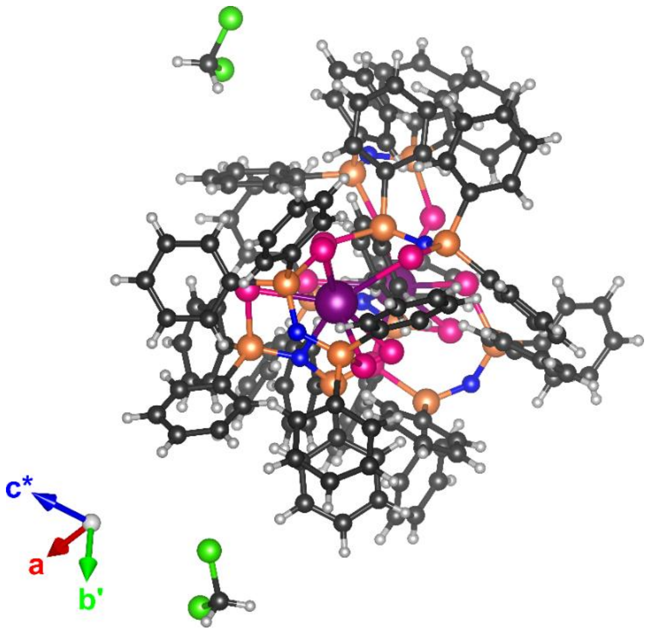

Figure S43. Orientation 2 of the **3-Er·dcm** crystal for torque magnetometry in the orthogonal ab′c* coordinate frame; the two magnetically unique molecules are shown. Color code: Er (purple), N (blue), P (peach), Se (pink), C (dark grey), H (light grey), Cl (green).

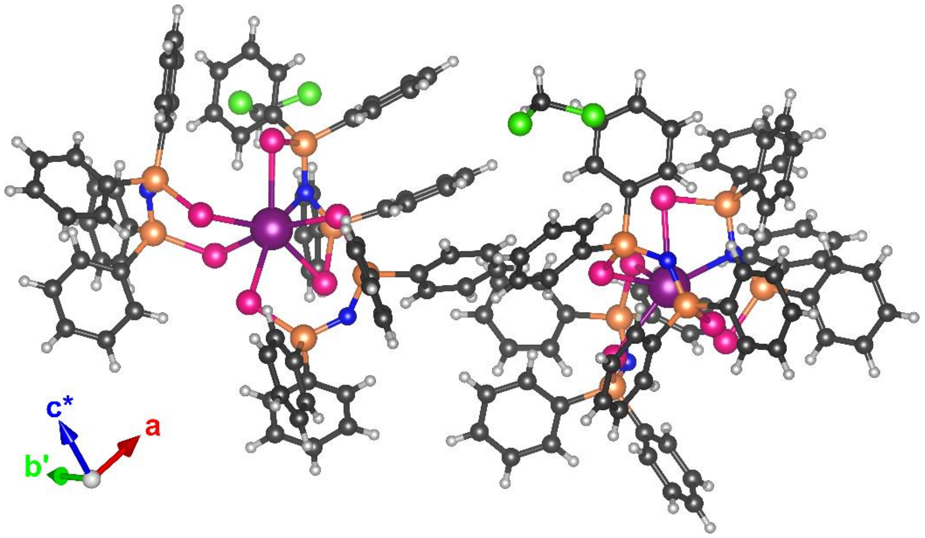

Figure S44. Orientation 3 of the **3-Er·dcm** crystal for torque magnetometry in the orthogonal ab′c* coordinate frame; the two magnetically unique molecules are shown. Color code: Er (purple), N (blue), P (peach), Se (pink), C (dark grey), H (light grey), Cl (green).

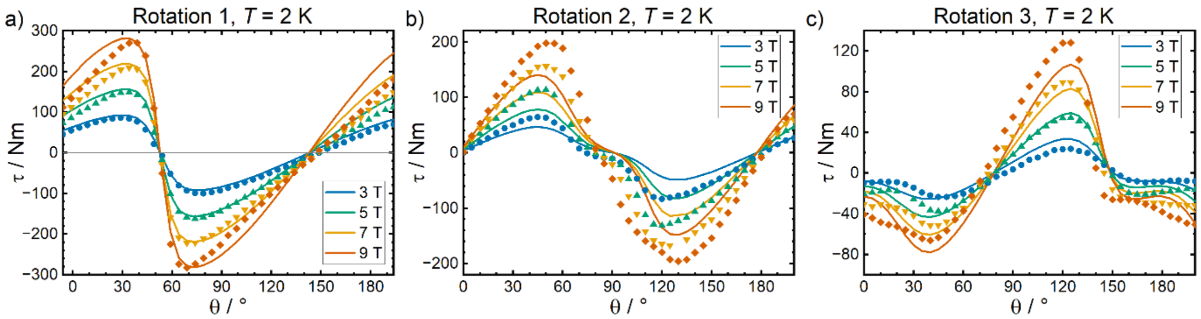

Figure S45. Torque curves for **3-Er·dcm** at 2 K for rotation axis 1 (a), 2 (b) and 3 (c). Simulation using S_{eff} = ½ model with g-values fixed to CASSCF values.

.

Table S27. Parameters used in $S_{eff} = ½$ simulation of **3-Er·dcm** torque.

	**Molecule 1**	**Molecule 2**
g_1	0.20231478	0.20231478
g_2	3.35806439	3.35806439
g_3	13.66449928	13.66449928
**α / °**	258.2(5)	108.8(5)
**β / °**	105.2(5)	105.2(5)
**γ / °**	79(2)	101(2)
**mass / µg**	1.96(3)	

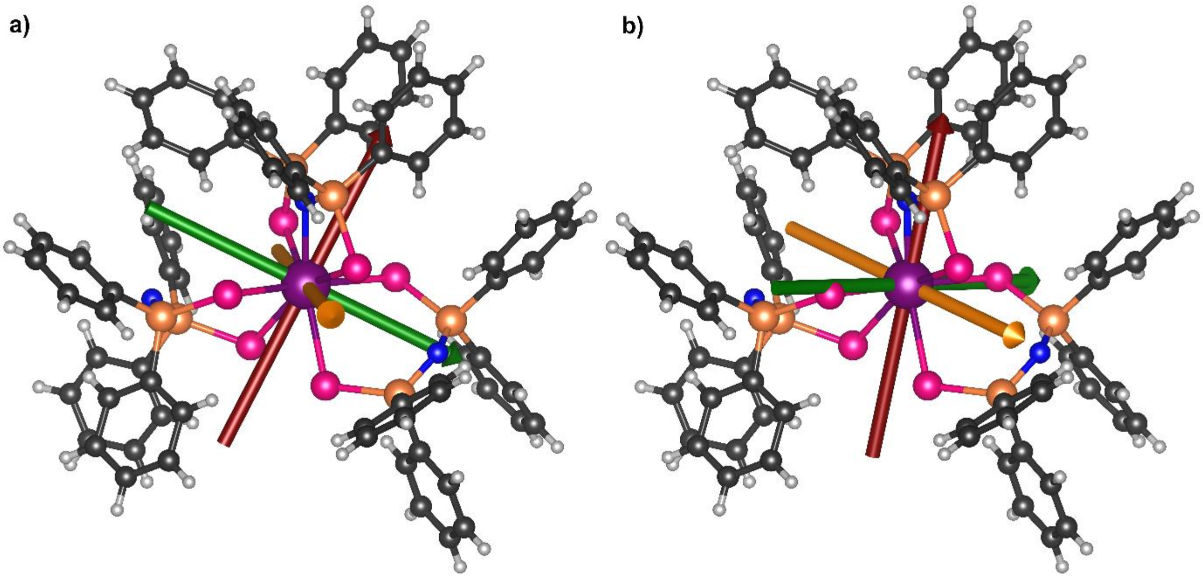

Figure S46. The two possible solutions for the *g*-tensor orientations in **3-Er·dcm**.

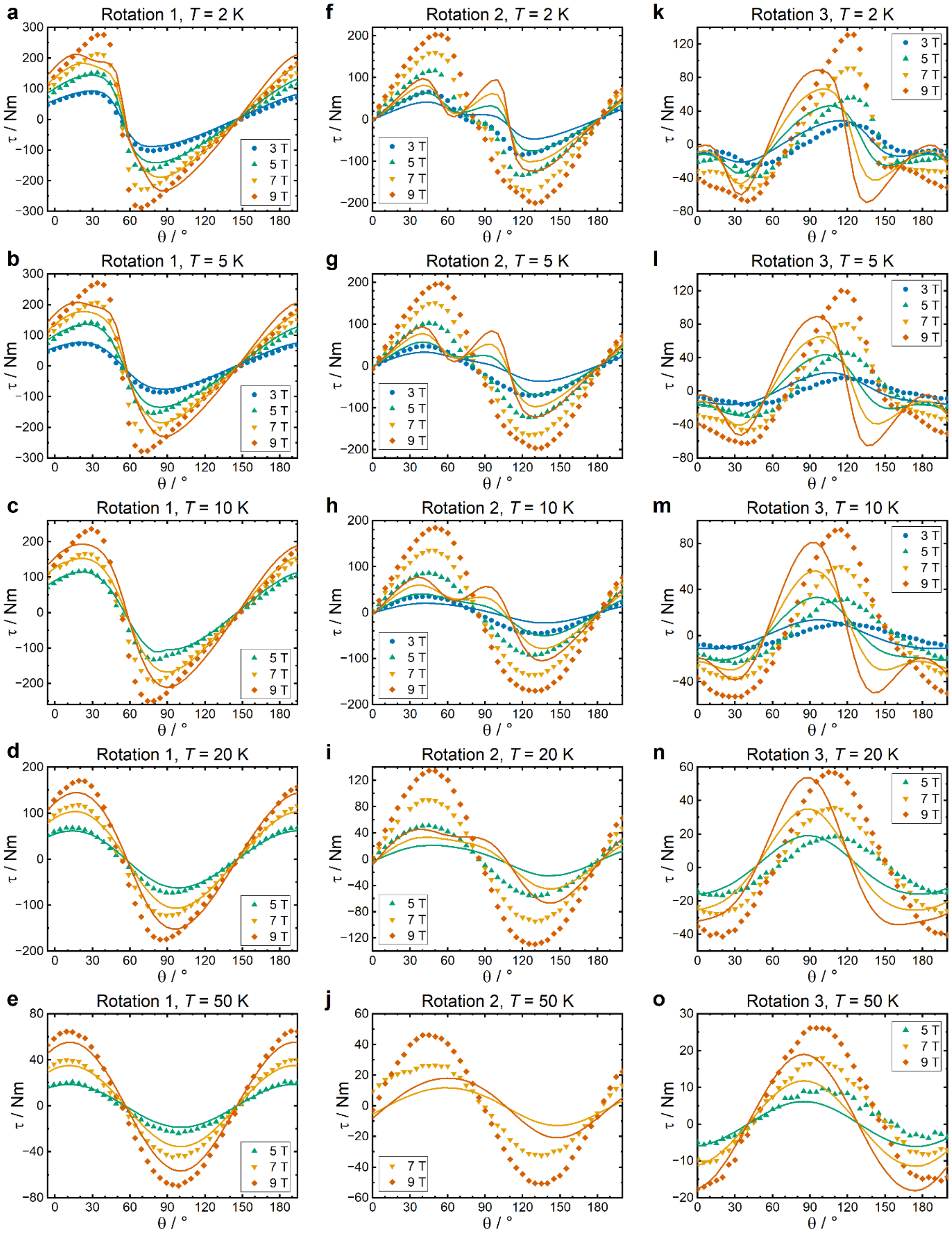

Figure S47. Simulated torque data for **3-Er·dcm**, modeled with full CASSCF crystal field and Euler angles fixed from $S_{\mathrm{eff}} = ½$ model.

7. AC Magnetometry

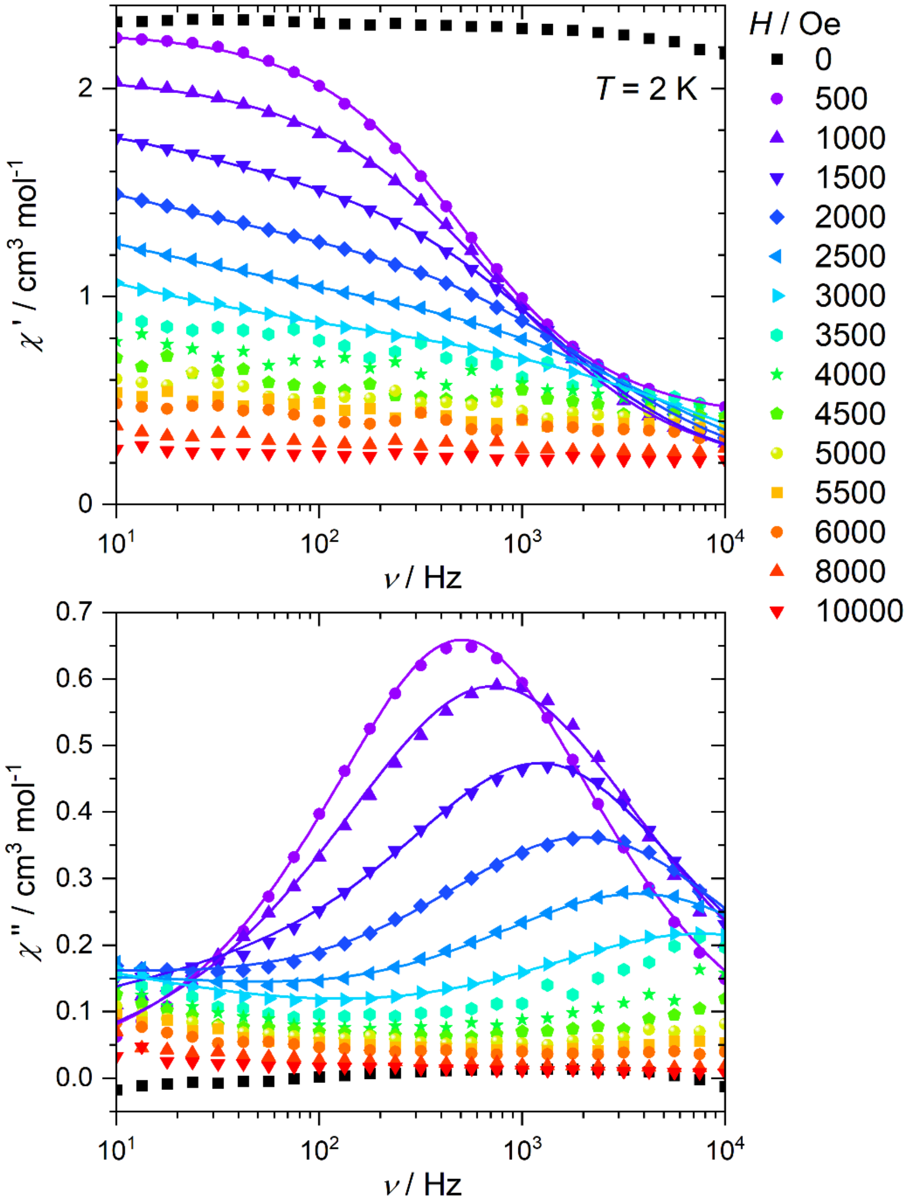

Figure S48. (a) In-phase (χ′) and **(b)** out-of-phase (χ″) ac susceptibilities for **1-Er·0.5tol** at 2 K and fields of 0–10000 Oe. Lines show fits to the Generalized Debye model (500, 1000 Oe) and Double Generalized Debye model (1500–3000 Oe).

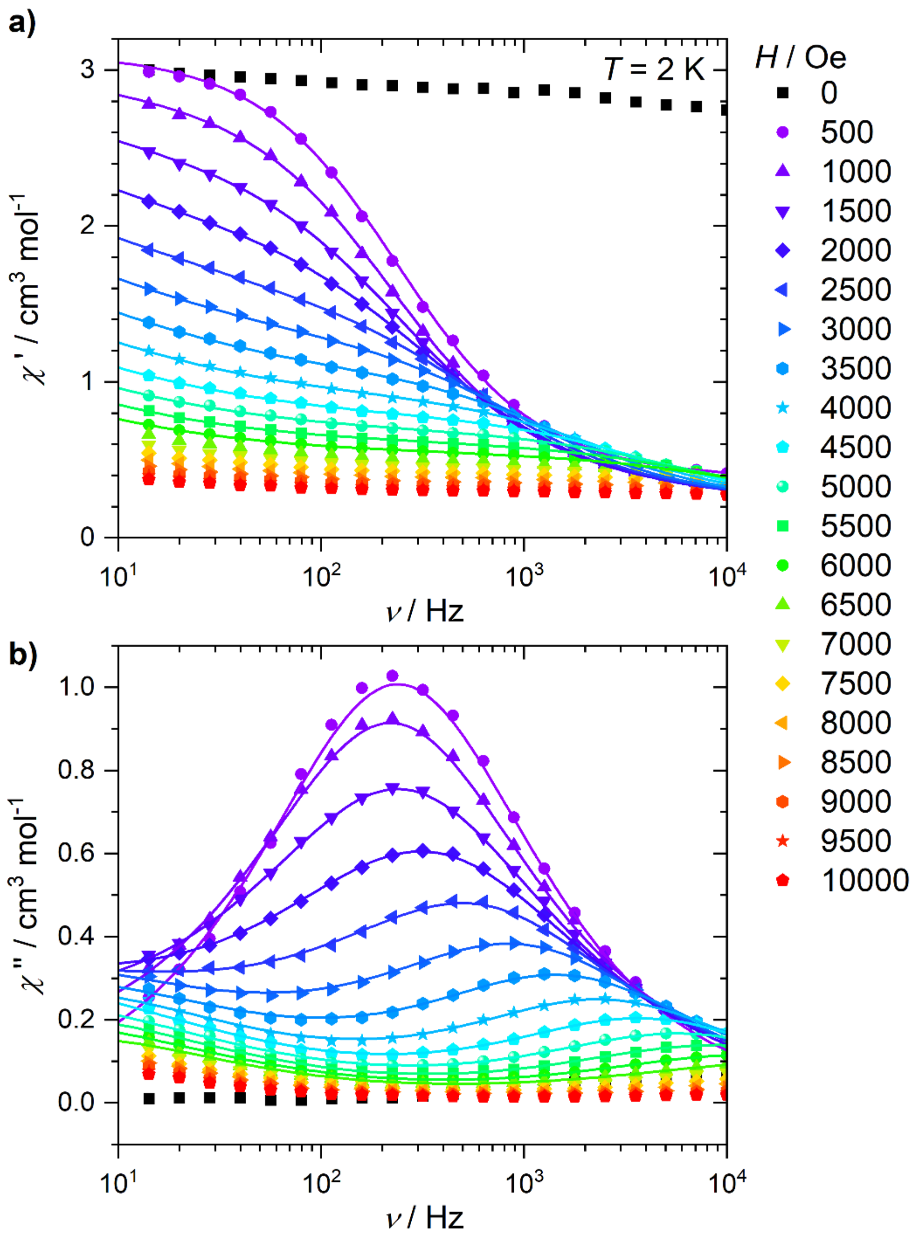

Figure S49. (a) In-phase (χ′) and **(b)** out-of-phase (χ″) ac susceptibilities for **2-Er·tol** at 2 K and fields of 0–10000 Oe. Lines show fits to the Generalized or Double Generalized Debye model.

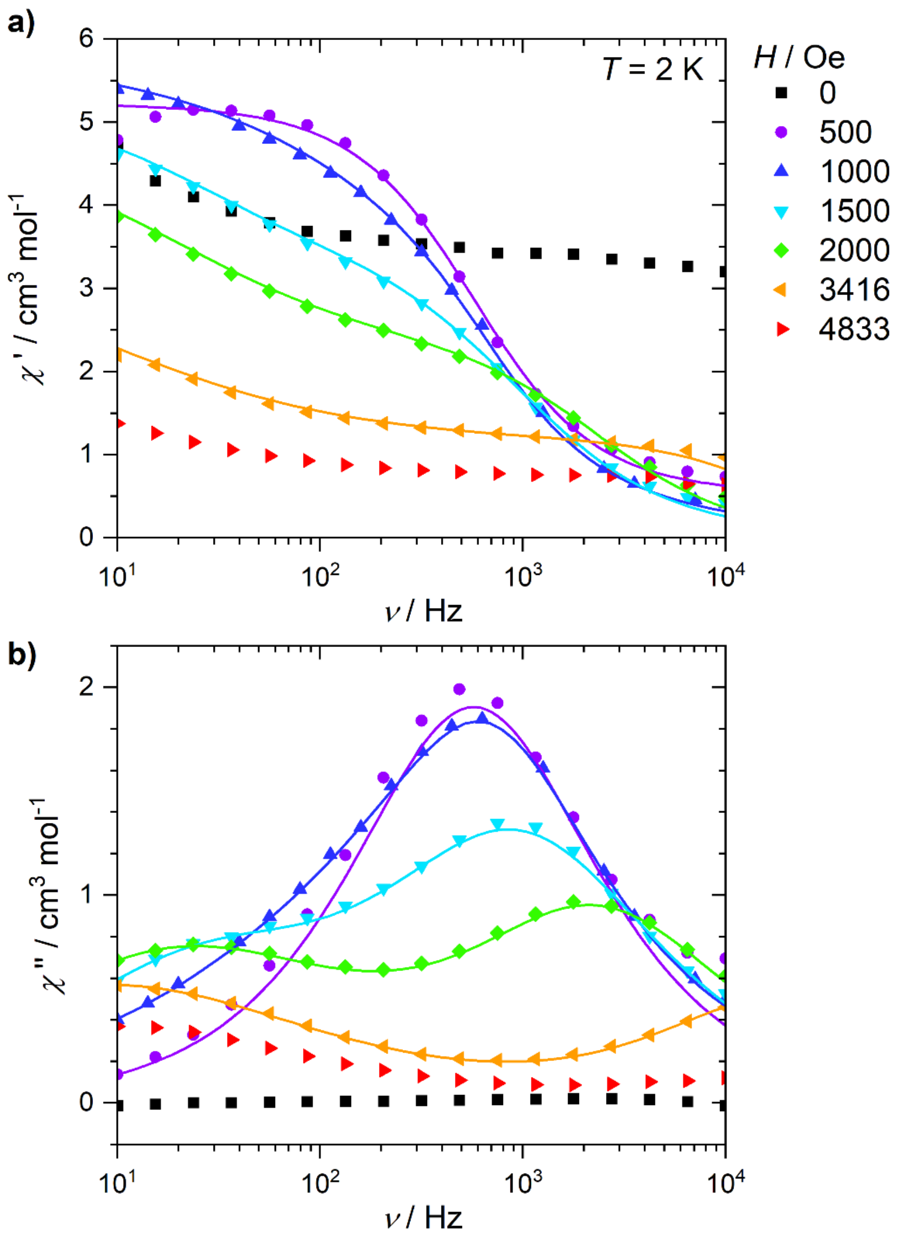

Figure S50. (a) In-phase (χ′) and **(b)** out-of-phase (χ″) ac susceptibilities for **3-Er·dcm** at 2 K and fields of 0–7666 Oe. Lines show fits to the Generalized (500 Oe) or Double Generalized Debye model.

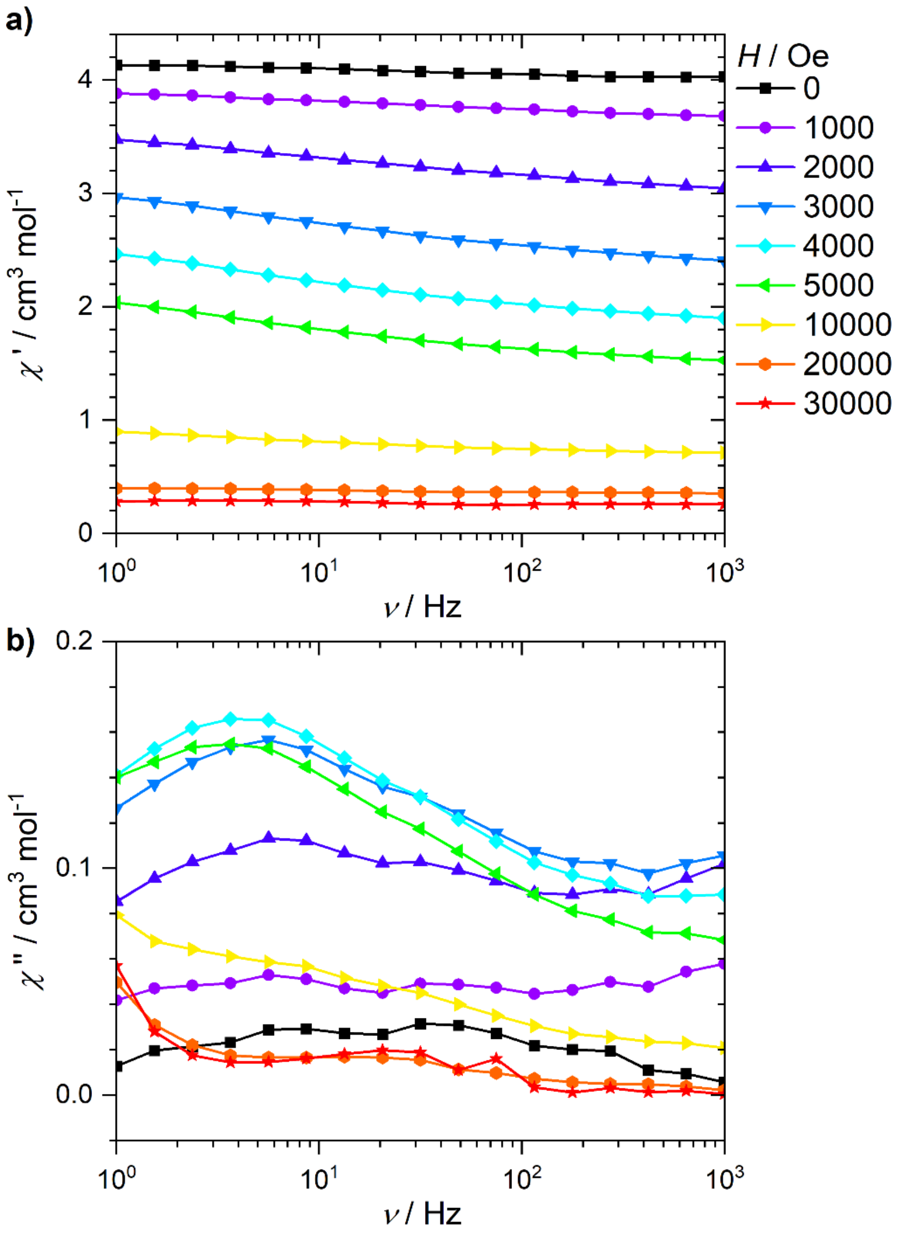

Figure S51. (a) In-phase (χ′) and **(b)** out-of-phase (χ″) ac susceptibilities for **2-Dy·tol** at 2 K and fields of 0–30000 Oe. Lines are guides for the eye.

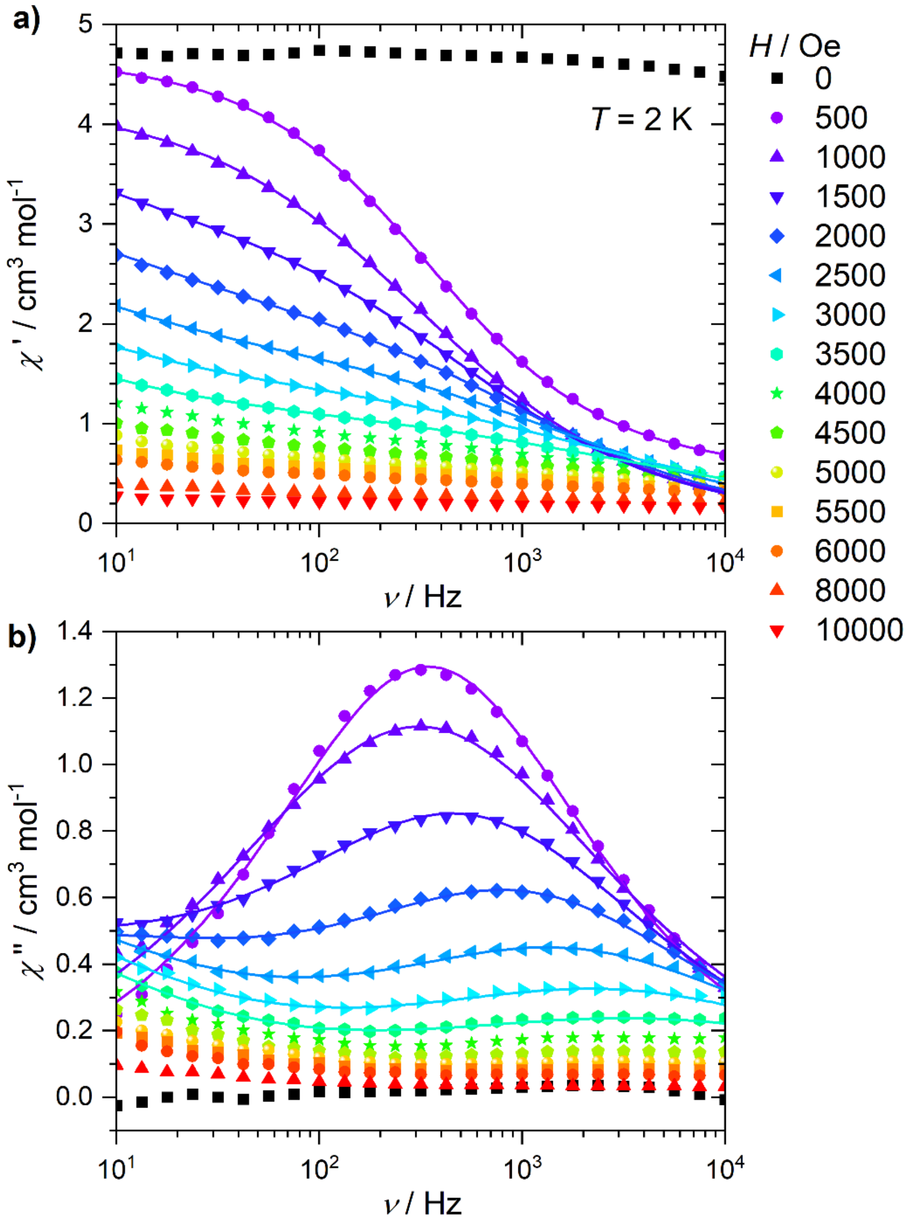

Figure S52. (a) In-phase (χ′) and **(b)** out-of-phase (χ″) ac susceptibilities for **1-Dy·0.5tol** at 2 K and fields of 0–6000 Oe. Lines show fits to the Generalized (500–1000 Oe) or Double Generalized Debye model.

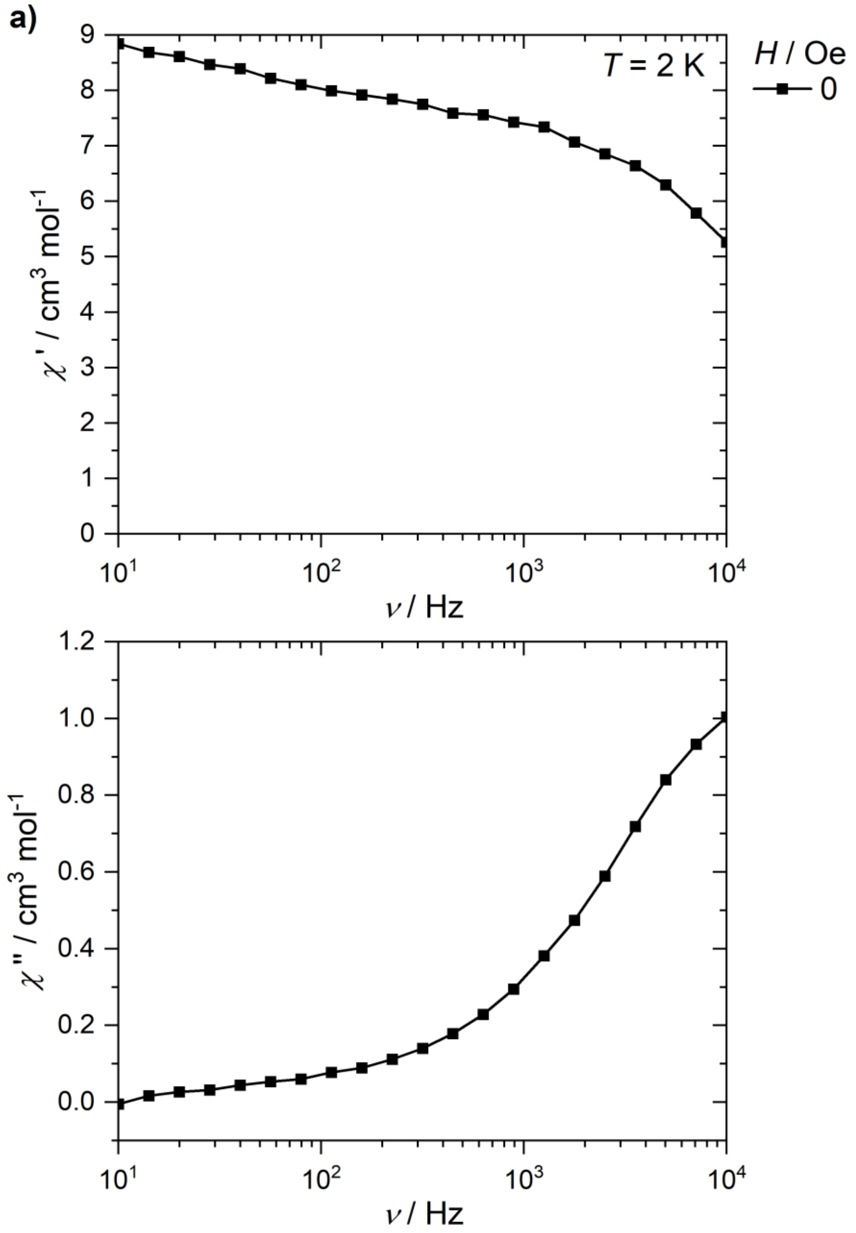

Figure S53. (a) In-phase (χ′) and **(b)** out-of-phase (χ″) ac susceptibilities for **3-Dy·2thf** at 2 K and zero field as measured on the PPMS. Lines are guides for the eye.

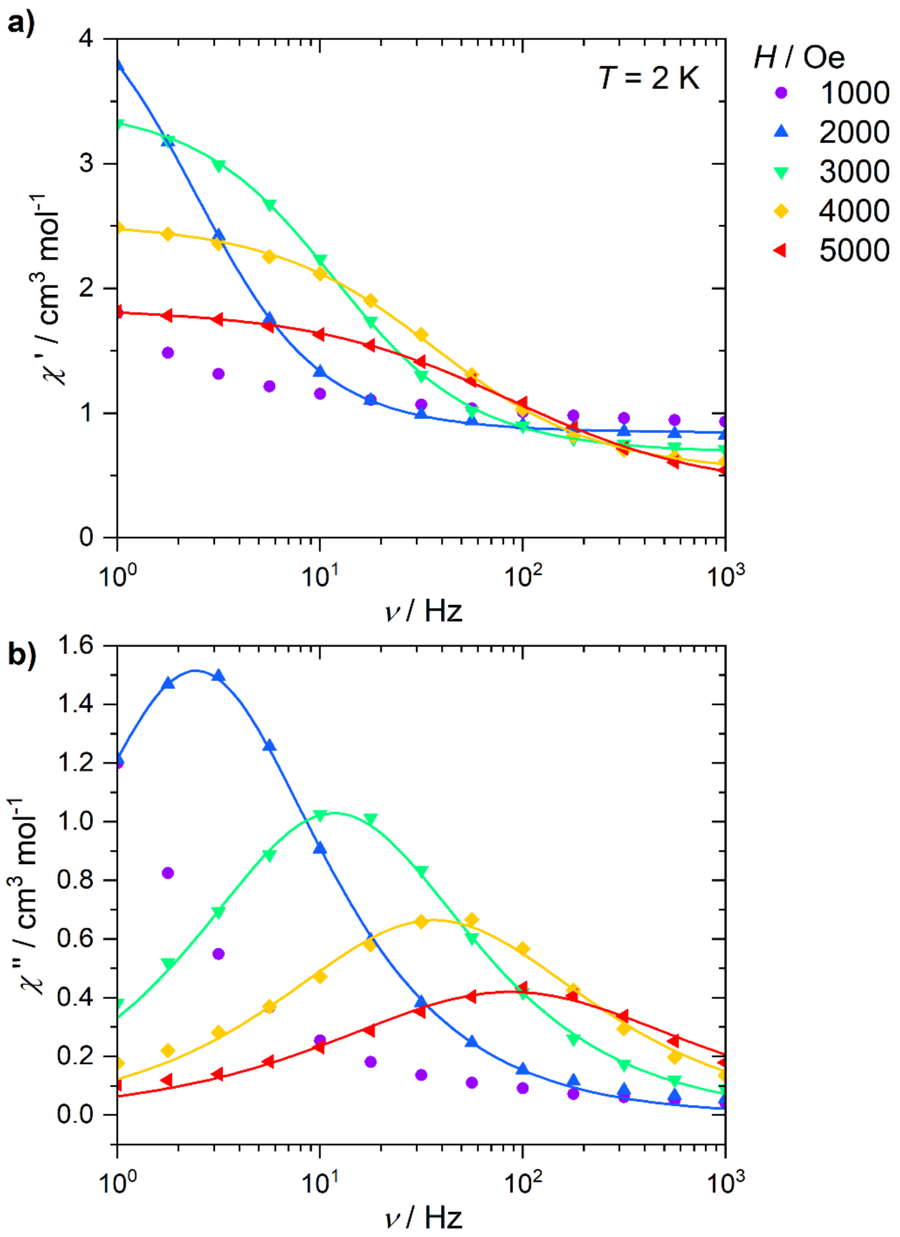

Figure S54. (a) In-phase (χ′) and **(b)** out-of-phase (χ″) ac susceptibilities for **3-Dy·2thf** at 2 K and fields of 1000–5000 Oe, measured on a SQUID magnetometer. Lines show fits to the Generalized Debye model.

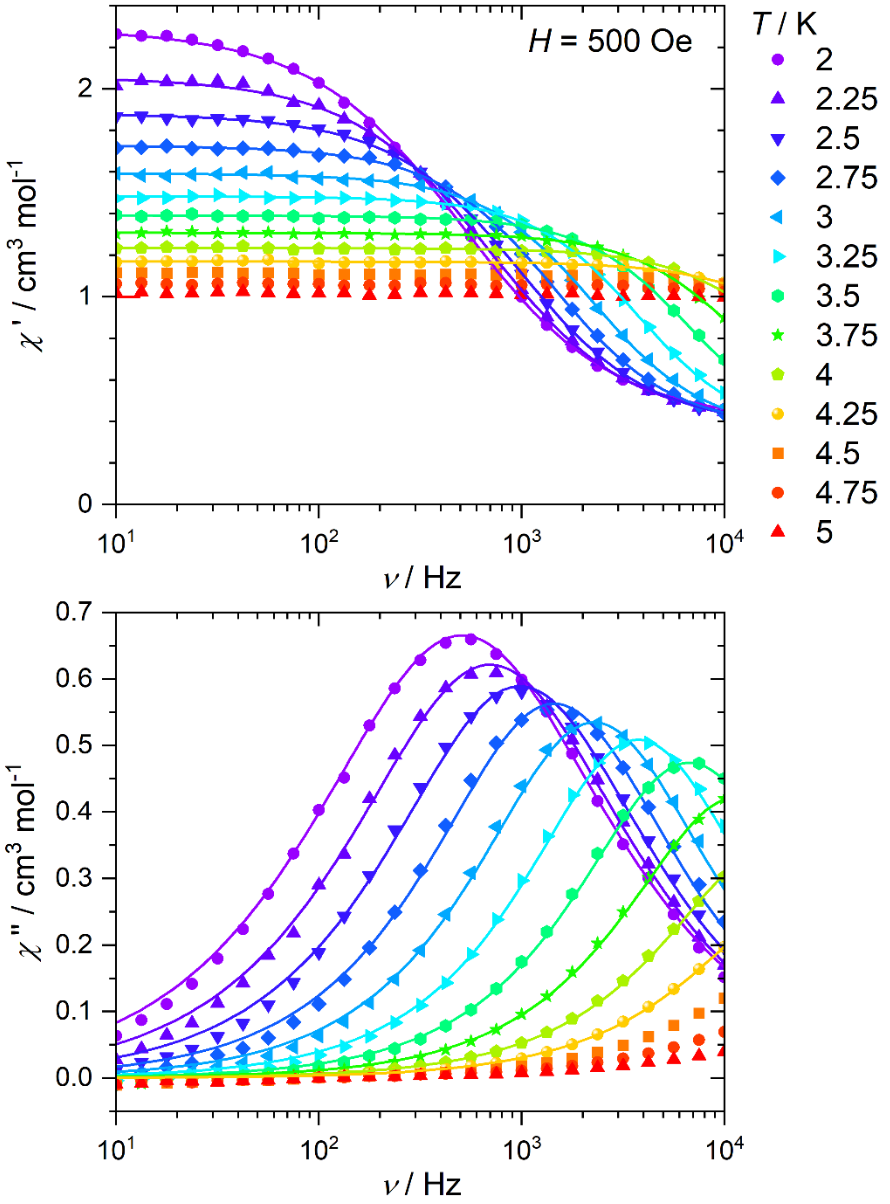

Figure S55. (a) In-phase (χ′) and **(b)** out-of-phase (χ″) ac susceptibilities for **1-Er·0.5tol** under 500 Oe and temperatures of 2–5 K. Lines show fits to the Generalized Debye model.

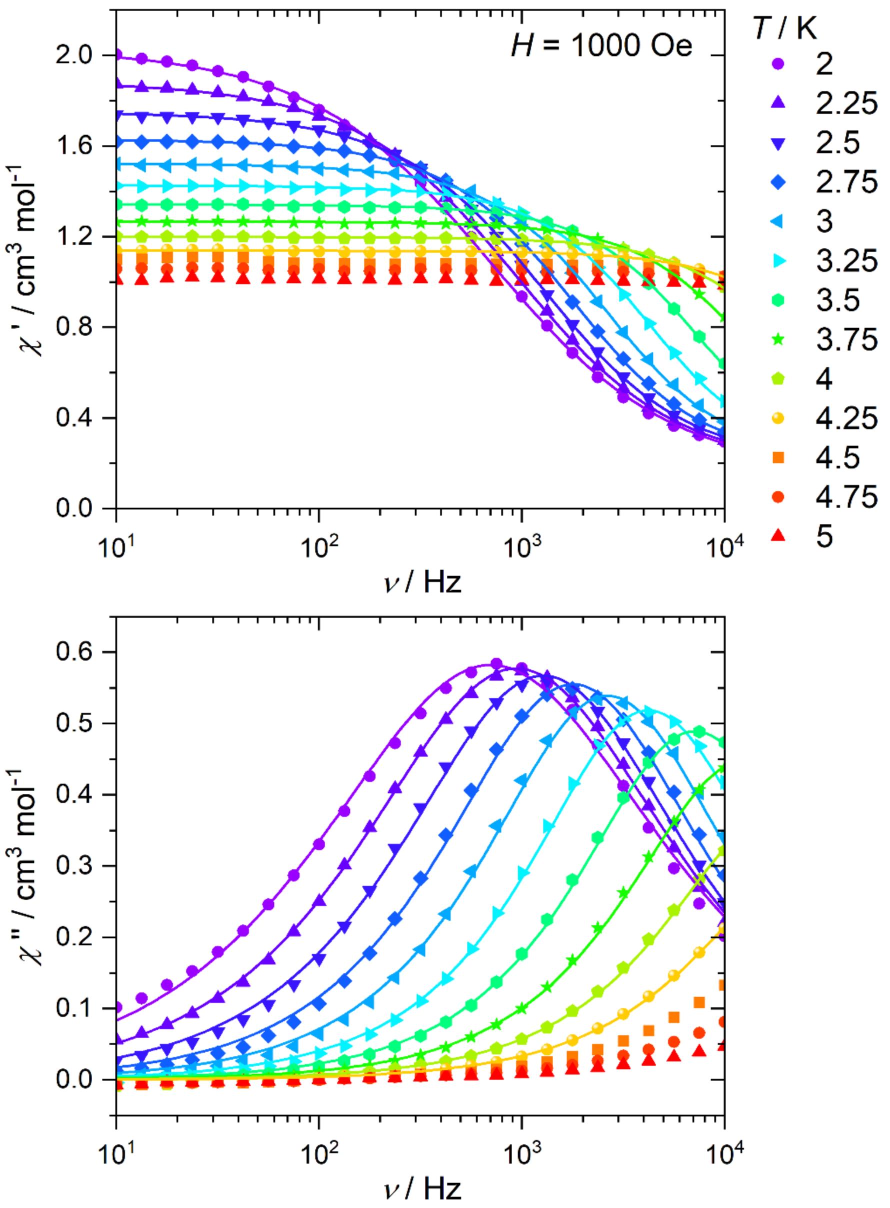

Figure S56. (a) In-phase (χ′) and **(b)** out-of-phase (χ″) ac susceptibilities for **1-Er·0.5tol** under 1000 Oe and temperatures of 2–5 K. Lines show fits to the Generalized Debye model.

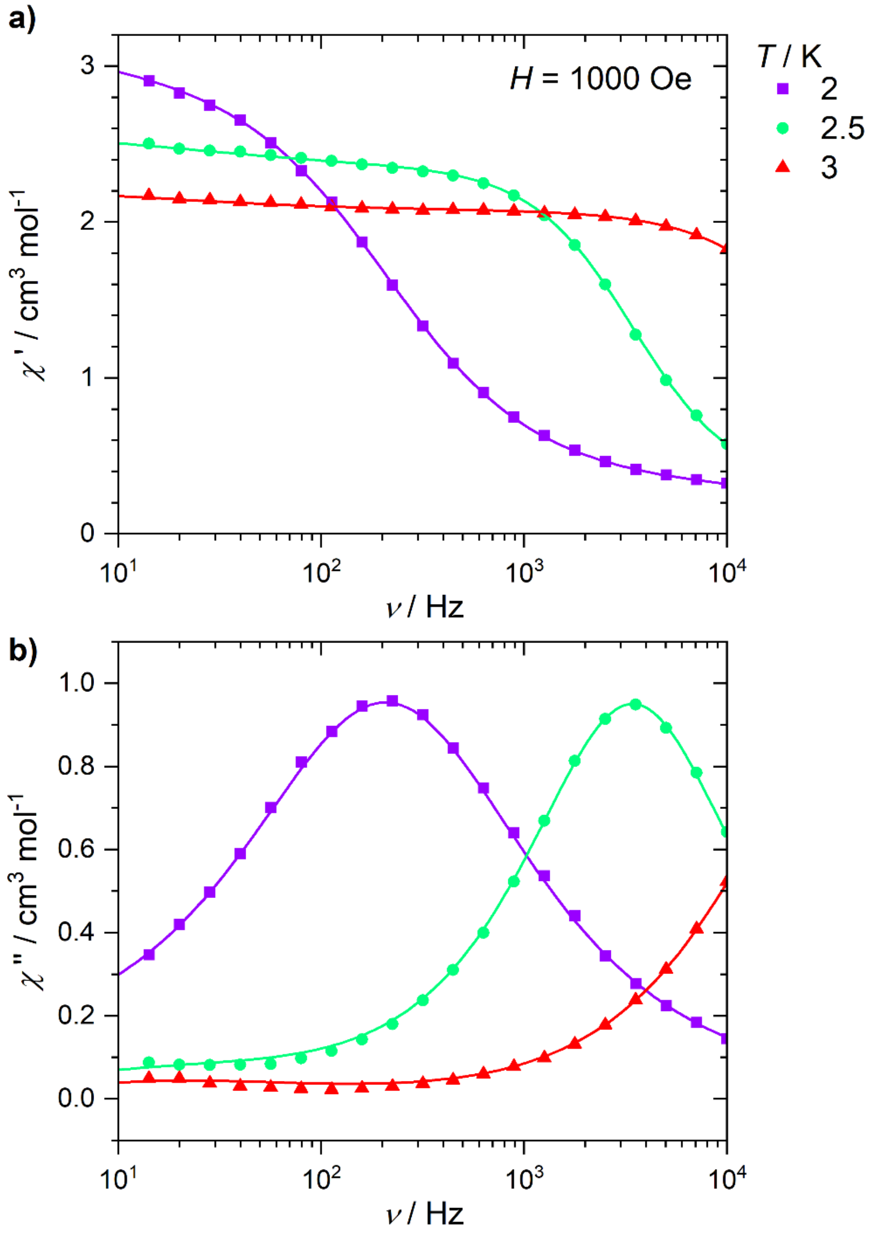

Figure S57. **(a)** In-phase (χ′) and **(b)** out-of-phase (χ″) ac susceptibilities for **2-Er·tol** under 1000 Oe and temperatures of 2–3 K. Lines show fits to the Double Generalized Debye model.

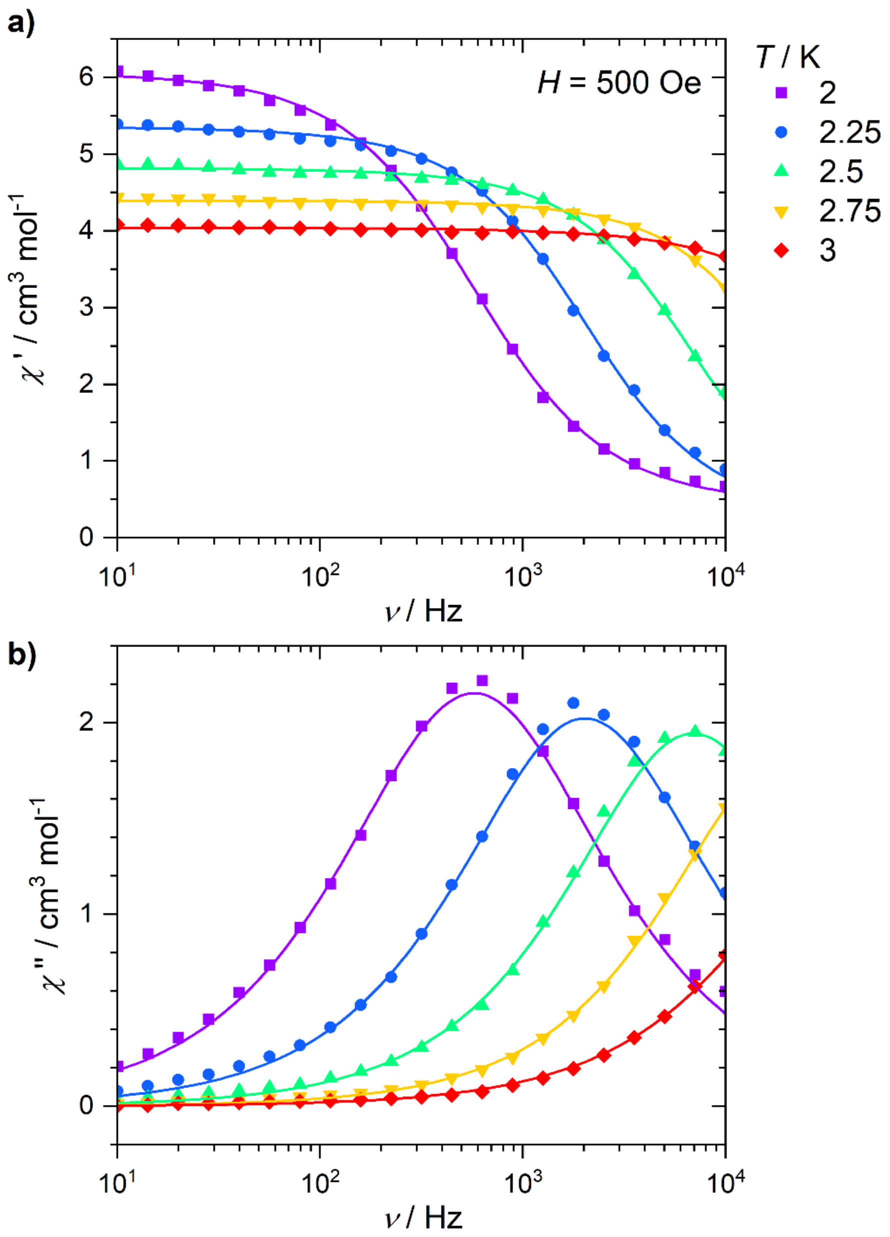

Figure S58. (a) In-phase (χ′) and **(b)** out-of-phase (χ″) ac susceptibilities for **3-Er·dcm** under 500 Oe and at temperatures of 2–3 K. Lines show fits to the Generalized Debye model.

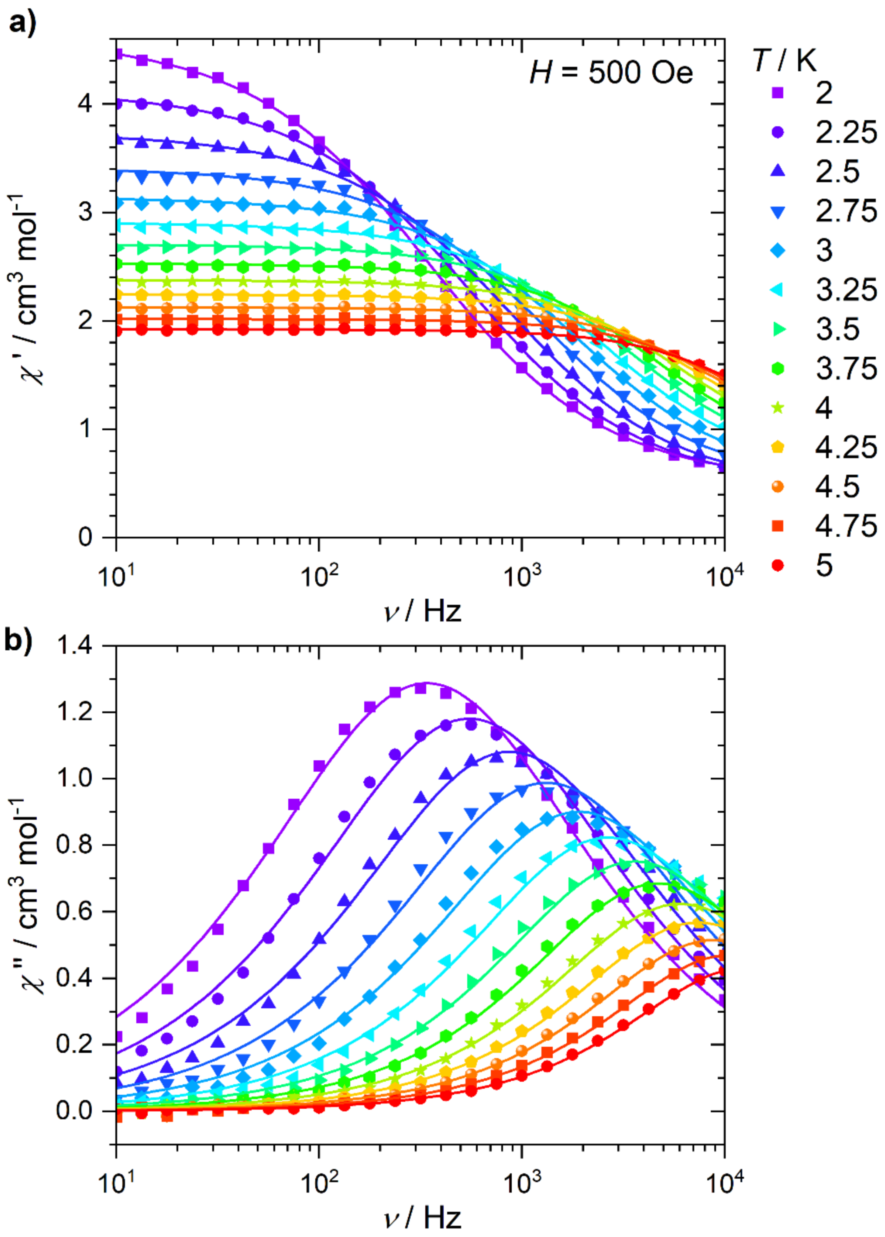

Figure S59. (a) In-phase (χ′) and **(b)** out-of-phase (χ″) ac susceptibilities for **1-Dy·0.5tol** under 500 Oe and at temperatures of 2–5 K. Lines show fits to the Generalized Debye model.

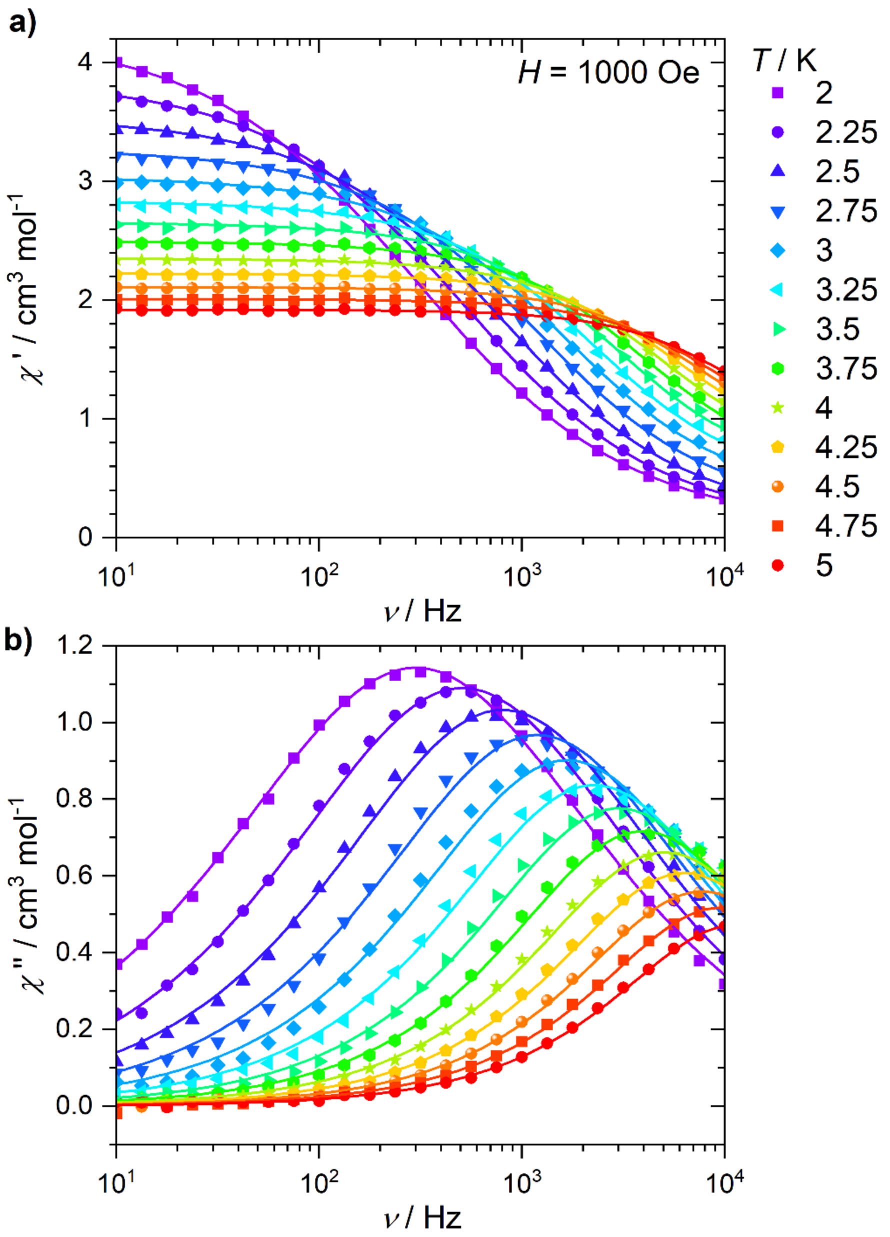

Figure S60. (a) In-phase (χ′) and **(b)** out-of-phase (χ″) ac susceptibilities for **1-Dy·0.5tol** under 1000 Oe and at temperatures of 2–5 K. Lines show fits to the Generalized Debye model.

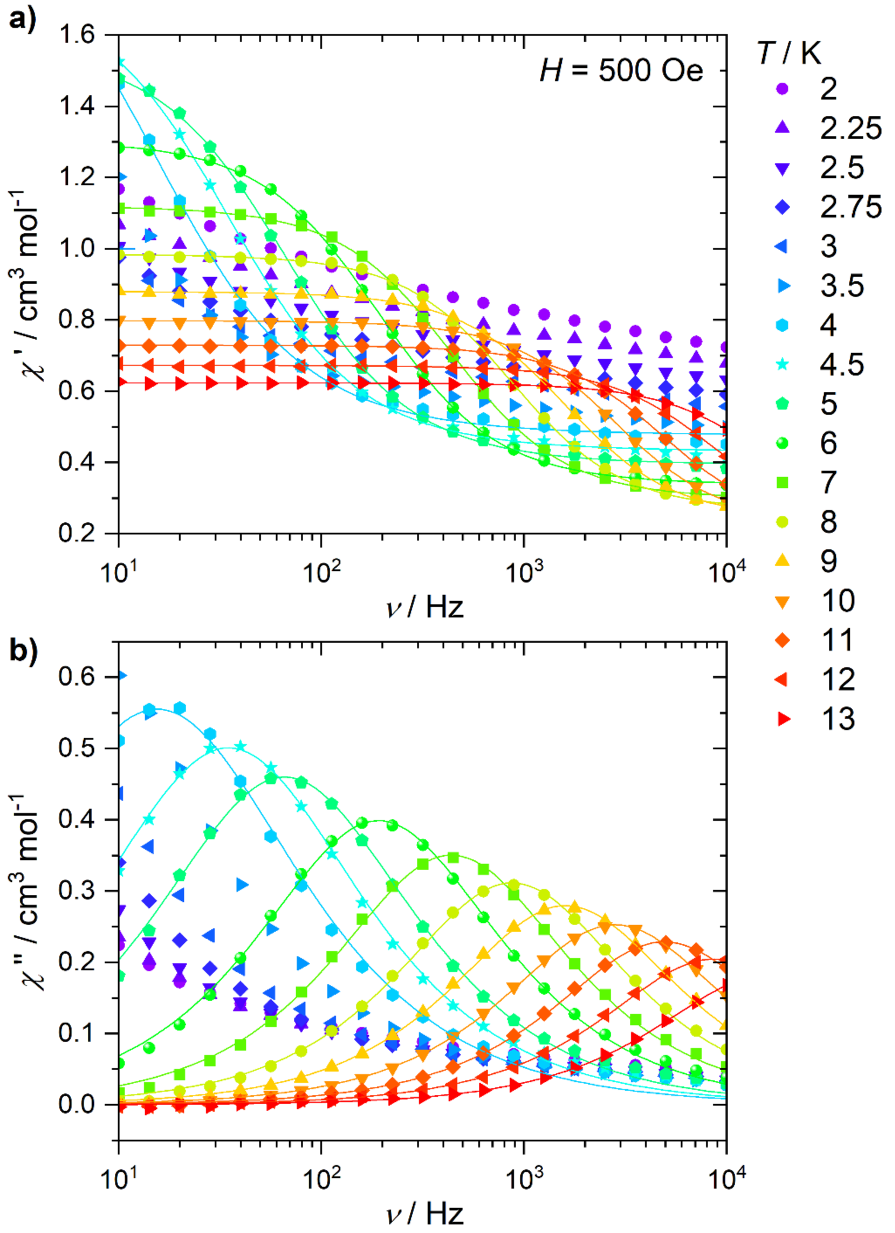

Figure S61. (a) In-phase (χ′) and **(b)** out-of-phase (χ″) ac susceptibilities for **3-Dy·2thf** under 500 Oe and at temperatures of 2–13 K. Lines indicate fits to the Generalized Debye model.

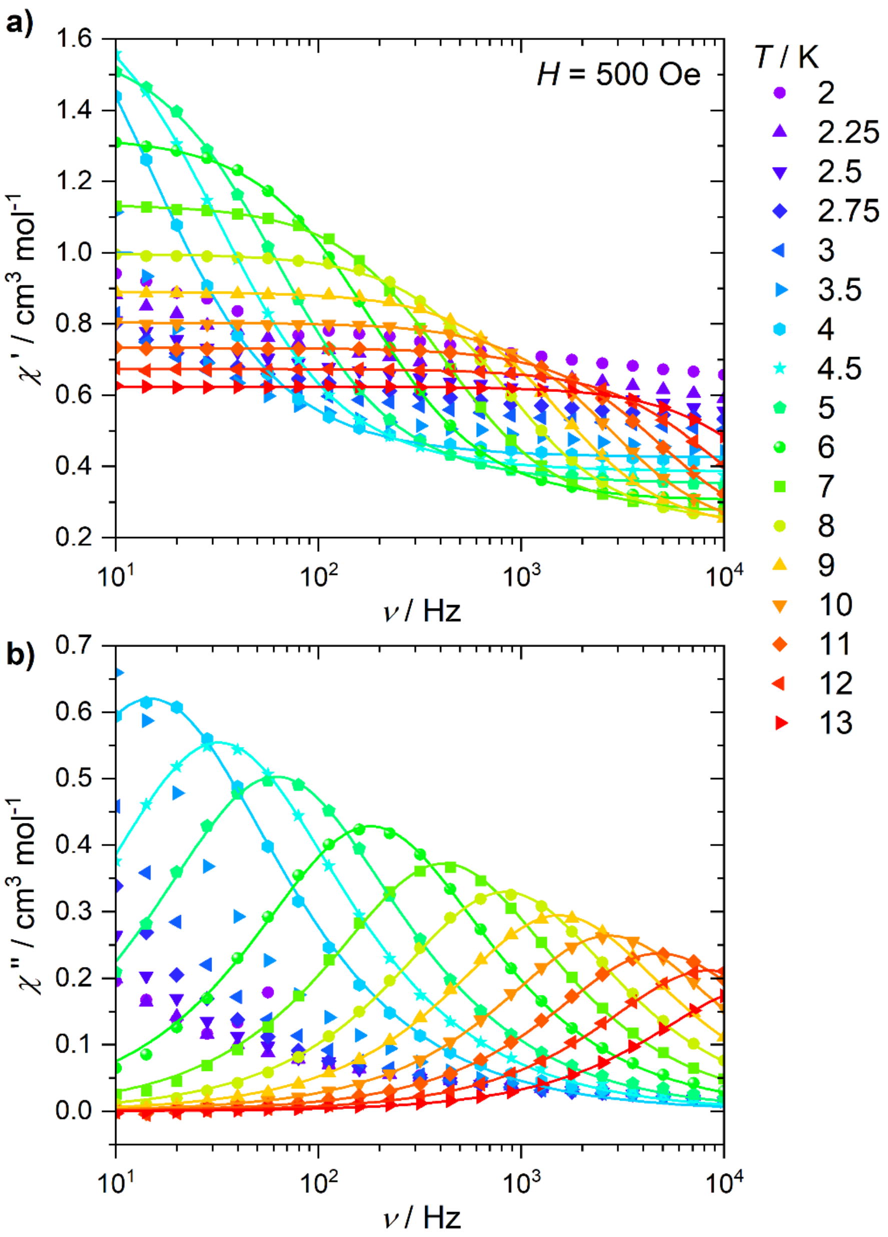

Figure S62. (a) In-phase (χ′) and **(b)** out-of-phase (χ″) ac susceptibilities for **3-Dy·2thf** under 1000 Oe and at temperatures of 2–13 K. Lines indicate fits to the Generalized Debye model.

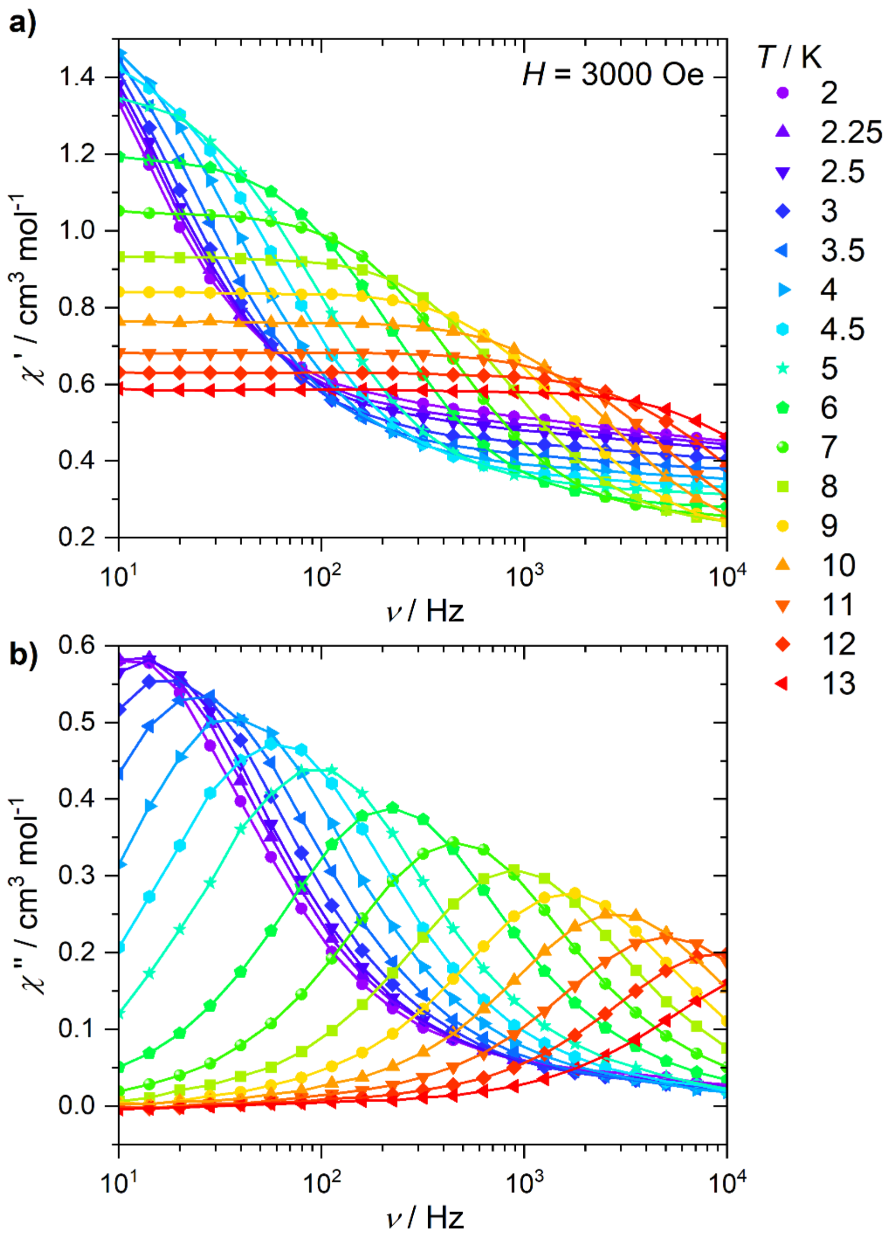

Figure S63. (a) In-phase (χ′) and **(b)** out-of-phase (χ″) ac susceptibilities for **3-Dy·2thf** under 3000 Oe and at temperatures of 2–13 K. Lines indicate fits to the Generalized Debye model.

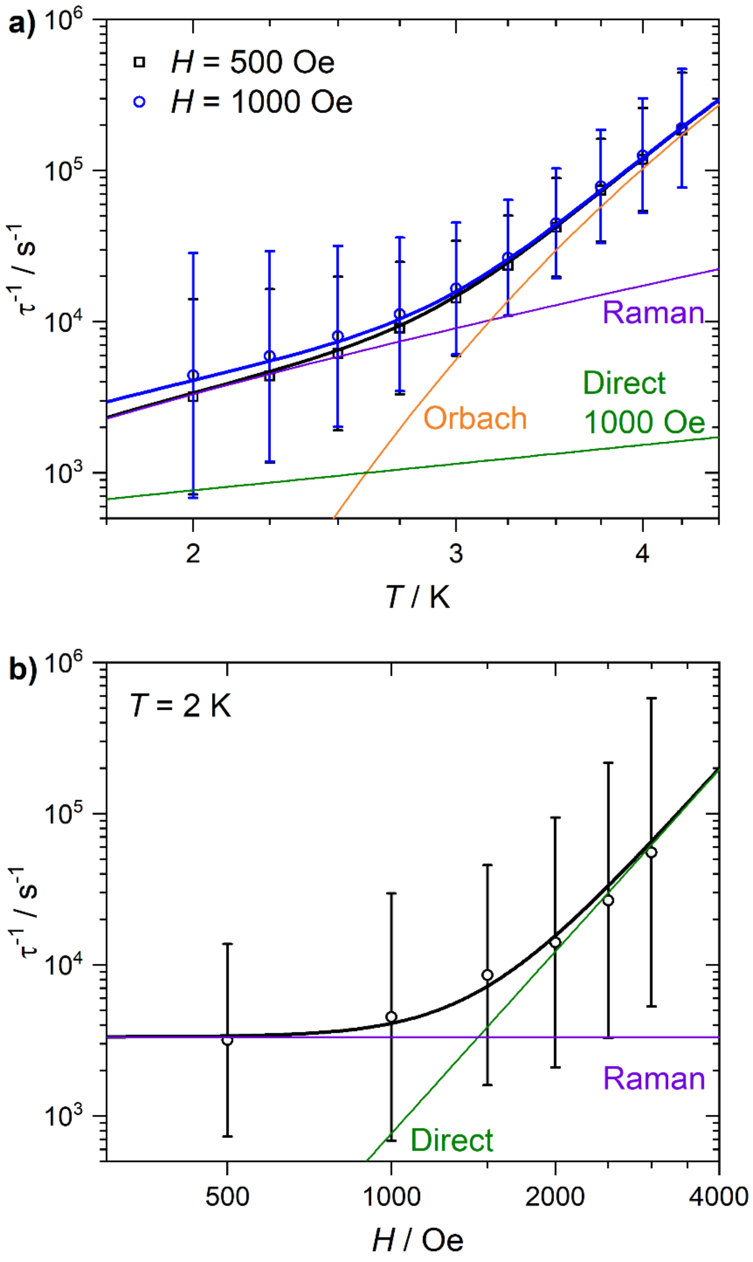

Figure S64. (a) Temperature-dependence under 500 Oe (black) or 1000 Oe (blue) dc field and **(b)** field-dependence at 2 K for the relaxation rates of **1-Er·0.5tol**. Error bars indicate 1 ESD in the distribution of rates. Thick lines indicate results obtained by simultaneously fitting all datasets to sum of Orbach (orange), phonon-pair driven Raman (purple) and Direct (green) processes: $\tau^{-1}(T,H) = \tau_0^{-1}\exp(-U_{\mathrm{eff}}/T) + R\exp(\omega/\mathrm{T})/[\exp(\omega/\mathrm{T})+1]^2 + AH^4T$ with $\tau_0 = 10^{-8.8(2)}$ s, $U_{\mathrm{eff}} = 24.2(13)$ cm^{-1}, $R = 10^{4.3(3)}$ s^{-1}, $\omega = 2.9(8)$ cm^{-1} and $A = 10^{-9.42(4)}$ s^{-1} Oe^{-4} K^{-1}. The Direct rates at 500 Oe (50–100 s^{-1}) and the Orbach rates at 2 K (17 s^{-1}) lie outside of the graph ranges.

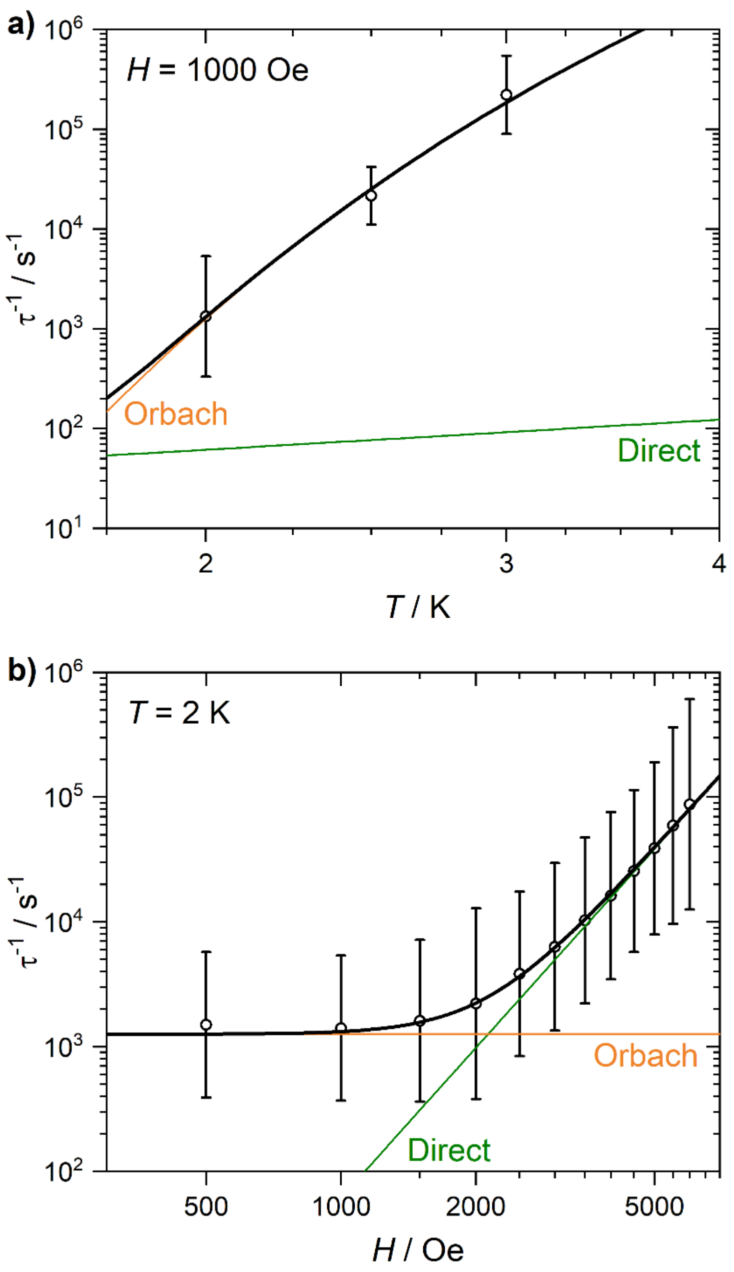

Figure S65. (a) Temperature-dependence under 1000 Oe dc field and **(b)** field-dependence at 2 K for the relaxation rates of **2-Er·tol**. Error bars indicate 1 ESD in the distribution of rates. Solid thick black line indicates fit obtained by simultaneously fitting both datasets to sum of phonon-pair driven Orbach (orange) and Direct (green) processes: $\tau^{-1}(T,H) = \tau_0^{-1}\exp(-U_{\mathrm{eff}}/T) + AH^4T$ with $\tau_0 = 10^{-9.61(12)}$ s, $U_{\mathrm{eff}} = 20.8(5)$ cm^{-1} and $A = 10^{-10.51(3)}$ s^{-1} Oe^{-4} K^{-1}.

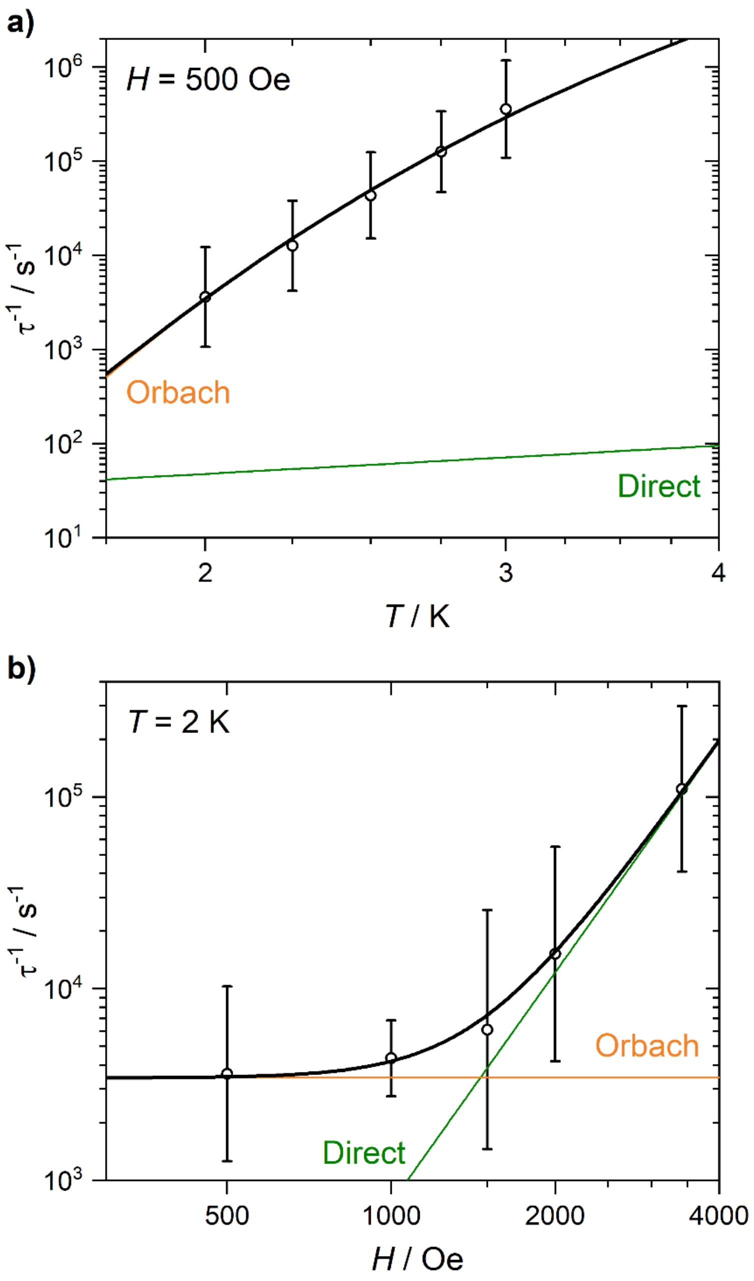

Figure S66. (a) Temperature-dependence under 500 Oe dc field and **(b)** field-dependence at 2 K for the relaxation rates of **3-Er·dcm**. Error bars indicate 1 ESD in the distribution of rates. Solid thick black line indicates fit obtained by simultaneously fitting both datasets to sum of Orbach (orange) and Direct (green) processes: $\tau^{-1}(T,H) = \tau_0^{-1}\exp(-U_{eff}/T) + AH^4T$ with $\tau_0 = 10^{-9.33(13)}$ s, $U_{eff} = 18.5(5)$ cm^{-1} and $A = 10^{-9.42(4)}$ s^{-1} Oe^{-4} K^{-1}.

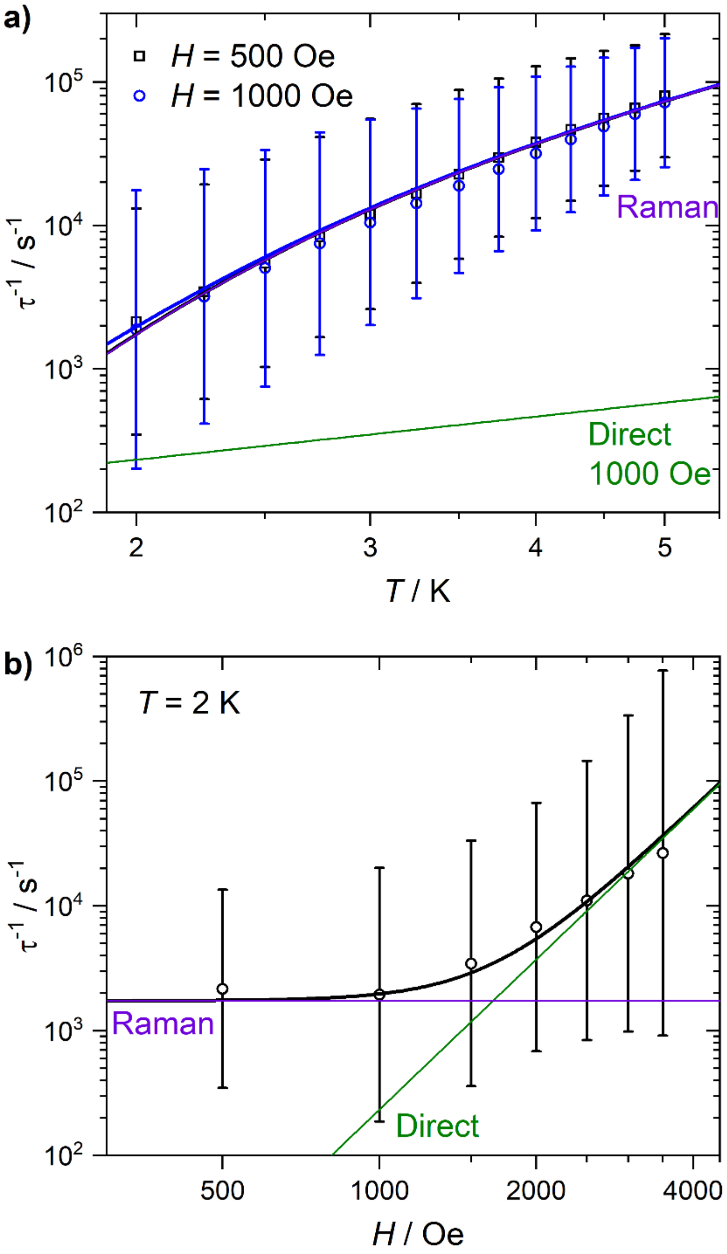

Figure S67. (a) Temperature-dependence under 500 Oe (black) or 1000 Oe (blue) dc field and **(b)** field-dependence at 2 K for the relaxation rates of **1-Dy·0.5tol**. Error bars indicate 1 ESD in the distribution of rates. Thick lines indicate result obtained by simultaneously fitting all datasets to sum of phonon-pair driven Raman (purple) and Direct (green) processes: $\tau^{-1}(T,H) = \tau_0^{-1}\exp(-U_{\mathrm{eff}}/T) + R\exp(\omega/\mathrm{T})/[\exp(\omega/\mathrm{T})+1]^2 + AH^4T$ with $\tau_0 = 10^{-8.8(2)}$ s, $U_{\mathrm{eff}} = 24.3(12)$ cm^{-1}, $R = 10^{4.4(3)}$ s^{-1}, $\omega = 3.0(8)$ cm^{-1} and $A = 10^{-9.47(3)}$ s^{-1} Oe^{-4} K^{-1}. The Direct process rates at 500 Oe are 10–40 s^{-1} and lie outside of the range shown.

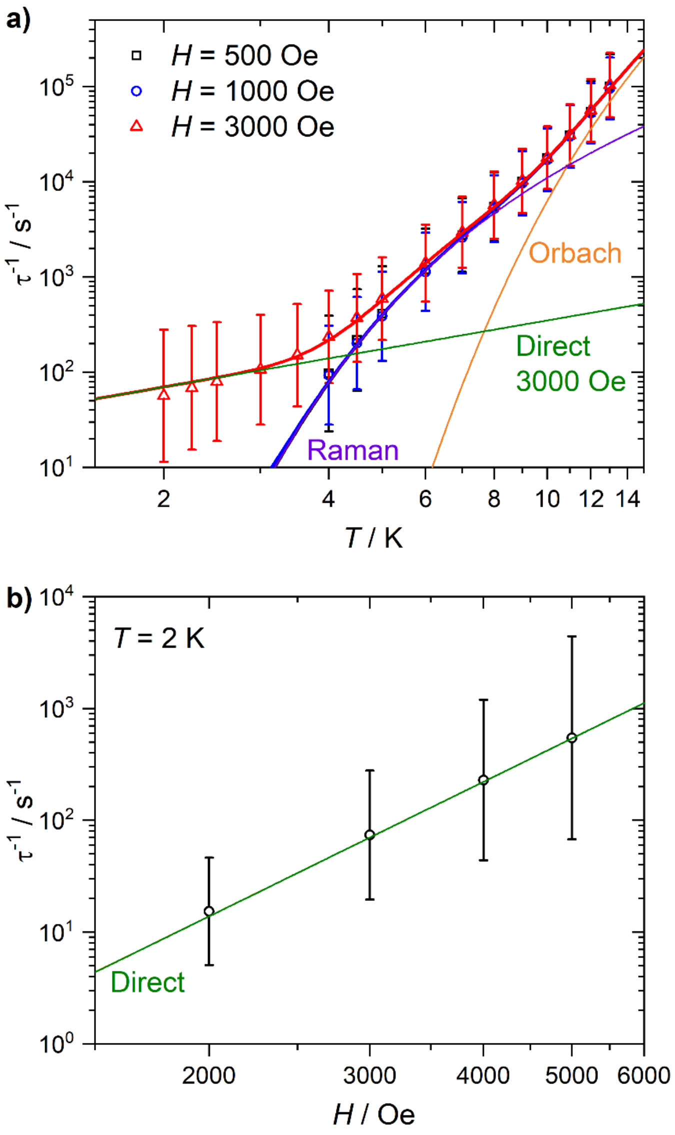

Figure S68. (a) Temperature-dependence at 500 Oe (black), 1000 Oe (blue) and 3000 Oe (red) dc field and **(b)** field-dependence at 2 K for the relaxation rates of **3-Dy·2thf**. Error bars indicate 1 ESD in the distribution of rates. The solid lines indicates result obtained by simultaneously fitting all datasets to sum of Orbach (orange), phonon-pair driven Raman (purple) and direct (green) processes: $\tau^{-1}(T,H) = \tau_0^{-1}\exp(-U_{\mathrm{eff}}/T) + R\exp(\omega/\mathrm{T})/[\exp(\omega/\mathrm{T})+1]^2 + AH^4T$ with $\tau_0 = 10^{-8.3(3)}$ s, $U_{\mathrm{eff}} = 72(6)$ cm^{-1}, $R = 10^{5.42(8)}$ s^{-1}, $\omega = 22.6(7)$ cm^{-1} and $A = 10^{-12.36(2)}$ s^{-1} Oe^{-4} K^{-1}. The direct process rates at 500 Oe (0.05–0.4 s^{-1}) and 1000 Oe (0.9–6 s^{-1}) and the 2 K Raman (0.02 s^{-1}) and Orbach (5E–15 s^{-1}) rates lie outside the range of the graph.

Table S28. Generalized Debye parameters for fitting of **1-Er·0.5tol** ac data at 2 K and variable field.

H / Oe	τ_1 / s	α_1	$\Delta\chi_1$ / cm^3 mol^{-1}	ln τ_1 / ln s	$\sigma_{<\ln \tau 1>}$ / ln s	χ_S / cm^3 mol^{-1}	τ_2 ^{a} / s	α_2 ^{a}	$\Delta\chi_2$ ^{a} / cm^3 mol^{-1}	ln τ_2 ^{a} / ln s	$\sigma_{<\ln \tau 2>}$ ^{a} / ln s
500	(3.155±0.079)E-4	0.223±0.011	1.884±0.030	–8.061	1.468	0.396±0.026	-	-	-	-	-
1000	(2.22±0.19)E-4	0.307±0.026	1.95±0.10	–8.415	1.885	0.122±0.089	-	-	-	-	-
1500	(1.17±0.17)E-4	0.27±0.16	1.28±1.26	–9.052	1.676	0.10±0.31	(5.7±25.7)E-3	0.57±1.06	0.64±2.26	–5.172	3.807
2000	(7.09±0.81)E-5	0.31±0.12	1.07±0.66	–9.554	1.903	0.069±0.154	0.079±0.929	0.64±0.89	1.0±3.9	–2.534	4.639
2500	(3.8±1.1)E-5	0.35±0.16	0.90±0.63	–10.191	2.099	0.047±0.272	0.034±0.238	0.56±0.71	0.78±2.09	–3.368	3.668
3000	(1.8±3.1)E-5	0.39±0.31	0.77±1.30	–10.923	2.349	0.00±0.97	0.24±3.07	0.61±0.60	1.3±4.5	–1.436	4.334

^{a} The second relaxation process is required to fit the data but relaxation parameters are unreliable as the full process is not observed.

Table S29. Generalized Debye parameters for fitting of **1-Er·0.5tol** ac data at 500 Oe and variable temperature.

T / K	τ / s	α	Δχ / cm^3 mol^{-1}	ln τ / ln s	$\sigma_{<\ln \tau>}$ / ln s	χ_S / cm^3 mol^{-1}
2	(3.129±0.087)E-4	0.227±0.012	1.917±0.034	–8.070	1.488	0.382±0.029
2.25	(2.28±0.11)E-4	0.192±0.020	1.689±0.055	–8.387	1.322	0.373±0.050
2.5	(1.623±0.079)E-4	0.160±0.021	1.520±0.056	–8.726	1.172	0.363±0.052
2.75	(1.100±0.060)E-4	0.126±0.023	1.374±0.066	–9.115	1.007	0.355±0.063
3	(6.98±0.45)E-5	0.100±0.027	1.252±0.085	–9.570	0.876	0.341±0.084
3.25	(4.23±0.42)E-5	0.078±0.038	1.15±0.15	–10.070	0.760	0.33±0.15
3.5	(2.37±0.46)E-5	0.076±0.072	1.07±0.35	–10.650	0.753	0.32±0.35
3.75	(1.35±0.26)E-5	0.081±0.069	0.96±0.30	–11.216	0.779	0.34±0.30
4	(8.4±2.9)E-6	0.082±0.095	0.81±0.33	–11.684	0.786	0.43±0.33
4.25	(5.4±4.7)E-6	0.099±0.174	0.67±0.56	–12.130	0.875	0.50±0.56

Table S30. Generalized Debye parameters for fitting of **1-Er·0.5tol** ac data at 1000 Oe and variable temperature.

T / K	τ / s	α	Δχ / cm^3 mol^{-1}	ln τ / ln s	$\sigma_{<\ln \tau>}$ / ln s	χ_S / cm^3 mol^{-1}
2	(2.26±0.16)E-4	0.303±0.021	1.911±0.079	–8.394	1.865	0.129±0.070
2.25	(1.687±0.045)E-4	0.2506±0.0087	1.730±0.030	–8.688	1.603	0.159±0.028
2.5	(1.248±0.041)E-4	0.204±0.011	1.571±0.038	–8.988	1.377	0.183±0.036
2.75	(8.912±0.49)E-5	0.159±0.020	1.430±0.068	–9.326	1.168	0.201±0.066
3	(6.02±0.44)E-5	0.125±0.026	1.31±0.10	–9.719	1.005	0.21±0.10
3.25	(3.78±0.43)E-5	0.101±0.039	1.22±0.18	–10.184	0.883	0.21±0.18
3.5	(2.23±0.39)E-5	0.092±0.060	1.13±0.32	–10.711	0.837	0.21±0.32
3.75	(1.27±0.23)E-5	0.097±0.059	1.04±0.29	–11.273	0.862	0.22±0.29
4	(7.9±2.1)E-6	0.098±0.070	0.89±0.28	–11.744	0.871	0.30±0.28
4.25	(5.2±2.6)E-6	0.105±0.095	0.74±0.34	–12.160	0.902	0.40±0.35

Table S31. Generalized Debye parameters for fitting of **2-Er·tol** ac data at 2 K and variable field.

H / Oe	τ_1 / s	α_1	$\Delta\chi_1$ / cm^3 mol^{-1}	ln τ_1 / ln s	$\sigma_{<\ln \tau 1>}$ / ln s	χ_S / cm^3 mol^{-1}	τ_2 ^{a} / s	α_2 ^{a}	$\Delta\chi_2$ ^{a} / cm^3 mol^{-1}	ln τ_2 ^{a} / ln s	$\sigma_{<\ln \tau 2>}$ ^{a} / ln s
500	(6.68±0.24)E–4	0.196±0.019	2.756±0.065	–7.311	1.342	0.372±0.046	-	-	-	-	-
1000	(7.1±1.2)E–4	0.20±0.17	2.3±2.2	–7.249	1.339	0.080±8.200	0.16±70.18	0.81±6.82	1.4±71.6	–1.813	9.540
1500	(6.20±0.94)E–4	0.23±0.11	1.9±1.1	–7.387	1.491	0.15±0.52	0.49±33.91	0.74±0.90	2.2±21.7	–0.709	6.611
2000	(4.5±1.8)E–4	0.28±0.15	1.8±2.0	–7.704	1.762	0.23±0.13	0.064±2.15	0.44±3.71	1.1±17.2	–2.742	2.686
2500	(2.61±0.30)E–4	0.23±0.23	1.1±1.5	–8.251	1.518	0.19±0.82	0.13±2.68	0.63±1.38	2.2±13.4	–2.007	4.600
3000	(1.58±0.23)E–4	0.24±0.10	0.97±0.49	–8.752	1.547	0.222±0.069	0.066±0.610	0.50±0.77	1.6±5.5	–2.723	3.177
3500	(9.7±1.2)E–5	0.235±0.097	0.81±0.28	–9.240	1.529	0.220±0.082	0.039±0.162	0.44±0.44	1.2±2.2	–3.251	2.661
4000	(6.2±1.1)E–5	0.24±0.13	0.68±0.27	–9.695	1.540	0.22±0.15	0.037±0.133	0.41±0.37	1.0±1.7	–3.297	2.462
4500	(3.9±1.5)E–5	0.23±0.17	0.54±0.33	–10.148	1.495	0.21±0.24	0.073±0.413	0.45±0.36	1.3±2.8	–2.617	2.777
5000	(2.6±2.8)E–5	0.25±0.29	0.47±0.62	–10.570	1.592	0.20±0.55	0.058±0.305	0.43±0.38	1.0±2.2	–2.848	2.598
5500	(1.7±3.5)E–5	0.29±0.34	0.43±0.92	–10.991	1.815	0.18±0.88	0.040±0.114	0.37±0.26	0.76±1.01	–3.223	2.234
6000	(1.1±4.9)E–5	0.32±0.51	0.38±1.58	–11.382	1.941	0.17±1.56	0.043±0.120	0.37±0.24	0.70±0.90	–3.143	2.245

[a] The second relaxation process is required to fit the data but relaxation parameters are unreliable as the full process is not observed.

Table S32. Generalized Debye parameters for fitting of **2-Er·tol** ac data at 1000 Oe and variable temperature.

T / K	τ_1 / s	α_1	$\Delta\chi_1$ / cm^3 mol^{-1}	ln τ_1 / ln s	$\sigma_{<\ln \tau 1>}$ / ln s	χ_S / cm^3 mol^{-1}	τ_2 ^{a} / s	α_2 ^{a}	$\Delta\chi_2$ ^{a} / cm^3 mol^{-1}	ln τ_2 ^{a} / ln s	$\sigma_{<\ln \tau 2>}$ ^{a} / ln s
2	(7.5±1.0)E-4	0.21±0.12	2.4±1.6	–7.197	1.389	0.16±1.86	0.11±18.1	0.76±3.29	1.2±25.9	–2.210	7.385
2.5	(4.62±0.32)E-5	0.061±0.051	2.08±0.30	–9.982	0.665	0.28±0.23	(8.1±24.1)E-3	0.37±0.81	0.24±0.44	–4.811	2.214
3	(4.5±12.1)E-6	0.10±0.59	2.0±6.6	–12.306	0.902	0.11±6.77	(8.23±24.6)E-3	0.29±1.28	0.13±0.41	–4.800	1.795

^{a} The second relaxation process is required to fit the data but relaxation parameters are unreliable as the full process is not observed.

Table S33. Generalized Debye parameters for fitting of **3-Er·dcm** ac data at 2 K and variable field.

H / Oe	τ_1 / s	α_1	$\Delta\chi_1$ / cm^3 mol^{-1}	ln τ_1 / ln s	$\sigma_{<\ln \tau1>}$ / ln s	χ_S / cm^3 mol^{-1}					
500	(2.78±0.56)E-4	0.13±0.10	4.72±0.66	–8.187	1.051	0.52±0.58	-	-	-	-	-
H / Oe	τ_1 / s	α_1	χ_T / cm^3 mol^{-1}	ln τ_1 / ln s	$\sigma_{<\ln \tau1>}$ / ln s	χ_S / cm^3 mol^{-1}	τ_2 [a] / s	α_2 [a]	χ_{mid} / cm^3 mol^{-1}	ln τ_2 [a] / ln s	$\sigma_{<\ln \tau2>}$ [a] / ln s
1000	(2.3±5.4)E-4	0.030±2.981	5.8±2.6	–8.382	0.440	0.00±0.14	(6.2±64.4)E-4	0.44±1.52	2.2±27.8	–7.393	2.639
1500	(1.6±4.9)E-4	0.22±0.99	5.2±5.7	–8.720	1.436	0.01±1.80	(6.6±63.4)E-3	0.26±3.94	3.4±9.5	–5.025	1.655
2000	(6.6±13.6)E-5	0.18±0.85	4.8±6.1	–9.627	1.298	0.000±0.001	(8.0±32.0)E-3	0.34±2.19	2.3±4.6	–4.814	2.089
3416	(9.1±32.0)E-6	0.12±2.56	3.5±14.2	–11.513	0.912	0.00±0.92	(1.7±30.3)E-2	0.42±2.77	1.2±2.3	–4.088	2.543

[a] To get reliable fits, a different MATLAB program was used fitting to $\boldsymbol{\tau^{-1}} = \boldsymbol{\chi_s} + \frac{\chi_T - \chi_{mid}}{1+(i\omega\tau_2)^{1-\alpha_2}} + \frac{\chi_{mid} - \chi_S}{1+(i\omega\tau_1)^{1-\alpha_1}}$. [b] The second relaxation process is required to fit the data but relaxation parameters are unreliable as the full process is not observed.

Table S34. Generalized Debye parameters for fitting of **3-Er·dcm** ac data at 500 Oe and variable temperature.

T / K	τ / s	α	Δχ / cm^3 mol^{-1}	ln τ / ln s	$\sigma_{<\ln \tau>}$ / ln s	χ_S / cm^3 mol^{-1}
2	(2.77±0.18)E-4	0.171±0.030	5.65±0.25	-8.191	1.223	0.42±0.22
2.25	(7.88±0.84)E-5	0.145±0.039	5.09±0.49	-9.449	1.102	0.26±0.48
2.5	(2.3±1.2)E-5	0.14±0.14	4.8±3.5	-10.679	1.054	0.0±3.5
2.75	(7.9±4.2)E-6	0.12±0.13	4.4±2.8	-11.751	0.984	0.0±2.8
3	(2.8±8.5)E-6	0.16±0.39	4.0±10.1	-12.793	1.191	0.0±10.1

Table S35. Generalized Debye parameters for fitting of **1-Dy·0.5tol** ac data at 2 K and variable field.

H / Oe	τ_1 / s	α_1	$\Delta\chi_1$ / cm^3 mol^{-1}	ln τ_1 / ln s	$\sigma_{<\ln \tau1>}$ / ln s	χ_S / cm^3 mol^{-1}	τ_2 ^{a} / s	α_2 ^{a}	$\Delta\chi_2$ ^{a} / cm^3 mol^{-1}	ln τ_2 ^{a} / ln s	$\sigma_{<\ln \tau2>}$ ^{a} / ln s
500	4.61±0.17E-4	0.296±0.015	4.20±0.10	–7.681	1.831	0.499±0.076	-	-	-	-	-
1000	5.14±0.23E-4	0.387±0.015	4.27±0.11	–7.573	2.341	0.010±0.085	-	-	-	-	-
1500	2.9±1.4E-4	0.37±0.14	2.9±3.0	–8.149	2.265	0.00±0.042	0.089±1.921	0.52±2.27	2.1±18.5	–2.417	3.271
2000	1.47±0.63E-4	0.38±0.13	2.0±1.7	–8.823	2.294	0.00±0.038	0.045±0.391	0.50±1.11	2.1±8.4	–3.097	3.162
2500	9.0±2.9E-5	0.42±0.18	1.7±1.3	–9.313	2.577	0.00±0.11	0.17±2.23	0.48±0.84	2.9±14.5	–1.784	2.994
3000	5.5±4.7E-5	0.47±0.35	1.4±1.9	–9.810	2.916	0.00±0.26	0.20±3.61	0.45±1.01	2.8±20.1	–1.596	2.788
3500	3.8±9.1E-5	0.53±0.48	1.2±2.3	–10.184	3.369	0.00±0.40	0.11±1.20	0.38±0.88	1.9±10.1	–2.211	2.290

^{a} The second relaxation process is required to fit the data but relaxation parameters are unreliable as the full process is not observed.

Table S36. Generalized Debye parameters for fitting of **1-Dy·0.5tol** ac data at 500 Oe and variable temperature.

T / K	τ / s	α	Δχ / cm^3 mol^{-1}	ln τ / ln s	$\sigma_{<\ln \tau>}$ / ln s	χ_S / cm^3 mol^{-1}
2	(4.68±0.22)E-4	0.293±0.019	4.15±0.12	–7.667	1.815	0.478±0.093
2.25	(2.90±0.20)E-4	0.274±0.025	3.69±0.16	–8.146	1.720	0.45±0.13
2.5	(1.83±0.19)E-4	0.263±0.034	3.31±0.22	–8.604	1.666	0.43±0.20
2.75	(1.21±0.19)E-4	0.251±0.047	2.96±0.33	–9.022	1.606	0.45±0.31
3	(8.3±1.7)E-5	0.235±0.056	2.63±0.42	–9.394	1.525	0.51±0.40
3.25	(6.0±1.5)E-5	0.216±0.062	2.33±0.48	–9.725	1.434	0.58±0.48
3.5	(4.4±1.3)E-5	0.199±0.070	2.06±0.59	–10.031	1.353	0.64±0.58
3.75	(3.4±1.3)E-5	0.181±0.089	1.83±0.76	–10.298	1.269	0.70±0.76
4	(2.6±1.3)E-5	0.17±0.12	1.63±0.99	–10.546	1.217	0.74±1.00
4.25	(2.1±1.2)E-5	0.15±0.14	1.5±1.1	–10.749	1.144	0.79±1.09
4.5	(1.79±0.83)E-5	0.14±0.13	1.29±0.88	–10.929	1.080	0.84±0.89
4.75	(1.52±0.57)E-5	0.13±0.11	1.14±0.66	–11.095	1.007	0.87±0.67
5	(1.25±0.35)E-5	0.122±0.084	1.05±0.43	–11.293	0.991	0.87±0.44

Table S37. Generalized Debye parameters for fitting of **1-Dy·0.5tol** ac data at 1000 Oe and variable temperature.

T / K	τ / s	α	Δχ / cm^3 mol^{-1}	ln τ / ln s	$\sigma_{<\ln \tau>}$ / ln s	χ_S / cm^3 mol^{-1}
2	(1.39±0.41)E-5	0.132±0.086	1.16±0.51	–11.181	1.036	0.76±0.52
2.25	(1.67±0.64)E-5	0.14±0.11	1.29±0.74	–10.999	1.062	0.73±0.75
2.5	(2.05±0.90)E-5	0.15±0.12	1.41±0.88	–10.797	1.105	0.70±0.89
2.75	(2.5±1.0)E-5	0.16±0.10	1.56±0.85	–10.589	1.167	0.66±0.85
3	(3.15±0.99)E-5	0.173±0.076	1.74±0.63	–10.365	1.235	0.61±0.64
3.25	(4.0±1.10E-5	0.190±0.065	1.94±0.53	–10.115	1.315	0.55±0.53
3.5	(5.3±1.3)E-5	0.207±0.060	2.16±0.47	–9.845	1.396	0.49±0.46
3.75	(7.0±1.5)E-5	0.234±0.053	2.44±0.40	–9.564	1.523	0.40±0.39
4	(9.5±1.8)E-5	0.260±0.049	2.75±0.37	–9.258	1.649	0.30±0.35
4.25	(1.34±0.18)E-4	0.287±0.036	3.09±0.26	–8.921	1.785	0.19±0.25
4.5	(1.98±0.16)E-4	0.309±0.023	3.43±0.17	–8.526	1.899	0.12±0.15
4.75	(3.13±0.13)E-4	0.336±0.013	3.794±0.088	–8.070	2.043	0.078±0.075
5	(5.31±0.13)E-4	0.370±0.009	4.231±0.063	–7.541	2.233	0.051±0.048

Table S38. Generalized Debye parameters for fitting of **3-Dy·2thf** ac data at 2 K and variable field.

H / Oe	τ / s	α	Δχ / cm^3 mol^{-1}	χ_S / cm^3 mol^{-1}	ln τ / ln s	$\sigma_{<\ln \tau>}$ / ln s
2000	(6.51 ± 0.28)E–2	0.146 ± 0.018	3.821 ± 0.097	0.842 ± 0.024	–2.731	1.107
3000	(1.354 ± 0.066)E–2	0.193 ± 0.026	2.800 ± 0.081	0.677 ± 0.051	–4.302	1.327
4000	(4.36 ± 0.53)E–3	0.261 ± 0.051	2.03 ± 0.15	0.51 ± 0.12	–5.434	1.653
5000	(1.83 ± 0.51)E–3	0.344 ± 0.070	1.49 ± 0.23	0.36 ± 0.21	–6.303	2.089

Table S39. Generalized Debye parameters for fitting of **3-Dy·2thf** ac data at 500 Oe and variable temperature.

T / K	τ / s	α	Δχ / cm^3 mol^{-1}	χ_S / cm^3 mol^{-1}	ln τ / ln s	$\sigma_{<\ln \tau>}$ / ln s
4	(1.03 ± 0.17)E–2	0.208 ± 0.042	1.55 ± 0.14	0.477 ± 0.017	–4.575	1.399
4.5	(4.58 ± 0.23)E–3	0.172 ± 0.023	1.317 ± 0.040	0.431 ± 0.012	–5.386	1.228
5	(2.418 ± 0.073)E–3	0.155 ± 0.017	1.177 ± 0.022	0.393 ± 0.011	–6.025	1.148
6	(8.34 ± 0.19)E–4	0.123 ± 0.013	0.969 ± 0.014	0.337 ± 0.010	–7.089	0.994
7	(3.64 ± 0.11)E–4	0.102 ± 0.018	0.824 ± 0.018	0.297 ± 0.015	–7.919	0.890
8	(1.825 ± 0.063)E–4	0.091 ± 0.018	0.720 ± 0.020	0.266 ± 0.019	–8.609	0.833
9	(9.93 ± 0.38)E–5	0.083 ± 0.018	0.638 ± 0.023	0.242 ± 0.023	–9.217	0.790
10	(5.66 ± 0.29)E–5	0.077 ± 0.022	0.571 ± 0.035	0.227 ± 0.034	–9.780	0.758
11	(3.25 ± 0.35)E–5	0.074 ± 0.040	0.514 ± 0.082	0.215 ± 0.083	–10.335	0.741
12	(1.84 ± 0.27)E–5	0.076 ± 0.046	0.46 ± 0.12	0.21 ± 0.12	–10.903	0.749
13	(9.9 ± 2.2)E–6	0.081 ± 0.069	0.41 ± 0.12	0.21 ± 0.12	–11.519	0.776

Table S40. Generalized Debye parameters for fitting of **3-Dy·2thf** ac data at 1000 Oe and variable temperature.

T / K	τ / s	α	Δχ / cm^3 mol^{-1}	χ_S / cm^3 mol^{-1}	ln τ / ln s	$\sigma_{<\ln \tau>}$ / ln s
4	(1.075 ± 0.082)E–2	0.166 ± 0.020	1.615 ± 0.070	0.4246 ± 0.0082	–4.533	1.198
4.5	(4.95 ± 0.12)E–3	0.149 ± 0.011	1.404 ± 0.021	0.3845 ± 0.0063	–5.309	1.119
5	(2.576 ± 0.046)E–3	0.140 ± 0.010	1.255 ± 0.014	0.3506 ± 0.0068	–5.962	1.074
6	(8.80 ± 0.15)E–4	0.114 ± 0.0099	1.027 ± 0.011	0.3020 ± 0.0079	–7.035	0.949
7	(3.840 ± 0.083)E–4	0.097 ± 0.012	0.869 ± 0.013	0.268 ± 0.011	–7.864	0.865
8	(1.910 ± 0.050)E–4	0.087 ± 0.014	0.756 ± 0.016	0.242 ± 0.015	–8.563	0.810
9	(1.035 ± 0.029)E–4	0.081 ± 0.014	0.670 ± 0.018	0.221 ± 0.017	–9.176	0.779
10	(5.86 ± 0.25)E–5	0.078 ± 0.018	0.597 ± 0.029	0.206 ± 0.029	–9.744	0.762
11	(3.33 ± 0.37)E–5	0.077 ± 0.041	0.536 ± 0.086	0.197 ± 0.087	–10.310	0.755
12	(1.89 ± 0.37)E–5	0.072 ± 0.074	0.47 ± 0.16	0.20 ± 0.16	–10.874	0.728
13	(1.04 ± 0.17)E–5	0.076 ± 0.053	0.420 ± 0.094	0.204 ± 0.096	–11.469	0.752

Table S41. Generalized Debye parameters for fitting of **3-Dy·2thf** ac data at 3000 Oe and variable temperature.

T / K	τ / s	α	Δχ / cm^3 mol^{-1}	χ_S / cm^3 mol^{-1}	ln τ / ln s	$\sigma_{<\ln \tau>}$ / ln s
2	(1.77 ± 0.63)E–2	0.249 ± 0.054	1.78 ± 0.32	0.480 ± 0.019	–4.037	1.596
2.25	(1.46 ± 0.32)E–2	0.229 ± 0.040	1.71 ± 0.19	0.465 ± 0.015	–4.228	1.497
2.5	(1.26 ± 0.20)E–2	0.216 ± 0.033	1.64 ± 0.14	0.451 ± 0.013	–4.377	1.436
3	(9.38 ± 0.79)E–3	0.194 ± 0.023	1.520 ± 0.070	0.4200 ± 0.0098	–4.669	1.331
3.5	(6.62 ± 0.30)E–3	0.173 ± 0.016	1.404 ± 0.036	0.3904 ± 0.0078	–5.017	1.234
4	(4.25 ± 0.10)E–3	0.149 ± 0.012	1.286 ± 0.019	0.3635 ± 0.0065	–5.462	1.119
4.5	(2.680 ± 0.041)E–3	0.1370 ± 0.0086	1.177 ± 0.011	0.3375 ± 0.0053	–5.922	1.062
5	(1.684 ± 0.030)E–3	0.124 ± 0.011	1.078 ± 0.012	0.3153 ± 0.0071	–6.387	0.999
6	(7.132 ± 0.087)E–4	0.1101 ± 0.0071	0.9308 ± 0.0073	0.2764 ± 0.0055	–7.246	0.930
7	(3.376 ± 0.068)E–4	0.096 ± 0.011	0.809 ± 0.012	0.248 ± 0.010	–7.994	0.859
8	(1.750 ± 0.042)E–4	0.087 ± 0.013	0.713 ± 0.014	0.224 ± 0.013	–8.651	0.813
9	(9.71 ± 0.28)E–5	0.080 ± 0.014	0.636 ± 0.018	0.207 ± 0.017	–9.240	0.774
10	(5.57 ± 0.17)E–5	0.077 ± 0.013	0.571 ± 0.021	0.194 ± 0.021	–9.795	0.755
11	(3.22 ± 0.38)E–5	0.074 ± 0.044	0.499 ± 0.087	0.184 ± 0.088	–10.345	0.742
12	(1.77 ± 0.23)E–5	0.077 ± 0.050	0.45 ± 0.10	0.18 ± 0.10	–10.940	0.757
13	(9.6 ± 2.0)E–6	0.081 ± 0.063	0.40 ± 0.11	0.19 ± 0.11	–11.553	0.776

8. References

1. Wang, F. T.; Najdzionek, J.; Leneker, K. L.; Wasserman, H.; Braitsch, D. M., A facile synthesis of imidotetraphenyldiphosphinic acids. *Synthesis and Reactivity in Inorganic Metal-Organic Chemistry* **1978,** *8* (2), 119-125.
2. Barbosa, G. A.; Neto, J. S. C.; Stoeberl, B. J.; Wisbeck, S.; Giese, S. O.; Yokaichiya, F.; Costa, D. d. S.; Barison, A.; Ribeiro, R. R.; Piovan, L.; Hughes, D.; Briganti, M.; Poneti, G.; Nunes, G. G.; Santana, F. S.; Soares, J. F., High-yield synthesis of heavy rare earth (iii) anhydrous solvates: known, new, and unexpected products. *Dalton Transactions* **2025,** *54* (20), 8251-8269.
3. Cea-Olivares, R.; Díaz-Vera, S.; López-Cardoso, M.; Tlahuext, H.; Vargas-Pineda, G.; Román-Bravo, P.; Jancik, V., The synthesis, spectroscopic characterization, and crystallographic study of the tetra-phenylimido-diphosphinates of Sc, Y, La, and Lu. *Journal of Molecular Structures* **2024,** *1297*, 136917.
4. Spek, A., PLATON SQUEEZE: a tool for the calculation of the disordered solvent contribution to the calculated structure factors. *Acta Crystallographica Section C* **2015,** *71* (1), 9-18.
5. Spek, A. L., PLATON SQUEEZE: a tool for the calculation of the disordered solvent contribution to the calculated structure factors. *Crystal Structure Communications* **2015,** *71* (1), 9-18.
6. Llunell, M.; Casanova, D.; Cirera, J.; Bofill, J.; Alemany, P.; Alvarez, S.; Pinsky, M.; Avnir, D., Program for the Stereochemical Analysis of Molecular Fragments by Means of Continuous Shape Measures and Associated Tools. *SHAPE, v1. 1b* **2005**, 1-35.
7. Coelho, A. A., TOPAS and TOPAS-Academic: an optimization program integrating computer algebra and crystallographic objects written in C++. *Applied Crystallography* **2018,** *51* (1), 210-218.
8. Rangarajan, S.; Beaumont, O. A.; Balakrishna, M. S.; Deacon, G. B.; Blair, V. L., Imidobis (tetraphenylphosphinato) lanthanoid (III) complexes: Synthesis by oxidative protolysis, and redox transmetallation/protolysis, structural studies and Hirshfeld surface analysis. *Inorganica Chimica Acta* **2025,** *574*, 122385.
9. Siiman, O.; Vetuskey, J., Synthesis of copper thio-and dithioimidodiphosphinates. Infrared and Raman spectra of imidodiphosphinates. *Inorganic Chemistry* **1980,** *19* (6), 1672-1680.
